# Supplementary material for: Defining good health and care from the perspective of persons with multimorbidity: results from a qualitative study of focus groups in eight European countries
Source: BMJ Open. 2018 Aug 30;8(8):e021072. doi: 10.1136/bmjopen-2017-021072 (PMC6119413; doi:10.1136/bmjopen-2017-021072)
Supplement: Supplementary data [file bmjopen-2017-021072supp001.pdf]

Supplementary files accompanying the manuscript:

Defining good health and care from the perspective of persons with multi-morbidity: Results from a qualitative study of focus groups in eight European countries

Box S.1: Extended protocol of the focus group

Introduction

- Purpose: to discuss what is important from the perspective of an individual with multiple health- and/or social problems in health/well-being and in care
- Overarching aim: the results of the discussion will be used to improve the care for persons with multiple health- and social problems
- Agenda explanation
- 'Rules'
    - No right / wrong answers, only differing points of view
    - Not necessary to agree with others, but must listen respectfully as others share their views
    - Talk to one-another, researchers only have role as moderator in guiding the discussion
    - Privacy / anonymity
    - The discussion will be recorded, and notes will be made
    - Turn off phones
- Informed consent
- Introduction of participants to one-another

Part 1: Defining good health/well-being and care

Step i):

- When you think about your health and well-being: try to complete this sentence / thought: "For me, being in great health means…"
- When you think about the care that you receive, have received, or may receive in the future, try to complete this sentence / thought: "I'd be really satisfied with all of the care / the overall care that I receive, if…"

**Write this question on a large white board / flip-over*

- Provide examples if participants are having trouble understanding what you mean:
    - …being able to work full-time. (health)
    - …being able to spend time with family and friends. (health)
    - …being able to live for many more years. (health)
    - …not going to the doctor so often. (health)
    - …my doctor took more time to explain things to me. (care)
    - …my partner would always be allowed to join me and discuss with the doctors as well. (care)
    - …my care plan was discussed with me. (care)
    - …I had one overall written care plan. (care)
    - …I always knew I could contact one person in case of questions. (care)

**The researcher needs to ask triggering and thought-provoking questions here.*

**The discussion should be about health and well-being in general, not about care specifically (yet).*

**Be careful not to provide too many examples and to steer the answers in one direction.*

**The researcher leading the discussion will be writing these thoughts out on a white board / flip-over.*

**The researchers need to allow the participants to reflect on one-another's ideas.*

Close the discussion with a final question: i.e., have we missed anything?

Step ii):

- Once it seems as though all participants have said what is important to them, the researcher will go through the points on the board, and with the help of the participants try to extrapolate what these things mean, in order to go from **specific** examples to **general** outcomes**.** For example:
    - "For me, being in good health means being able to work full-time", does this mean:
        - Participating in society?
        - Being able to support one-self financially? → Self-sufficiency
        - Having social contacts?
        - Having a daily structure?
    - "For me, being in good health means not going to the doctor so often", does this mean:
        - Having no acute health problems?
        - Having the ability to deal with minor health problems on your own? (self-management)
    - "I'd be really satisfied with all of the care / the overall care that I receive, if my doctor took more time to explain things to me", does this mean:
        - Good communication?
        - Shared-decision making?
        - More/enough time?
    - "I'd be really satisfied with all of the care / the overall care that I receive, if my partner could always be allowed to join me and discuss with the doctors as well.", does this mean:
        - Understanding your personal situation?
        - Involvement of the informal caregiver?
        - Good communication?

**The researcher needs to write key words that come forth on large notecards and place them in the centre of the table for all participants to be able to see.*
**The researcher needs to ask triggering and thought-provoking questions here – what do the points mentioned in step 1 really mean? What does it come down to? What would it mean for the person sitting next to you? How can you generalise this so that it is applicable for others in the room? Try to distinguish between individual opinions and actual group consensus.*
**In asking further questions, try to focus on multi-morbidity – how is this specific to you as a person with multiple health- and/or social problems, would this be different if you had a single disease?*
**For each initial statement, multiple cards can be made.*
**Close the discussion with a final question: i.e., have we covered all elements?*

Break

**Place cards from part one onto the table, sort them by 'triple aim'*
**Sort through original 77 cards, and add cards to the table from these (refrain from overlap, choose card from part one in words of participants over literature-cards)*

Part 2: Selecting most important concepts

- Instructions: "In the discussion during part 1 you've come up with points that are important for you when it comes to defining health and care. As you saw, we've been trying to write this into more general terms on these cards."
- In our research team we've also been looking at concepts that are often mentioned in research/literature as being important, we've added these to the table as well."

**Explain the new concepts in case anything is unclear.*

- "We'd like to ask each of you to look at all of these cards and write down for yourself which 10 are most important to you. This can be a mix of health/well-being, care, and cost cards."
- "Now we'd like you all to discuss your top 10 and try to see whether you can reach a group consensus, and make a top 10 of cards at the centre of the table."

**Ask a participant to first put his/hers down or read his/her list first, through discussion see whether some kind of consensus can be reached.*

Box S.2: List of literature-based outcomes used on cards in part two

Health / well-being

- Biomedical outcomes: e.g., blood sugar, blood pressure, lung function, kidney function
- Life expectancy
- Maintaining independence
- Activities of daily living: e.g. eating, bathing
- Independence from medical aids: e.g., wheelchair, oxygen
- Energy and fatigue
- Pain and discomfort
- Disability
- Physical mobility: e.g., being able to walk
- Work ability: being physically and mentally able to work
- Cognitive functioning: e.g., thinking, memory, concentration and learning
- Anxiety and depression
- Stress
- Worrying
- Listless: no spirit, apathetic, indifferent
- Self-esteem: a good feeling of one-self, self-respect
- Respect from others
- Social relationships: staying in contact with family/friends/acquaintances
- Maintaining social status: in terms of 'societal class', 'occupation', how you think others look at you in society.
- Societal participation: e.g., volunteer work, club memberships, community activities
- Social support: emotional, instrumental and financial assistance from the social network
- Loneliness
- Investing in future health and wellbeing
- Self-efficacy: belief in one's capacity to follow a needed or desired course of action
- Positive frame of mind, resilience: ability to maintain a positive perspective regarding the future, despite setbacks or change
- Coping
- Being able to deal with problems, losses, adverse events in an effective manner

• Health literacy: capacity to obtain, process, and understand information about illness and health<br>• Compliance/adherence to treatment<br>• Lifestyle: e.g., smoking, nutrition, physical activity, alcohol and drug use<br>• Feeling safe<br>• Enjoyment of life<br>• Maintaining dignity<br>• Living comfortably<br>• Caregiver burden
**Experience with care**
• Holistic assessment<br>• Shared decision-making<br>• Tailored care<br>• Individualised care planning<br>• Pro-active, prevention-oriented care<br>• Clear responsibilities and accountability<br>• Named coordinator<br>• Team work and collaboration between professionals<br>• Good communication<br>• Shared information between professionals and providers<br>• Continuity of care: pertains to continuity (e.g., follow-up, monitoring, stability, transfer) throughout time and between professionals and organisations)<br>• Confidence in knowledge and skills in professionals<br>• Respectful interaction between provider-patient: feeling of being treated with respect and being 'heard'<br>• Geographical access: e.g., facilities close to home<br>• Physical access: e.g., building is wheelchair accessible<br>• Timely access: e.g., no/short waiting lists<br>• Satisfaction with care process
**Costs**
• Service and support coverage: co-payments/deductible and financial access to services<br>• Travel and parking costs<br>• Loss of income – patient<br>• Loss of income - informal caregiver

Box S.3: Most important concepts in health/well-being, care, and costs per focus group participant per country (part 2 of the focus groups)

	**# / 58**	**Austria**	**Croatia**	**Germany[1]**	**Hungary**	**Netherlands**	**Norway**	**Spain**	**UK**
Health and well-being									
**Physical well-being**									
Energy & fatigue	**12**	4/7			1/6	4/7	2/7		1/6
Pain & discomfort	**4**	3/7							1/6
Biomedical outcomes	**3**	2/7			1/6				
Disability	**5**		2/7		1/6		1/7		1/6
Progression free health state*	**2**				2/6				
Physical mobility	**5**		1/7	1/6	1/6	1/7	2/7		
Activities of daily living	**7**		1/7	3/6	1/6	1/7			1/6
Life expectancy	**1**					1/7			
To be active*	**2**						2/7		
**Feeling safe**	**10**		1/7		3/6	3/7		3/6	
Discrimination*	**3**		3/7						
**Cognitive functioning**	**12**	4/7		1/6		2/7	1/7	3/6	1/6
**Independence**									
Being able to choose what you do / want to do*	**1**							1/6	
Moving freely around street *	**1**							1/6	
Independence from medical aids	**3**				1/6			1/6	1/6
Maintaining independence	**16**		2/7	5/6	1/6	1/7		1/6	1/6
Living comfortably	**5**	1/7		2/6		1/7			1/6
**Psychological well-being***	**3**			3/6					
Listlessness	**3**	2/7						1/6	
Loneliness	**2**	2/7							
Empathy*	**1**		1/7						
Stress/worrying	**7**				3/6		1/7		3/6
Anxiety & depression	**2**				1/6				1/6
**Enjoyment of life**	**16**	2/7		4/6	1/6	3/7	3/7		3/6
**Positive frame of mind, resilience**	**16**	2/7	3/7	2/6	2/6	1/7	2/7	2/6	2/6
Creativity*	**2**		2/7						
Investing in future	**1**							1/6	
Coping	**5**	1/7					2/7	1/6	1/6
Maintaining dignity	**9**	1/7			4/6			2/6	2/6
Self-esteem	**11**	1/7	1/7	3/6		3/7	2/7		1/6
Acceptance*	**4**			3/6		1/7			
Look out for possibilities*	**1**						1/7		
Hope	**1**						1/7		

	# / 58	Austria	Croatia	Germany[1]	Hungary	Netherlands	Norway	Spain	UK
**Social relationships & participation**									
Social relationships	**17**	3/7		5/6	2/6	4/7		2/6	1/6
Societal participation	**12**	1/7	3/7	3/6		3/7		1/6	1/6
Respect from others	**6**					2/7	2/7	1/6	1/6
To be taken seriously*	**3**						3/7		
Social support	**5**	1/7	1/7				2/7	1/6	
Being able to relate to other people without physical limits*	**1**							1/6	
Feeling useful*	**2**							2/6	
Get something off one's chest*	**2**	2/7							
(Maintaining good) social status	**5**	2/7		3/6					
Not being reduced to the disease*<br>To be regarded as whole human being*<br>Seeing past the outside/surface of a person*	**6**	1/7					 4/7<br>1/7		
Surrounding area takes care of someone's needs*	**1**	1/7							
Treatments compatible with work*	**1**	1/7							
Work ability	**6**		3/7		1/6	2/7			
Social expectations*	**1**		1/7						
Daily structure*	**4**			4/6					
Being able to plan*	**2**						2/7		
Burden for informal caregivers*	**3**			2/6			1/7		
**Self-management***	**7**			6/6		1/7			
Lifestyle	**3**	2/7							1/6
Compliance/adherence to treatment	**5**	1/7		2/6	1/6				1/6
Self-efficacy	**6**		2/7		1/6			1/6	2/6
Investing in future health & well-being	**4**		1/7	2/6		1/7			
Health literacy	**7**			5/6	1/6	1/7			
**Daily quality of life**	**1**						1/7		
Experience									
**Individualised care planning / tailored care**	**13**	1/7		4/6	1/6	2/7	4/7		1/6
**Holistic assessment/understanding**	**11**	1/7	1/7	4/6	2/6	2/7			1/6
**Good interactions providers-patients**									
Good communication	**14**	1/7	1/7	2/6		2/7	1/7	3/6	4/6
Encourage patients*	**1**							1/6	
Psychological support*	**2**			1/6		1/7			
Optimist reactions*	**1**							1/6	
Shared decision-making	**13**	2/7		4/6	1/6	1/7	1/7		4/6
Respectful interaction between provider-patient	**12**	2/7	1/7	5/6					4/6
Trust*	**7**			3/6		4/7			
Predictability of care service delivery* (good info)	**1**				1/6				
Flow of information*	**2**						2/7		

	# / 58	Austria	Croatia	Germany[1]	Hungary	Netherlands	Norway	Spain	UK
Being listened to and believed*	**2**								2/6
**Good collaborations providers**									
Shared information between providers*	**10**	2/7	1/7	1/6	2/6	2/7		1/6	1/6
Data transparency*	**3**			3/6					
Team work between providers	**10**		1/7		2/6		2/7	2/6	3/6
Solidary distribution between funds*	**1**	1/7							
Clear responsibility and accountability	**5**		1/7	3/6					1/6
Named coordinator	**7**		1/7	4/6		1/7	1/7		
**Continuity of care**	**9**		2/7	2/6	3/6	1/7		1/6	
Collaboration across sectors*	**5**			4/6					1/6
Coordination between primary care and hospitals*	**1**							1/6	
Prevent and avoid going to ER*	**2**							2/6	
Administrative barriers*	**1**		1/7						
Transferring of responsibility*	**1**		1/7						
Transfer-care*	**2**			1/6		1/7			
After-care*	**2**			1/6		1/7			
Systematic operating procedures in care*	**3**				3/6				
Utilisation of EMRs*	**2**			3/6	2/6	2/7			
Careful use of patient files / privacy	**2**			2/6					
**Confidence in knowledge and skills in providers**	**10**	1/7	2/7	3/6	2/6	1/7		1/6	
Education of health professionals*	**3**		3/7						
Importance and function of centres of excellence*	**1**		1/7						
Broader specialisations*	**1**						1/7		
**Access & availability**									
Physical access	**5**		1/7	1/6		2/7		1/6	
Timely access* (i.e., short waiting times)	**9**		2/7	1/6	2/6	1/7			4/6
Enough time* (provider for patient)	**8**	4/7			1/6	1/7		2/6	
Being seen by specialist when needed*	**2**							2/6	
Geographical access	**3**		1/7					1/6	1/6
Availability of treatment*	**3**		3/7						
Home care (available)	**1**			1/6					
Transport*	**1**			1/6					
**Avoid unnecessary treatment***	**2**	2/7							
**Pro-active, prevention-oriented, care**	**13**		1/7	1/6	4/6	4/7			3/6
**Patient-education and support (groups)**									
Integration of self-help groups*	**3**	3/7							
Importance of associations of patients*	**1**		1/7						
Good trainings offered*	**1**	1/7							
Medication help*	**5**			1/6		4/7			
Self-management support*	**1**					1/7			

	# / 58	Austria	Croatia	Germany[1]	Hungary	Netherlands	Norway	Spain	UK
Good info for patients on health	**1**						1/7		
Family support*	**3**		3/7						
**Bureaucratic hurdles***	**5**			4/6		1/7			
**Physical surroundings**									
Material conditions in institutions*	**1**		1/7						
**Macro-level**									
Non-cooperation with health policy makers*	**2**		2/7						
Importance of media pressure	**2**		2/7						
Learning from different countries' experiences*	**1**	1/7							
**Overall satisfaction with care process**	**8**	1/7		4/6	2/6			1/6	
Costs									
**Loss of income**									
Loss of income informal caregiver	**3**		1/7					2/6	
Loss of income patient	**7**	1/7	3/7		1/6			1/6	1/6
Overall costs in relation to income*	**1**					1/7			
**Service and support coverage**	**6**	2/7	1/7	1/6	1/6		1/7		
**Need for cost-benefit analysis***	**2**		2/7						
**Money follows the patient*** (bundled type payment that crosses all care level-borders)	**1**		1/7						
**Travel and parking costs**	**2**				1/6				1/6

Note: 1 = Group split into 2, so each time # out of 6 persons, each group discussed either health/well-being and costs or care and costs. Btw = between, Profs = professional care providers, EMRs = Electronic Medical Records, ER = Emergency Room. * Concepts/phrases that were novel and written on cards based on part 1 of the focus groups, and not on the basis of the a priori literature search.

Box S.4: Criteria used to come to a core set of outcomes to evaluate integrated care programmes

- **Cover the Triple Aim**: 1) improving population health/well-being and 2) patient experience, and 3) reducing cost (growth);
- **Measurable at an individual level** using PROMS and PREMS (this only applies to the first and second of the three aims);
- **Relevant for multi-morbidity** in different contexts and population groups;
- **Relevant for the 5P stakeholders: p**atients (persons with multi-morbidity), **p**artners (informal caregivers), **p**rofessionals, **p**ayers, **p**olicy makers.
- **Relevant across the** 17 selected **promising integrated care programmes**;
- **Timing of intervention effects**: the intervention effect (i.e., the impact of a programme on the outcome) in newly enrolled patients is likely to start occurring within the SELFIE evaluation period (6-18 months);
- The **outcomes are not redundant**, meaning that they are all necessary and not superfluous (e.g., one outcome does not overlap much with another outcome);
- **Preference independence**, i.e., the weight of one outcome can be elicited independently from the performance of another outcome;
- **Feasible to measure** performance.
