# Supplementary material for: Defining good health and care from the perspective of persons with multimorbidity: results from a qualitative study of focus groups in eight European countries
Source: BMJ Open. 2018 Aug 30;8(8):e021072. doi: 10.1136/bmjopen-2017-021072 (PMC6119413; doi:10.1136/bmjopen-2017-021072)
Supplement: Author's manuscript [file bmjopen-2017-021072.draft_revisions.pdf]

BMJ Open

BMJ Open is committed to open peer review. As part of this commitment we make the peer review history of every article we publish publicly available.

When an article is published we post the peer reviewers' comments and the authors' responses online. We also post the versions of the paper that were used during peer review. These are the versions that the peer review comments apply to.

The versions of the paper that follow are the versions that were submitted during the peer review process. They are not the versions of record or the final published versions. They should not be cited or distributed as the published version of this manuscript.

BMJ Open is an open access journal and the full, final, typeset and author-corrected version of record of the manuscript is available on our site with no access controls, subscription charges or pay-per-view fees (http://bmjopen.bmj.com).

If you have any questions on BMJ Open's open peer review process please email info.bmjopen@bmj.com

BMJ Open

# Defining good health and care from the perspective of persons with multi-morbidity: Results from focus groups in eight European countries

| | |
|---|---|
| Journal: | *BMJ Open* |
| Manuscript ID | bmjopen-2017-021072 |
| Article Type: | Research |
| Date Submitted by the Author: | 11-Dec-2017 |
| Complete List of Authors: | Leijten, Fenna; Erasmus Universiteit Rotterdam, Erasmus School of Health Policy & Management<br>Hoedemakers, Maaike; Erasmus Universiteit Rotterdam, Erasmus School of Health Policy & Management<br>Struckmann, Verena; Berlin University of Technology, Department of Health Care Management<br>Kraus, Markus; Institute of Advanced Studies<br>Cheraghi-Sohi, Sudeh; University of Manchester, NIHR Greater Manchester Patient Safety Translational Research Centre<br>Zemplenyi, Antal; Syreon Research Institute<br>Ervik, Rune; Uni Research Rokkan Centre<br>Vallve, Claudia; Consorci Institut D'Investigacions Biomediques August Pi i Sunyer (IDIBAPS)<br>Huic, Mirjana; Agency for Quality and Accreditation in Health Care and Social Welfare<br>Boland, Melinde; Erasmus Universiteit Rotterdam, Erasmus School of Health Policy & Management<br>Czypionka, Thomas; Institute of Advanced Studies<br>Rutten-van Mölken, Maureen; Erasmus Universiteit Rotterdam, Erasmus School of Health Policy & Management; Institute for Medical Technology Assessment, Erasmus University |
| Keywords: | PREVENTIVE MEDICINE, QUALITATIVE RESEARCH, person-centered, multi-morbidity, evaluation outcomes, integrated care |
| | |

SCHOLARONE™
Manuscripts

**Manuscript title:** Defining good health and care from the perspective of persons with multi-morbidity: Results from focus groups in eight European countries

**Authors**

Fenna Leijten[a], Maaike Hoedemakers[a], Verena Struckmann[b], Markus Kraus[c], Sudeh Cheraghi-Sohi[d], Antal Zemplényi[e], Rune Ervik[f], Claudia Vallvé[g], Mirjana Huiĉ[h], Thomas Czypionka[c], Melinde Boland[a], Maureen Rutten-van Mölken[a,i]*, on behalf of the SELFIE consortium

**Affiliations**

a) Erasmus University Rotterdam, Erasmus School of Health Policy & Management, Rotterdam, the Netherlands
b) Berlin University of Technology, Department of Health Care Management, Germany
c) Institute of Advanced Studies, Vienna, Austria
d) University of Manchester, NIHR Greater Manchester Patient Safety Translational Research Centre, Manchester, United Kingdom
e) Syreon Research Institute, Budapest, Hungary
f) Uni Research Rokkan Centre, Bergen, Norway
g) Consorci Institut D'Investigacions Biomediques August Pi i Sunyer (IDIBAPS), Barcelona, Spain
h) Agency for Quality and Accreditation in Health Care and Social Welfare, Zagreb, Croatia
i) Institute for Medical Technology Assessment, Erasmus University Rotterdam, Rotterdam, the Netherlands

*Corresponding author: Maureen Rutten-van Mölken: m.rutten@bmg.eur.nl / Tel: +31 10 4088559

For peer review only



For peer review only - http://bmjopen.bmj.com/site/about/guidelines.xhtml

For peer review only



For peer review only - http://bmjopen.bmj.com/site/about/guidelines.xhtml

**Abstract**

**Objectives**: The prevalence of multi-morbidity is increasing in many Western countries. Persons with multi-morbidity often experience a lack of alignment in the care that multiple health- and social care organisations provide. As a response, integrated care programmes are appearing. It is a challenge to evaluate such programmes and to choose appropriate outcome measures. To gain insight into meaningful outcomes, focus groups were held with persons with multi-morbidity in eight European countries, in which participants defined 'good health and well-being' and a 'good care process'.

**Methods**: In 2016, eight focus groups were organised with persons with multi-morbidity in: Austria, Croatia, Germany, Hungary, the Netherlands, Norway, Spain, and the UK (total N=58). Each focus group followed the same two-part procedure: 1) defining a) good health and well-being, b) a good care process and 2) group discussion on prioritising the most important concepts derived from part one and from a list extracted from the literature. Inductive and deductive analyses were done.

**Results**: Overall, the participants in all focus groups concentrated more on the care process than on health. Persons with multi-morbidity defined good health as being able to conduct and plan normal daily activities, having meaningful social relationships, and accepting and coping with the current situation. Absence of shame, fear, and/or stigma, being able to enjoy life, and overall psychological well-being were also important facets of good health. Being approached holistically by care providers was said to be vital to a good care process. Continuity of care and trusting professionals were also described as important. Across countries little variation in health definitions were found but variation in defining a good care process was seen.

**Conclusion**: A variety of health outcomes that entail well-being, social, and psychological facets and especially experience with care outcomes should be included when evaluating integrated care programmes for persons with multi-morbidity.




For peer review only - http://bmjopen.bmj.com/site/about/guidelines.xhtml

**Strengths and limitations of this study**

- Focus groups were held in 8 different European countries amongst persons with multi-morbidity.
- The qualitative, focus group, approach applied in this study allows persons with multi-morbidity to have a clear voice in the discussion on what meaningful evaluation outcomes are.
- Although different countries and multi-morbidities were included in this study, the applied methods do not allow for general statements and representativeness to be made.

For peer review only



For peer review only - http://bmjopen.bmj.com/site/about/guidelines.xhtml

## Introduction

With ageing populations in Western societies, there is an increasing prevalence of multi-morbidity, i.e., the co-occurrence of two or more health problems within one person at one time (van Oostrum et al., 2012; Barnett et al., 2012). Persons with multi-morbidity therefore often require care from different types of professionals. These professionals may work in different healthcare sectors (e.g., primary and secondary care) and may also work in social or community care. It is important that persons with multi-morbidity receive well-integrated care, in order to avoid the risk of fragmentation or overlap in the care received and interactions in treatment (Mair & May, 2014).

Integrated care is defined as structured efforts to provide coordinated, pro-active, person-centred, multidisciplinary care by two or more care providers that effectively communicate and collaborate. There are different integrated care programmes for multi-morbidity being implemented across Europe that may offer a solution to the aforementioned risks that this population faces (van der Heide et al., 2015; Struckmann et al., 2016; de Bruin et al., 2012; Hopman et al., 2017). The evidence-base of such programmes is still limited and findings are not yet wholly convincing (de Bruin et al., 2012; Hopman et al., 2017; Struckmann et al., 2016). Increasing this evidence-base is important for the durability, wider implementation, and more sustainable reimbursement/financing of such programmes. However, it can be questioned whether the current (economic) evaluation framework, in which costs per Quality Adjusted Life Years (QALYs) are calculated, provides sufficient insight into the broad range of outcomes that such integrated care programmes aim to improve. Integrated care programmes are complex interventions: they consist of various interacting components, target individuals but also groups and organisations, have a variety of intended outcomes, are amendable to tailoring through adaptation and learning feedback loops, and their effectiveness is impacted by the behaviour of those delivering and receiving the intervention (Craig et al., 2008). Common generic outcomes such as QALYs may not fully capture what these programmes are actually trying to achieve in persons with multi-morbidity. Their aims may go beyond the improvement of life expectancy and health-related quality of life, and include the improvement of well-being, the maintenance of independence, and increasing satisfaction with the care process.

In a time when the scarcity of resources is evident and evidence-based decisions on the spending of these resources are warranted, it is crucial to set up appropriate evaluations that can be used to convince decision-makers. Outcomes in the evaluations of complex care programmes often correspond at the higher level to the so-called 'triple aim': improving population health [and well-being], improving the patient's experience with care, and reducing cost [increase] (Berwick et al., 2008; Whittington et al., 2015). These higher level outcomes, however, can be interpreted in



For peer review only - http://bmjopen.bmj.com/site/about/guidelines.xhtml

different ways. Health can be defined as the absence of disease (Boorse, 1977), or a wider perspective can be applied whereby health is seen as the complete physical, mental, and social well-being beyond merely the absence of disease (WHO, 1946). More recent definitions turn health into a more active term, as the ability to adapt (Huber et al., 2011) and as a 'meta-capability' that can be used to attain human value (Venkatapuram, 2011). Similarly, the experience with care can include many different aspects, such as the extent of person-centeredness or to which care is proactive (e.g., two domains of the Patient Assessment of Chronic Illness Care (PACIC) instrument, Glasgow et al., 2005).

Different perspectives and approaches can be taken when operationalising the triple aim and meaningful outcomes of integrated care programmes. In the current study we aim to have persons with multi-morbidity define good health or well-being and a good care process, and identify what they find most important in each; thus focusing on two of the three 'triple aims'. We look at this from a cross-country perspective by conducting focus groups in eight European countries involved in the SELFIE research project on integrated care in multi-morbidity: the Netherlands, Austria, Croatia, Germany, Hungary, Norway, Spain, and the UK (see Box 1). Using a qualitative focus group approach encourages interaction between persons to take place that may allow for novel concepts and themes to arise.

**<<<Insert here Box 1:** Information on the SELFIE project>>>

For peer review only



For peer review only - http://bmjopen.bmj.com/site/about/guidelines.xhtml

## Methods

Focus groups were chosen because we were interested in the perspectives of persons with multi-morbidity themselves when it comes to health/well-being and care. The qualitative focus group methodology allows for novel concepts to arise, and interaction between persons can strengthen this process. The Consolidated criteria for reporting qualitative research (COREQ) checklist was used to structure this manuscript (Tong et al., 2007). Eight focus groups were conducted with persons with multi-morbidity, one in each SELFIE partner-country. The Dutch SELFIE team provided all other partners with a protocol on how to conduct and report on the focus groups, held a teleconference with each partner to discuss the protocol, and provided additional support throughout the process. All focus groups were organised between spring and fall of 2016.

### Recruitment

The goal was to recruit 6-8 persons for each focus group, with a mix of gender, age distribution, and types of multi-morbidity ($\geq$2 health and/or social problems) being desired. Participants were recruited via patient organisations (AT, DE, HR, NL, NO), medical professional organisations (HR), self-help groups (AT, DE), medical centres (AT, ES, HU), non-profit care organisations (e.g., Red Cross) (AT), and patient and public involvement groups (UK). We specified that we were searching for persons with multiple health- and/or social problems. Participants were also recruited via SELFIE national Stakeholder Advisory Boards (SAB) (AT, DE, HU, NL, NO). Each country has a SAB that reflects on SELFIE findings and consists of five stakeholder groups, '5Ps': Patients (persons with multi-morbidity), Partners (informal caregivers), Professionals, Payers, and Policy makers. At the time that the focus groups were being organised, a SAB meeting had just taken place in each country, with two to four persons with multi-morbidity present. These persons were reached out to for the focus groups and we tried to snowball via their networks.

Participants were reimbursed for their travel costs and in some countries a gratification was made available either as a token of appreciation or to aid recruitment (AT, DE, NL).

### Procedure

An extensive protocol was developed (see Supplementary file Box S.1) that was followed in each focus group. The first focus group was held by the Dutch team, who added 'lessons learned' to the protocol to aid the subsequent focus groups. Each country made a protocol in their own language to use during the actual focus group. The focus groups were all held in a meeting room in an office or clinical setting, with two to four researchers present, one being the chair and at least one taking extensive notes throughout the meeting. At least one of these researchers is a co-author on this



For peer review only - http://bmjopen.bmj.com/site/about/guidelines.xhtml

publication (FL, VS, MK, SCS, AZ, RE, CV, MiH, MB, MRM). The focus groups were also recorded. These researchers had a minimum of a bachelor's degree and experience/training in patient-contact and/or qualitative research.

The focus groups consisted of an introduction and two main parts: 1) defining a) good health and well-being, and b) a good care process, and 2) discussion on most important concepts and creating 'top-10' lists.

At the onset, the researchers welcomed everyone and introduced themselves, as only some researchers present had already been in touch with participants via telephone or email beforehand. The researchers stated their names, current position, and background expertise/occupation. Following this, in the introduction the researchers stated the purpose of the focus group: to discuss what is important from the perspective of an individual with multiple health- and/or social problems when it comes to health and care. The agenda of the focus group (introduction, part 1, part 2) was described. Next, the 'rules' of the focus group were introduced (see Supplementary File Box S.1) (e.g., respectful interaction, phones off). Participants were asked if they had any questions regarding the informed consent, which they had received beforehand, and had time to read this through if they had not done so already, and signed these. The recorder was then turned on and participants were asked to introduce themselves and briefly describe their multi-morbidities.

In part one of the focus group, participants were asked to define good health/well-being and a good care process. First, a discussion about good health/well-being was held, followed by a discussion about a good care process. We started by asking participants to complete the sentence "For me, being in great health means…" and "I'd be really satisfied with all of the care / the overall care that I receive, if…" Answers were discussed and written on flip-over boards. During this discussion a researcher asked triggering questions when needed and tried to focus the discussion on either health/well-being or care. After this, a researcher went through the statements on the flip-over board and the group tried to move from specific examples to general outcomes. These more general outcomes were highlighted / marked on the flip-overs and written on cards. Again a researcher needed to ask thought provoking questions: What do these original statements really boil down to? How can you expand this so that it counts for everyone in the room? Throughout both steps a researcher also could pose the question as to how this is especially relevant or different for multi-morbidity.

After part one, there was a 15-30 minute break. During the break, the researchers arranged the cards with concepts, or outcomes, mentioned during part one (e.g., on tables or white/magnet boards). They also sorted through the a priori made cards from the literature and added those *not* mentioned during part one to the display. These cards were made a priori by the Dutch team, and



For peer review only - http://bmjopen.bmj.com/site/about/guidelines.xhtml

distributed to each organiser. The outcomes on the a priori made cards stemmed from a large scientific literature review that had been conducted in the SELFIE project (Struckmann et al., 2017). All outcomes included in those publications were ordered according to the triple aim and, where possible, overlap was removed. This resulted in a list of 77 potentially relevant outcomes (51 health/well-being, 22 experience, 4 cost) (see Supplementary File Box S.2). Each focus group organiser had been asked to translate these outcomes to their respective languages and write them on cards, using different colours for each triple aim.

In part two, the researchers briefly explained to the group that the cards from part one were now on display, and that they also added novel cards on the basis of findings in the literature. These new concepts were mentioned one-by-one and, where unclear, explained. Participants were asked to look at all the concepts. They were asked to write down the ten concepts that were most important to them on a sheet of paper. Hereafter, a discussion was opened as to what was on everyone's 'top-10' list.

**Analyses**

All focus groups were recorded and extensive notes were made, pictures of flip-overs and cards were made, and the top-10 lists were collected. In two cases, verbatim transcriptions were also made (HR, NO). Reports were made of each focus group following a predefined template: structure of the focus group, recruitment, participant characteristics, reflection on the process, findings part 1, findings part 2, and conclusion/discussion.

Three analytical steps can be distinguished. First, throughout the reporting and summarising done for each focus group, the co-authors analysed and reflected on their respective focus group. This analysis was thus done by the researchers present at the focus group. Next, these reports were analysed independently by the first, second, and second-to-last author from the Dutch team. During this analysis step themes and priorities, corresponding to part one and two, were extracted from each focus group and these were compared across focus groups. Third, these themes and priorities were discussed amongst all co-authors, thus including the authors of the initial reports that were present at the focus groups. The analysis of part one was done applying an inductive approach, part two was done both inductively and deductively as concepts from the literature had been used here and concepts/outcomes were clustered and categorised. No specific qualitative analysis software was used in analysing the data.

The goal from the outset was to conduct one focus group per country, thus data saturation was not discussed a priori. As in most cases no verbatim transcripts were made, these were not returned to participants. However, in some cases, participants were emailed after the meeting to



For peer review only - http://bmjopen.bmj.com/site/about/guidelines.xhtml

thank them for their participation (DE, ES, HU, NL, NO, UK) and also in some cases were sent notes (NL). About one year after the focus groups, all participants were sent an update on the overall focus group results across countries.

**Ethics statement**

All participants signed an informed consent form. This form was developed by the Dutch team on the basis of the WHO informed consent for qualitative research (WHO). This was translated by each country into their respective languages. The informed consent consisted of information on the study, the purpose of the research, type of research, participant selection, voluntary participation, procedure, duration, potential risks, benefits, reimbursements, confidentiality, sharing of the results, right to refuse / withdraw, and contact information.



For peer review only

For peer review only - http://bmjopen.bmj.com/site/about/guidelines.xhtml

## **Results**

### Participant and focus group characteristics

In total 58 persons participated across the eight focus groups. Only in some cases specific persons not participate, this was because we could not get in touch with these persons, they had a conflicting agenda/schedule (e.g., holiday), were too busy, or in some cases had to cancel last minute due to illness or last minute (healthcare) appointments.

In all focus groups six or seven persons participated, except in Germany where 12 persons participated. During part two of the focus group in Germany, two subgroups were created to ease discussion. The mean age per focus group is presented in Table 1 below. The overall mean age was 65 years (range 31-86).

During the focus groups most frequently 3 researchers (min 2, max 4) were present, whereby 1 led [part of] the discussion and 1 took notes (total researchers present = 20, M/F = 4/16). The focus groups took 2.5 hours on average (min 2, max 3).

The majority of persons named three morbidities during the introduction of the focus groups (Table 1). Health problems were categorised according to the WHO 2010 International Classification of Diseases (ICD-10) (WHO, 2010). For an overview of the specific morbidities mentioned by each participant at the start of the focus groups see Table 2. Across countries, diseases of the circulatory system, musculoskeletal system, and endocrine disorders were most common morbidities amongst participants (Table 2). Specific examples of frequently mentioned diseases were high blood pressure, rheumatism, arthritis, and diabetes mellitus. It is of note that only in the UK mental health problems were explicitly named at the start of the focus groups (Table 2).

<<<**Insert here Table 1:** Focus group participant characteristics>>>

<<<**Insert here Table 2:** Morbidities of participants per country categorised according to the ICD-10>>

### Defining health and care (part 1)

*Defining health*

Across all focus groups the idea of good health being the ability to do **'normal' daily activities** was mentioned. This for example included activities such as going outside and undertaking activities (ES), being able to use a computer (UK), and doing physical activities within realistic reach:



For peer review only - http://bmjopen.bmj.com/site/about/guidelines.xhtml

"…Also being active is necessary for well-being. I was always very athletic nowadays only limited, but it works if you adapt your activities to your physical ability." (P2; AT)

*Response*: "That's true, though I cannot climb mountains anymore, but I can walk through different parks and I can still use the stairways." (P7; AT)

In three focus groups it was also said that especially in the case of multi-morbidity it is not only about doing such daily activities, but being able **to plan** them and **structure** them yourself (DE, NO, UK). This is related to having realistic expectations about what one can do and relates to acceptance. **Acceptance** was mentioned in terms of self-acceptance and acceptance by others (AT, DE, HU, NL). For example:

"Part of it is acceptance, I can do what I can do, and I should leave the other things." (P4; NL)

"How to handle the pain and the disease plays an important role. Integration of your diseases in your daily activities, accepting the pain and especially not feeling bad all the time although the diseases are permanent is a big step." (P11; DE)

"For me, I even feel good, if no additional things come. In my case, all of my conditions and problems that I was dealing with are considered as end-stage or final stage, and this is accepted. Therefore if my condition is not worsening, then I am fine." (P5, HU)

Daily activities are also linked to the desire to maintain **social relationships** and **participation** in society (AT, DE, ES, HR, HU, NL, NO).

"I want to be consciously active in society. With everything, doing what I want to do, being useful for others." (P7; NL)

"For me, good health means being psychosocially active…" (P1; HR) (i.e., having social relationships that are meaningful, being an active participant in everyday life)

In many countries good health was also associated with the **absence of shame, discrimination, fear, and/or stigma** (ES, HR, HU, NL, NO, UK). These feelings could relate to the wider public, the patient-provider, or the personal realm. For example, in Spain the examples of fear of walking alone and



For peer review only - http://bmjopen.bmj.com/site/about/guidelines.xhtml

being vulnerable or shame of being seen with medical apparatuses such as an oxygen machine were mentioned. In Hungary shame was mentioned in the patient-provider relation, e.g., not being able to keep to a diet. In Croatia these concepts were summarised as personal vulnerability. Fear of the future and not knowing how the disease trajectory will go was also discussed (HU, NL, NO). Doubts and worries about sharing updates on ones' diagnoses with family and the impact and burden that might have was mentioned (AT). In the Spanish focus group the discussion on fear and shame was heated, as persons dealt and coped with this issue differently. This relates to a point that also came up, of not only accepting the health problems, but also **coping**, being resilient, managing, and having responsibility for the diseases themselves, which may be perceived to be especially difficult in the case of multi-morbidity (DE, HU, NL, NO, UK).

> "Oh yeah, I think if you have multiple [diseases] it just adds to the whole workload really, how to cope with different things, oh yeah." (P1; UK)

The general idea of good health being defined as **feeling safe** also was discussed in various focus groups (ES, HU, NL, NO, UK). This can be seen as the result of being absent from the aforementioned negative states (e.g., fear). Feeling safe was also discussed in terms of trusting professionals (discussed more below). In Norway, feeling safe also extended to the economic realm:

> "…fear, fear for one day having to give up your work for example … if I don't manage any more, if I am unable to work anymore, then we do not have the economic resources to live here anymore, this was in my thoughts when the illness hits me and then fear…" (P2; NO).

Lastly, during the open discussion in part one, participants mentioned having a positive frame of mind, being able to enjoy life, and the importance of **psychological well-being** was mentioned in all focus groups.

> "I think psychological problems ought to be mentioned. Many people with chronic illnesses are also struggling psychologically. It could be because you have bad conscience, because you are dreading something, because you do not know if you will manage and suffer from performance anxiety". (P1; NO)

> "I want to enjoy my life, even though I've these diseases. That means, just being full of life." (P4; AT)



For peer review only - http://bmjopen.bmj.com/site/about/guidelines.xhtml

*Defining care*

When considering the wide array of themes discussed when defining health/well-being, it is not surprising that good care was defined by many persons as being approached as a whole person and **being treated holistically** (AT, NO, UK).

> "I wish that people treat me in a respectful manner, because it's true, I am sick, but the disease is not me. I don't want to be reduced to my diseases." (P2; AT)

> "So I would have wished for a doctor that, to put it this way, had the overview of the whole human being, that he shouldn't treat a heart disease just in isolation, you have another disease, and a third..." (P2; NO)

Persons explained that being approached holistically also means receiving **holistic support**, including informal caregiver support (HR, NL, NO, UK), good information provision, and especially **emotional and psychological support.** Participants mentioned that support should take the form of more extensive, easily accessible, and timely information on the health problems at hand and medication, but also psychological support, support with self-management, and self-help groups for example (AT, DE, ES, HR, HU, NL). Concerning emotional and psychological support specifically, this is needed even when this is not the 'main' problem at hand (AT, ES, HR, NL, UK). For example, in the Netherlands participants mentioned that in the case of multi-morbidity it takes time to accept the new health problems that arise on top of existing health problems and that there should be support for this adjustment period. In the UK a disconnection between the patient- and provider's priorities was seen, as it was mentioned that the healthcare professionals does do not focus on the mental health issues, when they should:

> "Yeah, for me I'd like the mental health to be bigger…for me all my healthcare professionals see the [physical disease] as the big thing with me, I don't, I see my depression as the big thing because that's what affects me day to day." (P4; UK)

> "What I see is that there's no psychological aid. When you are told you are a chronic patient and you have to take a drug all your life, and that this is for all your life, some people are depressed, needs psychological aid…" (P1; ES)



For peer review only - http://bmjopen.bmj.com/site/about/guidelines.xhtml

For peer review only

A wider theme discussed was **trust in professionals and the system**. This is in part linked to the emotional and psychological support, whereby persons felt that two-way trust is needed in the relationship between the patient and provider so that psychological issues can be discussed, the provider really listens, and so that they can embark on the care process together (AT, NL, NO, UK). Related to this, respectful interaction between provider and patient was also often mentioned (AT, DE, NL, NO, UK, HU). Specifically in the UK, the direction of trust was mentioned with participants saying that they needed to be 'believed' by the professionals (P2, P4, UK).

Trust also pertained to being able to rely on the provider in being able to help, based on their skills and knowledge (DE, ES, HR, NL, NO, UK). Participants spoke of having a 'prepared' care provider to talk to, and trusting their expertise and education.

> "Yeah, but there are difficulties with medication when you've got multiple things..." (P5; UK)
> *Response*: "Oh yeah, but you'd expect your GPs to keep on top of that, it is debatable as to whether that actually happens or not." (P1; UK)

In Hungary, Germany, and the Netherlands the trust also pertained to the professionals in general:

> "Trust in the medical world, well I'm sceptical about that, a lot of things happen around you, that you think, did they not see that?... Yeah, then you lose faith/trust because of the things that happen to you…" (P2; NL)

> "If I visit my doctor, I wish I could reproduce what he is doing there, which services are provided and I would like to sign for them. I want to be more informed about what is done. This way, I often cannot trust my physicians." (P8; DE)

As may be especially relevant in multi-morbidity, issues surrounding **continuity of care** were mentioned in all focus groups. This pertained to clear responsibilities, a clear contact point, transfer and 'after care', good communication, and good collaboration and teamwork. These points about continuity often also related to **sharing information or medical records** between providers and organisations (DE, ES, HR, HU, NO, NL).

> "The problem is the coordination between the primary health centres with the reference hospitals. The doctor at the hospital should see all the information, and the family doctor as well…" (P5; ES)



For peer review only - http://bmjopen.bmj.com/site/about/guidelines.xhtml

"I would expect that all the institutions and all the GPs in this city would be connected to the same electronical [IT] system. In this case I would not need to go with all those stacks of papers when I see a doctor. (...) There should be a system which can be seen by everyone and not only by some particular segments of the care." (P1, HU)

**Cross-country comparisons in defining good health and care (part 1)**

Little variation was seen between countries when defining health (part 1). Themes surrounding the definition of a good care process differed more between countries. Although aspects of access and availability were mentioned in all countries, this concept took a different form between the countries. In some cases this pertained to timely care in terms of waiting lists and time (ES, HU, NL). In Hungary, however, this specifically had to do with the lack of information on the waiting times. Patients emphasized that waiting times were more acceptable if patients knew in advance how long they had to wait.

"The worst thing is waiting… They could calculate an order with some gap in between. There are problems with the information sharing" (P6; HU)

Access in the form of time also referred to professionals having enough time for persons with multi-morbidity in Germany, Hungary, and Norway.

Availability also had different definitions per country – in Croatia, Germany, and the UK this was treatment, care, and provider availability and the freedom to choose between them. In Norway, Spain, and the UK this was also geographical- availability and access.

Several themes were only brought up in part one by certain countries. Namely, bureaucracy or the reduction of the burden thereof was mentioned in Germany and the Netherlands, for example with regard to care services that fall within our outside of the insurance package.

"There are persons who are physically not able to walk to their physiotherapist anymore. In fact they are in need of transport, but you have to apply for transportation for every single therapeutic unit. These are very difficult circumstances due to too much bureaucracy. This is disastrous…" (P9; DE)

In Austria participants discussed the need for more self-help and support groups. In contrast, in the Netherlands this was mentioned as a positive aspect already present and available. Participants in



For peer review only - http://bmjopen.bmj.com/site/about/guidelines.xhtml

Austria were aware that these things exist in other countries, and felt it was missing in their own context.

Lastly, physical surroundings in care provision were mentioned in Hungary that were related to cleanliness, enough personal space, and enough seats.

> "Because if we consider our life or our homes, we do not like if it is dirty and messy. In a good facility there should be cleanliness, order and discipline." (P2; HU)

> "At the … [department] patients need to wait in a very narrow corridor with one row of chairs. There are about three times more patients than chairs. (P5; HU)

Furthermore, in Hungary systematically organized operating procedures in care was an important requirement mentioned by multiple patients. This is made apparent through the fact that the care process in hospitals is sometimes unreasonably long, the staff is in rush, patients do not get clear instructions on the next steps of the procedure, there are redundant diagnostic test, and information is not shared appropriately among professionals.

**Most important concepts (part 2)**

In all focus groups, in part 2, participants were asked to make a top-10 list of most important concepts. These concepts were those identified in part 1 and supplemented with 'missing' concepts from the literature (see Supplementary File Box S.2). These top-10 lists were discussed. There was large variation within focus groups (i.e., countries) as to what concepts were deemed most important and on persons' top-10 lists. In Supplementary File Box S.3 all concepts written on persons' top-10 lists are presented, including those from the literature and 'novel' concepts derived upon during part 1 of the focus groups.

In Table 3 below an overview of the most frequently mentioned concepts is presented. These are concepts written on the top-10 lists of at least 10 persons (out of the 58 participants in total) across all countries. The health and well-being concepts social relationships, a positive frame of mind or resilience, enjoyment of life, and maintaining independence were most frequently noted by participants on their top-10 lists. A positive frame of mind or resilience, was mentioned by at least one person in each countries' focus group. Many facets pertaining to good interactions between care providers and persons with multi-morbidity were on the top-10 lists of participants, such as good communication, shared decision-making, and respect. Furthermore, individualised care planning and a holistic assessment and understanding of the problems at hand were aspects of the



For peer review only - http://bmjopen.bmj.com/site/about/guidelines.xhtml

care process also frequently on the top-10 lists. Lastly, pro-active, prevention-oriented, care was found important by many participants. As can be seen, there is great variety in concepts that participants put on their top-10 lists, both between and across countries. Furthermore, some phrases and words are quite specific whereas others are broad; participants were free to determine at what conceptual level they wrote their top-10 outcomes.

**<<<Insert here Table 3:** Most frequently listed outcomes on the 'top-10' lists of focus group participants across the eight focus groups>>>

For peer review only



For peer review only - http://bmjopen.bmj.com/site/about/guidelines.xhtml

## Discussion

### Main findings

Participants defined good health and well-being in terms of being able to conduct 'normal' daily activities, being able to plan and structure these, and having social relationships and participating in society. Acceptance by oneself and by others, and coping with, one's current health situation was deemed an aspect of good health. Absence of shame, discrimination, fear, and/or stigma from the public, care providers and oneself, and on the other end feeling safe and psychological well-being were also facets of good health. Social relationships, resilience, enjoyment of life, and maintaining independence were considered the *most* important aspects of good health across participants from all countries. A good care process was defined as one whereby persons are approached and supported holistically, with specific attention for emotional and psychological support, there is confidence and trust in professionals and the system, continuity of care is guaranteed, and that information is shared and accessible within a reasonable time. Concepts deemed *most* important were good communication, shared decision-making, and respect between care provider and the person with multi-morbidity, as well as individualised care planning and pro-active, prevention-oriented, care. Little cross-country variation in health themes were found, however, in defining care differences did exist, for example in terms of the exact type of access referred to (e.g., geographical, timely).

### Interpreting findings

Several themes brought up during the focus groups were explicitly mentioned to be more relevant for persons with multi-morbidity; this was especially the case when defining care. One such concept was enough time: in Norway persons mentioned that it is difficult when their issues are not immediately visible for a care provider, and that they need time to explain the multiple problems at hand. Also confidence in providers' skills are related to their needing to address or at least be aware of multiple problems and often multiple medications, some of which may go beyond their specialist area of expertise. Also the importance of well-coordinated and smooth transitions (i.e., continuity of care) is particularly so for persons with multi-morbidity, who often cross provider-, organisation- and sector boundaries throughout their care trajectories. In the realm of continuity of care, in many focus groups sharing information for example via shared electronic medical records was explicitly mentioned. It is of note that issues surrounding privacy were not mentioned by participants, although EMRs are in most cases not in place yet due to issues at a wider system level.

Aspects especially to do with defining care seemed relatively unique and extra relevant for multi-morbidity. However, there was a large degree of overlap in the concepts mentioned in



For peer review only - http://bmjopen.bmj.com/site/about/guidelines.xhtml

defining health in the current focus groups and the existing definitions presented in the introduction by the WHO, Huber and Vankatapuram, and similar studies conducted on defining health. Namely, absence of shame, discrimination, fear, and/or stigma, feeling safe, psychological well-being, and social relationships and participation especially overlap with the mental and social well-being aspects of the 1946 WHO definition of health (WHO, 1946). The importance of mental health outcomes has also been recently found in a study amongst a UK general population on what aspects of the EuroQol 5-dimensions (EQ-5D) are currently missing or receive too little attention, i.e., mental health (Shah et al., 2017). This is also in line with a study by Ebrahimi and colleagues (2012) amongst frail elderly where being able to 'master' daily life (e.g., coping and acceptance) and being happy and satisfied with life (e.g., enjoyment of life) were described as characteristics of health.

Other concepts found in the current focus group relate more to the definition by Huber and colleagues of health as the ability to adapt, i.e., acceptance, coping, resilience (Huber et al., 2011). Also overlap was seen with our finding on the ability to conduct normal daily activities and the aforementioned study on frail elderly where the ability to live the routine life persons are accustomed to was identified as an aspect of health (Ebrahimi et al., 2012). Lastly, especially Venkatapuram's definition of health as the capability to attain human value, seems to overlap with the general idea of the ability to participate in normal daily activities and social participation (a means to attain human value) and enjoyment of life as mentioned throughout the current focus groups (Venkatapuram, 2011).

It was noticeable that, across all focus groups, aspects relating to care were already mentioned in defining good health. It appeared that persons could not always clearly distinguish the two. This might be due to the fact that persons more easily can identify these care-related factors as 'changeable'. When exploring which aspects of health/well-being and care were most important in part two of the focus groups, we saw across countries that these overlapped with those mentioned during the first part of the discussions but also that often concepts were included from the literature-cards. As these were introduced and explained after the break, participants reacted positively to them. This indicates conceptual overlap in what is currently being assessed in evaluations, and thus found in the literature a priori, and what participants of our focus groups found important (Struckmann et al., 2017).

**Strengths & limitations**

Several limitations in the current study should be mentioned. Only in the UK mental health problems were explicitly mentioned by participants when morbidities were named at the start of the focus groups. This, however, did not seem to have any effect on our findings, as attention for psychological



For peer review only - http://bmjopen.bmj.com/site/about/guidelines.xhtml

health was deemed an important outcome across focus groups. The difference in explicitly naming mental health problems may relate to culturally-related stigma issues. For example, throughout the Dutch focus group it became apparent that depression issues were also present amongst participants. Also in the German focus groups mental health problems, such as depression, were mentioned as 'side effects' of other health problems.

It was not the goal of the current study to attain a representative sample of participants per country. Participants in some countries may, for example, be the more 'involved' patients. As such they are involved in research more often and may be more familiar with the topics discussed in the focus groups than an average patient. However, considering that our findings are largely in line with expectations based on previous studies, we do not feel that their perceptions of what good health and care is, and priorities therein, systematically differ from other patients.

Initially, the goal of the second part of the focus groups was to reach a group 'top-10', in which participants would discuss their individual lists and try to create a group list. However, due to time restrictions this was not possible. Thus the results of part two reflect each individuals' opinion. We opted to present findings in the results section mentioned by ten or more persons across the focus groups, but this is a relatively arbitrary cut-off point, and the full findings are shown in the Supplementary File Box S.3. The total number of cards from which participants could select concepts from for their top-10 list differed in size depending on from which country they are from. The level of specificity in concepts also differs greatly, for example the overall satisfaction with care process is mentioned, as well as the specific facet that a provider has enough time to listen to a patient (see Supplementary File Box S.3). Quantifying such a qualitative process is thus to be done cautiously. In the Supplementary File Box, concepts are clustered into broader concepts, there are of course different definitions and interpretations for terms, and as many concepts are all interrelated a level of subjectivity is introduced here.

We consider it a strength of the current research that the participating countries in SELFIE differ in geographical size, population [density], culture, and welfare and health care systems – providing a wide scope. Furthermore, it is a main strength that persons with multi-morbidity were so actively approached and included in the current study and in the wider SELFIE study, introduced in Box 1. As will be described below, the results of these focus groups form the predominant input for the core set of outcomes to be included in the SELFIE evaluations of 17 integrated care programmes: participants were given a direct voice and greatly valued this. Another main strength of the current study and great addition to the literature is the fact that we asked participants in our focus groups to not only reflect on health, but also on experience with care. In situations whereby health improvements, especially in the traditional physical and clinical health indicators, are infeasible,

For peer review only



For peer review only - http://bmjopen.bmj.com/site/about/guidelines.xhtml

focusing on their having a satisfactory and even positive experience throughout the care process becomes more important. And, as was seen in the current study, experience with care is often inextricably linked to the definition of health. Although we did not explicitly incorporate costs in the first part of the focus groups, and thus predominantly focus on two of the three 'triple aims', participants did mention several cost concepts, also in prioritising. In the future, it would be interesting to also have an explicit discussion on what role costs play in, especially, experiences with care.

**Next steps**

When evaluating integrated care programmes for multi-morbidity, a wide array of health- and care outcomes should be included. Few aspects related to physical functioning, a traditionally used outcome, were found on the top-10 lists of importance, i.e., only 'energy and fatigue' was mentioned by more than 10 persons across all focus groups. Instead, more well-being, social, and psychological facets of health are important and should thus be included in evaluations. This finding overlaps with current trends in conducting more comprehensive evaluations. To this end, in SELFIE a core set of outcomes for the evaluation of 17 promising integrated care programmes for multi-morbidity was developed that places a lot of focus on such outcomes. The core set consists of the following outcomes: physical functioning, psychological well-being, social relationships & participation, enjoyment of life, resilience, person-centeredness, continuity of care, and total health- and social care costs. This core set was developed on the basis of four sources, of which the focus groups described in the current article had the most prominent influence. The other three sources were: i) findings from a literature review conducted to develop a framework on integrated care for multi-morbidity and to identify existing programme evaluations (Struckmann et al., 2017), ii) (inter)national stakeholder advisory board discussions in the eight SELFIE partner countries with Patients, Partners, Professionals, Payers, and Policy makers, and iii) the aims of the aforementioned 17 programmes and current indicators included in these programmes.  A set of criteria was used to come to this list (see Supplementary File Box S.4), and some concepts mentioned in the focus group were not included in the core set, because these did not meet these criteria. This is the case for the more system- and cultural-wide concepts, such as absence of public stigma, which is cannot be used to assess the performance of one specific care programme.

**Conclusion**

A variety of health outcomes that entail well-being, social, and psychological facets should be included when evaluating integrated care programmes for persons with multi-morbidity. Even more



For peer review only - http://bmjopen.bmj.com/site/about/guidelines.xhtml

imperative is that experience with care outcomes be included in such evaluations that tackle the complexity of multi-morbidity care provision. In conclusion, important next steps include moving towards harmonising evaluation frameworks and the specific indicators used in such evaluations.

For peer review only

**Funding statement**

The SELFIE project has received funding from the European Union's Horizon 2020 research and innovation programme under grant agreement No 634288. The content of this publication reflects only the SELFIE groups' views and the European Commission is not liable for any use that may be made of the information contained herein.

**Data sharing statement**

The recordings and available verbatim transcripts will not be made available, to protect the anonymity of participants. Upon request to the corresponding author, we may consider in collaboration with the respective SELFIE-partner, whether a country-specific focus group report can be shared. Other findings from the overarching SELFIE study can be found on the SELFIE website (www.selfie2020.eu).

**Competing interests**

None of the involved co-authors had a competing interest.

**Author's contribution**

FL, MB, and MRM drafted the focus group procedure document and analysis instruction document, and organised and were present at the Dutch focus group. VS, MK, SCS, AZ, RE, CV, MiH, and TC were involved in the translation and organisation of the focus groups in their respective countries and in the analysis of their focus group and reporting thereof. FL, MB, and MRM analysed the Dutch group; FL, MaH, MB, and MRM analysed all focus group reports and conducted the overarching analyses. FL, MaH, and MRM drafted the manuscript; all co-authors critically reviewed and contributed to the manuscript, e.g., by appraising themes and providing fitting quotes).

For peer review only

**References**


Barnett K, Mercer SW, Norbury M, et al. Epidemiology of multimorbidity and implications for health care, research, and medical education: a cross-sectional study. Lancet. 2012;380(9836):37-43.

Berwick DM, Nolan TW, Whittington J. The triple aim: Care, health, and cost. Health Aff. 2008;27(3):759-769.

Boorse C. Health as a theoretical concept. Philosophy of Science. 1977;44(4):542-573.

de Bruin SR, Versnel N, Lemmens LC, et al. Comprehensive care programs for patients with multiple chronic conditions: A systematic review. Health Policy. 2012;107(2-3):108-145.

Craig P, Dieppe P, Macintyre S, et al. Developing and evaluating complex interventions: the new Medical Research Council guidance. BMJ. 2008;337:a1655.

Ebrahimi Z, Wilhelmson K, Moore CD, et al. Frail elders' experiences with and perceptions of health. Qual Health Res. 2012;22:1513-1523.

Glasgow RE, Wagner EH, Schaefer J, et al. Development and validation of the Patient Assessment of Chronic Illness Care (PACIC). Med Care. 2005;43:436-444.

van der Heide I, Snoeijs S, Melchiorre MG, et al. Innovating care for people with multiple chronic conditions in Europe: An overview. Utrecht, the Netherlands: ICARE4EU, NIVEL, 2015.

Hopman P, de Bruin SR, Forjaz MJ, et al. Effectiveness of comprehensive care programs for patients with multiple chronic conditions or frailty: a systematic literature review. Health Policy. 2016: [Epub ahead of print].

Huber M, Knottnerus JA, Green L, et al. How should we define health? BMJ. 2011;343:d4163.

Mair FS & May CR. Thinking about the burden of treatment. BMJ. 2014;348:g6680.

van Oostrum SH, Picavet HSJ, van Gelder BM, et al. Multimorbidity and comorbidity in the Dutch population – data from general practices. BMC Public Health. 2012;12:715.

Shah KK, Mulhern B, Longworth L, Janssen MF. Views of the UK general public on important aspects of health not captured by EQ-5D. Patient. Online first, 2017. DOI: 10.1007/s40271-017-0240-1

Struckmann V, Leijten FRM, van Ginneken E, et al. Relevant models and elements of integrated care for multi-morbidity: results of a scoping review. Health Policy. *Submitted* (2017).

Tong A, Sainsbury P, Craig J. Consolidated criteria for reporting qualitative research (COREQ): a 32-item checklist for interviews and focus groups. Int J for Qual Health Care. 2007;19:349-357.

Venkatapuram, S. Health Justice. Cambridge: Polity Press, 2011.

Whittington JW, Nolan K, Lewis N, et al. Pursing the Triple Aim: The first 7-years. Milbank Q. 2015;93(2):263-300.

World Health Organization. WHO Constitution. 1946.

World Health Organization. Informed Consent Form Template for Qualitative Studies.


For peer review only



For peer review only - http://bmjopen.bmj.com/site/about/guidelines.xhtml

World Health Organization (WHO). International Classification of Diseases (ICD-10). Geneva: WHO; 2010.

For peer review only



For peer review only - http://bmjopen.bmj.com/site/about/guidelines.xhtml

## Tables & Figures

**Box 1:** Information on the SELFIE project

**SELFIE** (Sustainable intEgrated chronic care modeLs for multi-morbidity: delivery, FInancing, and performancE) is a Horizon2020 funded EU project that aims to contribute to the improvement of person-centred care for persons with multi-morbidity by proposing evidence-based, economically sustainable, integrated care programmes that stimulate cooperation across health and social care and are supported by appropriate financing and payment schemes. More specifically, SELFIE aims to:

- Develop a taxonomy of promising integrated care programmes for persons with multi-morbidity;
- Provide evidence-based advice on matching financing/payment schemes with adequate incentives to implement integrated care;
- Provide empirical evidence of the impact of promising integrated care on a wide range of outcomes using Multi-Criteria Decision Analysis;
- Develop implementation and change strategies tailored to different care settings and contexts in Europe, especially Central and Eastern Europe.

Seventeen promising integrated care programmes for persons with multi-morbidity are being evaluated in SELFIE using MCDA and a common set of core outcomes as well as programme-type specific outcomes. The latter depend on whether a programme is i) a population health management programme, ii) a programme targeting frail elderly, iii) a programme targeting persons with problems in multiple life domains, or iv) an oncology or palliative care programme.

The SELFIE consortium includes eight organisations in the following countries: the Netherlands (coordinator) (NL), Austria (AT), Croatia (HR), Germany (DE), Hungary (HU), Norway (NO), Spain (ES), and the UK. (www.selfie2020.eu) [Grant Agreement No 634288]

**Table 1:** Focus group participant characteristics

| | **# (M/F)** | **Mean age (Min-Max)** | **Mean number of morbidities** |
|---|---|---|---|
| **Austria (AT)** | 7 (5/2) | 72.9 (62-84) | 2.3 |
| **Croatia (HR)** | 7 (4/3) | 51.7 (31-69) | 3 |
| **Germany (DE)** | 12 (4/8) | 62.4 (37-78) | 3.9 |
| **Hungary (HU)** | 6 (1/5) | 64.5 (47-78) | 3 |
| **Netherlands (NL)** | 7 (5/2) | 66.3 (53-75) | 5 |
| **Norway (NO)** | 7 (2/5) | 65.4 (42-76) | 3.1 |
| **Spain (ES)** | 6 (5/1) | 70.2 (60-81) | 2.7 |
| **United Kingdom (UK)** | 6 (4/2) | 68.8 (58-86) | 4 |
| **Total** | 58 (30/28) | 65.3 (37-86) | 3.4 |



For peer review only - http://bmjopen.bmj.com/site/about/guidelines.xhtml

**Table 2:** Morbidities of participants per country categorised according to the ICD-10

| | **Total** | **Austria** | **Croatia** | **Germany** | **Hungary** | **Netherlands** | **Norway** | **Spain** | **UK** |
|---|---|---|---|---|---|---|---|---|---|
| **Neoplasms** | 8 | 3 | 1 | 2 | | 1 | | 1 | |
| Diseases of the **blood** and disorders involving the immune mechanism | 3 | | 2 | | | | 1 | | |
| **Endocrine**, nutritional, and metabolic disease | 31 | 2 | 1 | 6 | 5 | 5 | 4 | 3 | 5 |
| **Mental** and **behavioural** disorders | 6 | | | | | | | | 6 |
| Diseases of the **nervous** system | 20 | 1 | 2 | 12 | 2 | | 1 | | 2 |
| Diseases of the **eye** and adnexa | 7 | | | | 1 | 6 | | | |
| Diseases of the **ear** and mastoid process | 5 | | | 3 | | | 1 | | 1 |
| Diseases of the **circulatory** system | 44 | 1 | 5 | 12 | 8 | 7 | 4 | 4 | 3 |
| Diseases of the **respiratory** system | 17 | | 1 | | | 6 | 4 | 6 | |
| Diseases of the **digestive** system | 10 | 1 | 1 | 1 | 1 | 2 | 1 | 1 | 2 |
| Diseases of the **skin** and subcutaneous tissue | 3 | 1 | 1 | | | | | | 1 |
| Diseases of the **musculoskeletal system** and connective tissue | 36 | 6 | 6 | 9 | 1 | 4 | 5 | 1 | 4 |
| Diseases of the **genitourinary** system | 4 | | | | | 4 | | | |
| **Congenital malformations**, **chromosomal abnormalities** | 1 | 1 | | | | | | | |
| Symptoms, signs, **abnormal clinical and lab findings**, **not else classified** | 2 | | | 2 | | | | | |
| **Injury**, **poisoning** and other consequences of external causes | 1 | | | | | | 1 | | |

Note: ICD-10: International Statistical Classification of Diseases and Related Health Problems, 10th revision.

For peer review only



For peer review only - http://bmjopen.bmj.com/site/about/guidelines.xhtml

**Table 3:** Most frequently listed outcomes on the 'top-10' lists of focus group participants across the eight focus groups

| | **# / 58** | **Austria (AT)** | **Croatia (HR)** | **Germany[1] (DE)** | **Hungary (HU)** | **Netherlands (NL)** | **Norway (NO)** | **Spain (ES)** | **United Kingdom (UK)** |
|---|---|---|---|---|---|---|---|---|---|
| *Health and well-being* | | | | | | | | | |
| Energy & fatigue | **12** | 4/7 | | | 1/6 | 4/7 | 2/7 | | 1/6 |
| Feeling safe | **10** | | 1/7 | | 3/6 | 3/7 | | 3/6 | |
| Cognitive functioning | **12** | 4/7 | | 1/6 | | 2/7 | 1/7 | 3/6 | 1/6 |
| Maintaining independence | **16** | | 2/7 | 5/6 | 1/6 | 1/7 | | 1/6 | 1/6 |
| Enjoyment of life | **16** | 2/7 | | 4/6 | 1/6 | 3/7 | 3/7 | | 3/6 |
| Positive frame of mind, resilience | **16** | 2/7 | 3/7 | 2/6 | 2/6 | 1/7 | 2/7 | 2/6 | 2/6 |
| Self-esteem | **11** | 1/7 | 1/7 | 3/6 | | 3/7 | 2/7 | | 1/6 |
| Social relationships | **17** | 3/7 | | 5/6 | 2/6 | 4/7 | | 2/6 | 1/6 |
| Societal participation | **12** | 1/7 | 3/7 | 3/6 | | 3/7 | | 1/6 | 1/6 |
| *Experience* | | | | | | | | | |
| Individualised care planning / tailored care | **13** | 1/7 | | 4/6 | 1/6 | 2/7 | 4/7 | | 1/6 |
| Holistic assessment/understanding | **11** | 1/7 | 1/7 | 4/6 | 2/6 | 2/7 | | | 1/6 |
| Good communication between provider-patient | **14** | 1/7 | 1/7 | 2/6 | | 2/7 | 1/7 | 3/6 | 4/6 |
| Shared decision-making provider-patient | **13** | 2/7 | | 4/6 | 1/6 | 1/7 | 1/7 | | 4/6 |
| Respectful interaction between provider-patient | **12** | 2/7 | 1/7 | 5/6 | | | | | 4/6 |
| Shared information between providers | **10** | 2/7 | 1/7 | 1/6 | 2/6 | 2/7 | | 1/6 | 1/6 |
| Team work between providers | **10** | | 1/7 | | 2/6 | | 2/7 | 2/6 | 3/6 |
| Confidence in knowledge and skills in providers | **10** | 1/7 | 2/7 | 3/6 | 2/6 | 1/7 | | 1/6 | |
| Pro-active, prevention-oriented, care | **13** | | 1/7 | 1/6 | 4/6 | 4/7 | | | 3/6 |

Note: 1 = Group split into 2, so each time # out of 6 persons, each group discussed either health/well-being and costs or care and costs.



For peer review only - http://bmjopen.bmj.com/site/about/guidelines.xhtml

<u>**Supplementary files**</u> accompanying the manuscript:

Defining good health and care from the perspective of persons with multi-morbidity: Results from focus groups in eight European countries

**Box S.1:** Extended protocol of the focus group

**Introduction**

- Purpose: to discuss what is important from the perspective of an individual with multiple health- and/or social problems in health/well-being and in care
- Overarching aim: the results of the discussion will be used to improve the care for persons with multiple health- and social problems
- Agenda explanation
- 'Rules'
  - No right / wrong answers, only differing points of view
  - Not necessary to agree with others, but must listen respectfully as others share their views
  - Talk to one-another, researchers only have role as moderator in guiding the discussion
  - Privacy / anonymity
  - The discussion will be recorded, and notes will be made
  - Turn off phones
- Informed consent
- Introduction of participants to one-another

**Part 1: Defining good health/well-being and care**

Step i):

- When you think about your <u>health and well-being</u>: try to complete this sentence / thought: "For me, being in great health means…"
- When you think about the <u>care</u> that you receive, have received, or may receive in the future, try to complete this sentence / thought: "I'd be really satisfied with all of the care / the overall care that I receive, if…"

**Write this question on a large white board / flip-over*

  - Provide examples if participants are having trouble understanding what you mean:
    - …being able to work full-time. (health)
    - …being able to spend time with family and friends. (health)
    - …being able to live for many more years. (health)
    - …not going to the doctor so often. (health)
    - …my doctor took more time to explain things to me. (care)
    - …my partner would always be allowed to join me and discuss with the doctors as well. (care)
    - …my care plan was discussed with me. (care)
    - …I had one overall written care plan. (care)
    - …I always knew I could contact one person in case of questions. (care)

**The researcher needs to ask triggering and thought-provoking questions here.*

**The discussion should be about health and well-being in general, not about care specifically (yet).*

**Be careful not to provide too many examples and to steer the answers in one direction.*

**The researcher leading the discussion will be writing these thoughts out on a white board / flip-over.*

For peer review only

**The researchers need to allow the participants to reflect on one-another's ideas.*
**Close the discussion with a final question: i.e., have we missed anything?*

Step ii):

- Once it seems as though all participants have said what is important to them, the researcher will go through the points on the board, and with the help of the participants try to extrapolate what these things mean, in order to go from **specific** examples to **general** outcomes**.** For example:
  - "For me, being in good health means being able to work full-time", does this mean:
    - Participating in society?
    - Being able to support one-self financially? → Self-sufficiency
    - Having social contacts?
    - Having a daily structure?
  - "For me, being in good health means not going to the doctor so often", does this mean:
    - Having no acute health problems?
    - Having the ability to deal with minor health problems on your own? (self-management)
  - "I'd be really satisfied with all of the care / the overall care that I receive, if my doctor took more time to explain things to me", does this mean:
    - Good communication?
    - Shared-decision making?
    - More/enough time?
  - "I'd be really satisfied with all of the care / the overall care that I receive, if my partner could always be allowed to join me and discuss with the doctors as well.", does this mean:
    - Understanding your personal situation?
    - Involvement of the informal caregiver?
    - Good communication?

**The researcher needs to write key words that come forth on large notecards and place them in the centre of the table for all participants to be able to see.*
**The researcher needs to ask triggering and thought-provoking questions here – what do the points mentioned in step 1 really mean? What does it come down to? What would it mean for the person sitting next to you? How can you expand this so that it 'counts' for everyone in the room? Try to distinguish between individual opinions and actual group consensus.*
**In asking further questions, try to focus on multi-morbidity – how is this specific to you as a person with multiple health- and/or social problems, would this be different if you had a single disease?*
**For each initial statement, multiple cards can be made.*
**Close the discussion with a final question: i.e., have we covered all elements?*

**Break**

**Place cards from part one onto the table, sort them by 'triple aim'*
**Sort through original 77 cards, and add cards to the table from these (refrain from overlap, choose card from part one in words of participants over literature-cards)*

For peer review only

**Part 2: Selecting most important concepts**

- Instructions: "In the discussion during part 1 you've come up with points that are important for you when it comes to defining health and care. As you saw, we've been trying to write this into more general terms on these cards."
- In our research team we've also been looking at concepts that are often mentioned in research/literature as being important, we've added these to the table as well."

**Explain the new concepts in case anything is unclear.*

- "We'd like to ask each of you to look at all of these cards and write down for yourself which 10 are most important to you. This can be a mix of health/well-being, care, and cost cards."
- "Now we'd like you all to discuss your top 10 and try to see whether you can reach a group consensus, and make a top 10 of cards at the centre of the table."

**Ask a participant to first put his/hers down or read his/her list first, through discussion see whether some kind of consensus can be reached.*

For peer review only

**Box S.2:** List of literature-based outcomes used on cards in part two

**Health / well-being**

- Biomedical outcomes: e.g., blood sugar, blood pressure, lung function, kidney function
- Life expectancy
- Maintaining independence
- Activities of daily living: e.g. eating, bathing
- Independence from medical aids: e.g., wheelchair, oxygen
- Energy and fatigue
- Pain and discomfort
- Disability
- Physical mobility: e.g., being able to walk
- Work ability: being physically and mentally able to work
- Cognitive functioning: e.g., thinking, memory, concentration and learning
- Anxiety and depression
- Stress
- Worrying
- Listless: no spirit, apathetic, indifferent
- Self-esteem: a good feeling of one-self, self-respect
- Respect from others
- Social relationships: staying in contact with family/friends/acquaintances
- Maintaining social status: in terms of 'societal class', 'occupation', how you think others look at you in society.
- Societal participation: e.g., volunteer work, club memberships, community activities
- Social support: emotional, instrumental and financial assistance from the social network
- Loneliness
- Investing in future health and wellbeing
- Self-efficacy: belief in one's capacity to follow a needed or desired course of action
- Positive frame of mind, resilience: ability to maintain a positive perspective regarding the future, despite setbacks or change
- Coping
- Being able to deal with problems, losses, adverse events in an effective manner

- Health literacy: capacity to obtain, process, and understand information about illness and health
- Compliance/adherence to treatment
- Lifestyle: e.g., smoking, nutrition, physical activity, alcohol and drug use
- Feeling safe
- Enjoyment of life
- Maintaining dignity
- Living comfortably
- Caregiver burden

**Experience with care**

- Holistic assessment
- Shared decision-making
- Tailored care
- Individualised care planning
- Pro-active, prevention-oriented care
- Clear responsibilities and accountability
- Named coordinator
- Team work and collaboration between professionals
- Good communication
- Shared information between professionals and providers
- Continuity of care: pertains to continuity (e.g., follow-up, monitoring, stability, transfer) throughout time and between professionals and organisations)
- Confidence in knowledge and skills in professionals
- Respectful interaction between provider-patient: feeling of being treated with respect and being ‘heard’
- Geographical access: e.g., facilities close to home
- Physical access: e.g., building is wheelchair accessible
- Timely access: e.g., no/short waiting lists
- Satisfaction with care process

**Costs**

- Service and support coverage: co-payments/deductible and financial access to services
- Travel and parking costs
- Loss of income – patient
- Loss of income - informal caregiver

For peer review only

**Box S.3:** Most important concepts in health/well-being, care, and costs per focus group participant per country (part 2 of the focus groups)

| | # / 58 | Austria | Croatia | Germany[1] | Hungary | Netherlands | Norway | Spain | UK |
|---|---|---|---|---|---|---|---|---|---|
| *Health and well-being* | | | | | | | | | |
| **Physical well-being** | | | | | | | | | |
| Energy & fatigue | **12** | 4/7 | | | 1/6 | 4/7 | 2/7 | | 1/6 |
| Pain & discomfort | **4** | 3/7 | | | | | | | 1/6 |
| Biomedical outcomes | **3** | 2/7 | | | 1/6 | | | | |
| Disability | **5** | | 2/7 | | 1/6 | | 1/7 | | 1/6 |
| Progression free health state* | **2** | | | | 2/6 | | | | |
| Physical mobility | **5** | | 1/7 | 1/6 | 1/6 | 1/7 | 2/7 | | |
| Activities of daily living | **7** | | 1/7 | 3/6 | 1/6 | 1/7 | | | 1/6 |
| Life expectancy | **1** | | | | | 1/7 | | | |
| To be active* | **2** | | | | | | 2/7 | | |
| **Feeling safe** | **10** | | 1/7 | | 3/6 | 3/7 | | 3/6 | |
| Discrimination* | **3** | | 3/7 | | | | | | |
| **Cognitive functioning** | **12** | 4/7 | | 1/6 | | 2/7 | 1/7 | 3/6 | 1/6 |
| **Independence** | | | | | | | | | |
| Being able to choose what you do / want to do* | **1** | | | | | | | 1/6 | |
| Moving freely around street * | **1** | | | | | | | 1/6 | |
| Independence from medical aids | **3** | | | | 1/6 | | | 1/6 | 1/6 |
| Maintaining independence | **16** | | 2/7 | 5/6 | 1/6 | 1/7 | | 1/6 | 1/6 |
| Living comfortably | **5** | 1/7 | | 2/6 | | 1/7 | | | 1/6 |
| **Psychological well-being*** | **3** | | | 3/6 | | | | | |
| Listlessness | **3** | 2/7 | | | | | | 1/6 | |
| Loneliness | **2** | 2/7 | | | | | | | |
| Empathy* | **1** | | 1/7 | | | | | | |
| Stress/worrying | **7** | | | | 3/6 | | 1/7 | | 3/6 |
| Anxiety & depression | **2** | | | | 1/6 | | | | 1/6 |
| **Enjoyment of life** | **16** | 2/7 | | 4/6 | 1/6 | 3/7 | 3/7 | | 3/6 |
| **Positive frame of mind, resilience** | **16** | 2/7 | 3/7 | 2/6 | 2/6 | 1/7 | 2/7 | 2/6 | 2/6 |
| Creativity* | **2** | | 2/7 | | | | | | |
| Investing in future | **1** | | | | | | | 1/6 | |
| Coping | **5** | 1/7 | | | | | 2/7 | 1/6 | 1/6 |
| Maintaining dignity | **9** | 1/7 | | | 4/6 | | | 2/6 | 2/6 |
| Self-esteem | **11** | 1/7 | 1/7 | 3/6 | | 3/7 | 2/7 | | 1/6 |
| Acceptance* | **4** | | | 3/6 | | 1/7 | | | |
| Look out for possibilities* | **1** | | | | | | 1/7 | | |
| Hope | **1** | | | | | | 1/7 | | |

For peer review only

| | # / 58 | Austria | Croatia | Germany[1] | Hungary | Netherlands | Norway | Spain | UK |
|---|---|---|---|---|---|---|---|---|---|
| **Social relationships & participation** | | | | | | | | | |
| Social relationships | **17** | 3/7 | | 5/6 | 2/6 | 4/7 | | 2/6 | 1/6 |
| Societal participation | **12** | 1/7 | 3/7 | 3/6 | | 3/7 | | 1/6 | 1/6 |
| Respect from others | **6** | | | | | 2/7 | 2/7 | 1/6 | 1/6 |
| To be taken seriously* | **3** | | | | | | 3/7 | | |
| Social support | **5** | 1/7 | 1/7 | | | | 2/7 | 1/6 | |
| Being able to relate to other people without physical limits* | **1** | | | | | | | 1/6 | |
| Feeling useful* | **2** | | | | | | | 2/6 | |
| Get something off one's chest* | **2** | 2/7 | | | | | | | |
| (Maintaining good) social status | **5** | 2/7 | | 3/6 | | | | | |
| Not being reduced to the disease* | **6** | 1/7 | | | | | | | |
| To be regarded as whole human being* | | | | | | | 4/7 | | |
| Seeing past the outside/surface of a person* | | | | | | | 1/7 | | |
| Surrounding area takes care of someone's needs* | **1** | 1/7 | | | | | | | |
| Treatments compatible with work* | **1** | 1/7 | | | | | | | |
| Work ability | **6** | | 3/7 | | 1/6 | 2/7 | | | |
| Social expectations* | **1** | | 1/7 | | | | | | |
| Daily structure* | **4** | | | 4/6 | | | | | |
| Being able to plan* | **2** | | | | | | 2/7 | | |
| Burden for informal caregivers* | **3** | | | 2/6 | | | 1/7 | | |
| **Self-management*** | **7** | | | 6/6 | | 1/7 | | | |
| Lifestyle | **3** | 2/7 | | | | | | | 1/6 |
| Compliance/adherence to treatment | **5** | 1/7 | | 2/6 | 1/6 | | | | 1/6 |
| Self-efficacy | **6** | | 2/7 | | 1/6 | | | 1/6 | 2/6 |
| Investing in future health & well-being | **4** | | 1/7 | 2/6 | | 1/7 | | | |
| Health literacy | **7** | | | 5/6 | 1/6 | 1/7 | | | |
| **Daily quality of life** | **1** | | | | | | 1/7 | | |
| *Experience* | | | | | | | | | |
| **Individualised care planning / tailored care** | **13** | 1/7 | | 4/6 | 1/6 | 2/7 | 4/7 | | 1/6 |
| **Holistic assessment/understanding** | **11** | 1/7 | 1/7 | 4/6 | 2/6 | 2/7 | | | 1/6 |
| **Good interactions providers-patients** | | | | | | | | | |
| Good communication | **14** | 1/7 | 1/7 | 2/6 | | 2/7 | 1/7 | 3/6 | 4/6 |
| Encourage patients* | **1** | | | | | | | 1/6 | |
| Psychological support* | **2** | | | 1/6 | | 1/7 | | | |
| Optimist reactions* | **1** | | | | | | | 1/6 | |
| Shared decision-making | **13** | 2/7 | | 4/6 | 1/6 | 1/7 | 1/7 | | 4/6 |
| Respectful interaction between provider-patient | **12** | 2/7 | 1/7 | 5/6 | | | | | 4/6 |
| Trust* | **7** | | | 3/6 | | 4/7 | | | |
| Predictability of care service delivery* (good info) | **1** | | | | 1/6 | | | | |
| Flow of information* | **2** | | | | | | 2/7 | | |

For peer review only

| | # / 58 | Austria | Croatia | Germany[1] | Hungary | Netherlands | Norway | Spain | UK |
|---|---|---|---|---|---|---|---|---|---|
| Being listened to and believed* | **2** | | | | | | | | 2/6 |
| **Good collaborations providers** | | | | | | | | | |
| Shared information between providers* | **10** | 2/7 | 1/7 | 1/6 | 2/6 | 2/7 | | 1/6 | 1/6 |
| Data transparency* | **3** | | | 3/6 | | | | | |
| Team work between providers | **10** | | 1/7 | | 2/6 | | 2/7 | 2/6 | 3/6 |
| Solidary distribution between funds* | **1** | 1/7 | | | | | | | |
| Clear responsibility and accountability | **5** | | 1/7 | 3/6 | | | | | 1/6 |
| Named coordinator | **7** | | 1/7 | 4/6 | | 1/7 | 1/7 | | |
| **Continuity of care** | **9** | | 2/7 | 2/6 | 3/6 | 1/7 | | 1/6 | |
| Collaboration across sectors* | **5** | | | 4/6 | | | | | 1/6 |
| Coordination between primary care and hospitals* | **1** | | | | | | | 1/6 | |
| Prevent and avoid going to ER* | **2** | | | | | | | 2/6 | |
| Administrative barriers* | **1** | | 1/7 | | | | | | |
| Transferring of responsibility* | **1** | | 1/7 | | | | | | |
| Transfer-care* | **2** | | | 1/6 | | 1/7 | | | |
| After-care* | **2** | | | 1/6 | | 1/7 | | | |
| Systematic operating procedures in care* | **3** | | | | 3/6 | | | | |
| Utilisation of EMRs* | **2** | | | 3/6 | 2/6 | 2/7 | | | |
| Careful use of patient files / privacy | **2** | | | 2/6 | | | | | |
| **Confidence in knowledge and skills in providers** | **10** | 1/7 | 2/7 | 3/6 | 2/6 | 1/7 | | 1/6 | |
| Education of health professionals* | **3** | | 3/7 | | | | | | |
| Importance and function of centres of excellence* | **1** | | 1/7 | | | | | | |
| Broader specialisations* | **1** | | | | | | 1/7 | | |
| **Access & availability** | | | | | | | | | |
| Physical access | **5** | | 1/7 | 1/6 | | 2/7 | | 1/6 | |
| Timely access* (i.e., short waiting times) | **9** | | 2/7 | 1/6 | 2/6 | 1/7 | | | 4/6 |
| Enough time* (provider for patient) | **8** | 4/7 | | | 1/6 | 1/7 | | 2/6 | |
| Being seen by specialist when needed* | **2** | | | | | | | 2/6 | |
| Geographical access | **3** | | 1/7 | | | | | 1/6 | 1/6 |
| Availability of treatment* | **3** | | 3/7 | | | | | | |
| Home care (available) | **1** | | | 1/6 | | | | | |
| Transport* | **1** | | | 1/6 | | | | | |
| **Avoid unnecessary treatment*** | **2** | 2/7 | | | | | | | |
| **Pro-active, prevention-oriented, care** | **13** | | 1/7 | 1/6 | 4/6 | 4/7 | | | 3/6 |
| **Patient-education and support (groups)** | | | | | | | | | |
| Integration of self-help groups* | **3** | 3/7 | | | | | | | |
| Importance of associations of patients* | **1** | | 1/7 | | | | | | |
| Good trainings offered* | **1** | 1/7 | | | | | | | |
| Medication help* | **5** | | | 1/6 | | 4/7 | | | |
| Self-management support* | **1** | | | | | 1/7 | | | |

For peer review only

| | # / 58 | Austria | Croatia | Germany[1] | Hungary | Netherlands | Norway | Spain | UK |
|---|---|---|---|---|---|---|---|---|---|
| Good info for patients on health | **1** | | | | | | 1/7 | | |
| Family support* | **3** | | 3/7 | | | | | | |
| **Bureaucratic hurdles*** | **5** | | | 4/6 | | 1/7 | | | |
| **Physical surroundings** | | | | | | | | | |
| Material conditions in institutions* | **1** | | 1/7 | | | | | | |
| **Macro-level** | | | | | | | | | |
| Non-cooperation with health policy makers* | **2** | | 2/7 | | | | | | |
| Importance of media pressure | **2** | | 2/7 | | | | | | |
| Learning from different countries' experiences* | **1** | 1/7 | | | | | | | |
| **Overall satisfaction with care process** | **8** | 1/7 | | 4/6 | 2/6 | | | 1/6 | |
| *Costs* | | | | | | | | | |
| **Loss of income** | | | | | | | | | |
| Loss of income informal caregiver | **3** | | 1/7 | | | | | 2/6 | |
| Loss of income patient | **7** | 1/7 | 3/7 | | 1/6 | | | 1/6 | 1/6 |
| Overall costs in relation to income* | **1** | | | | | 1/7 | | | |
| **Service and support coverage** | **6** | 2/7 | 1/7 | 1/6 | 1/6 | | 1/7 | | |
| **Need for cost-benefit analysis*** | **2** | | 2/7 | | | | | | |
| **Money follows the patient*** (bundled type payment that crosses all care level-borders) | **1** | | 1/7 | | | | | | |
| **Travel and parking costs** | **2** | | | | 1/6 | | | | 1/6 |

Note: 1 = Group split into 2, so each time # out of 6 persons, each group discussed either health/well-being and costs or care and costs. Btw = between, Profs = professional care providers, EMRs = Electronic Medical Records, ER = Emergency Room. * Concepts/phrases that were novel and written on cards based on part 1 of the focus groups, and not on the basis of the a priori literature search.

For peer review only

**Box S.4:** Criteria used to come to a core set of outcomes to evaluate integrated care programmes

- **Cover the Triple Aim**: 1) improving population health/well-being and 2) patient experience, and 3) reducing cost (growth);
- **Measurable at an individual level** using PROMS and PREMS (this only applies to the first and second of the three aims);
- **Relevant for multi-morbidity** in different contexts and population groups;
- **Relevant for the 5P stakeholders: p**atients (persons with multi-morbidity), **p**artners (informal caregivers), **p**rofessionals, **p**ayers, **p**olicy makers.
- **Relevant across the** 17 selected **promising integrated care programmes**;
- **Timing of intervention effects**: the intervention effect (i.e., the impact of a programme on the outcome) in newly enrolled patients is likely to start occurring within the SELFIE evaluation period (6-18 months);
- The **outcomes are not redundant**, meaning that they are all necessary and not superfluous (e.g., one outcome does not overlap much with another outcome);
- **Preference independence**, i.e., the weight of one outcome can be elicited independently from the performance of another outcome;
- **Feasible to measure** performance.

For peer review only

## COREQ (COnsolidated criteria for REporting Qualitative research) Checklist

A checklist of items that should be included in reports of qualitative research. You must report the page number in your manuscript where you consider each of the items listed in this checklist. If you have not included this information, either revise your manuscript accordingly before submitting or note N/A.

| Topic | Item No. | Guide Questions/Description | Reported on Page No. |
|---|---|---|---|
| **Domain 1: Research team and reflexivity** | | | |
| *Personal characteristics* | | | |
| Interviewer/facilitator | 1 | Which author/s conducted the interview or focus group? | 8 |
| Credentials | 2 | What were the researcher's credentials? E.g. PhD, MD | 8 (1) |
| Occupation | 3 | What was their occupation at the time of the study? | 8 (1) |
| Gender | 4 | Was the researcher male or female? | 11 |
| Experience and training | 5 | What experience or training did the researcher have? | 8 |
| *Relationship with participants* | | | |
| Relationship established | 6 | Was a relationship established prior to study commencement? | 7 |
| Participant knowledge of the interviewer | 7 | What did the participants know about the researcher? e.g. personal goals, reasons for doing the research | 8 |
| Interviewer characteristics | 8 | What characteristics were reported about the inter viewer/facilitator? e.g. Bias, assumptions, reasons and interests in the research topic | 8, 11 |
| **Domain 2: Study design** | | | |
| *Theoretical framework* | | | |
| Methodological orientation and Theory | 9 | What methodological orientation was stated to underpin the study? e.g. grounded theory, discourse analysis, ethnography, phenomenology, content analysis | 9 |
| *Participant selection* | | | |
| Sampling | 10 | How were participants selected? e.g. purposive, convenience, consecutive, snowball | 7 |
| Method of approach | 11 | How were participants approached? e.g. face-to-face, telephone, mail, email | 7 |
| Sample size | 12 | How many participants were in the study? | 11 |
| Non-participation | 13 | How many people refused to participate or dropped out? Reasons? | 11 |
| *Setting* | | | |
| Setting of data collection | 14 | Where was the data collected? e.g. home, clinic, workplace | 7 |
| Presence of non-participants | 15 | Was anyone else present besides the participants and researchers? | 7, 11 |
| Description of sample | 16 | What are the important characteristics of the sample? e.g. demographic data, date | 7, 11 |
| *Data collection* | | | |
| Interview guide | 17 | Were questions, prompts, guides provided by the authors? Was it pilot tested? | 7-8 |
| Repeat interviews | 18 | Were repeat inter views carried out? If yes, how many? | 7-8 |
| Audio/visual recording | 19 | Did the research use audio or visual recording to collect the data? | 8 |
| Field notes | 20 | Were field notes made during and/or after the inter view or focus group? | 8-10 |
| Duration | 21 | What was the duration of the inter views or focus group? | 11 |
| Data saturation | 22 | Was data saturation discussed? | 9 |
| Transcripts returned | 23 | Were transcripts returned to participants for comment and/or | 9-10 |

For peer review only

| Topic | Item No. | Guide Questions/Description | Reported on Page No. |
|---|---|---|---|
| | | correction? | |
| **Domain 3: analysis and findings** | | | |
| *Data analysis* | | | |
| Number of data coders | 24 | How many data coders coded the data? | 9 |
| Description of the coding tree | 25 | Did authors provide a description of the coding tree? | 9 |
| Derivation of themes | 26 | Were themes identified in advance or derived from the data? | 9 |
| Software | 27 | What software, if applicable, was used to manage the data? | 9 |
| Participant checking | 28 | Did participants provide feedback on the findings? | 9-10 |
| *Reporting* | | | |
| Quotations presented | 29 | Were participant quotations presented to illustrate the themes/findings? Was each quotation identified? e.g. participant number | 9-10 (results) |
| Data and findings consistent | 30 | Was there consistency between the data presented and the findings? | 9-10 |
| Clarity of major themes | 31 | Were major themes clearly presented in the findings? | 9-10 |
| Clarity of minor themes | 32 | Is there a description of diverse cases or discussion of minor themes? | 9-10 |

Developed from: Tong A, Sainsbury P, Craig J. Consolidated criteria for reporting qualitative research (COREQ): a 32-item checklist for interviews and focus groups. *International Journal for Quality in Health Care*. 2007. Volume 19, Number 6: pp. 349 – 357

**Once you have completed this checklist, please save a copy and upload it as part of your submission. DO NOT include this checklist as part of the main manuscript document. It must be uploaded as a separate file.**

For peer review only

BMJ Open

# Defining good health and care from the perspective of persons with multi-morbidity: Results from a qualitative study of focus groups in eight European countries

| | |
|---|---|
| Journal: | *BMJ Open* |
| Manuscript ID | bmjopen-2017-021072.R1 |
| Article Type: | Research |
| Date Submitted by the Author: | 05-Apr-2018 |
| Complete List of Authors: | Leijten, Fenna; Erasmus Universiteit Rotterdam, Erasmus School of Health Policy & Management<br>Hoedemakers, Maaike; Erasmus Universiteit Rotterdam, Erasmus School of Health Policy & Management<br>Struckmann, Verena; Berlin University of Technology, Department of Health Care Management<br>Kraus, Markus; Institute of Advanced Studies<br>Cheraghi-Sohi, Sudeh; University of Manchester, NIHR Greater Manchester Patient Safety Translational Research Centre<br>Zemplenyi, Antal; Syreon Research Institute; University of Pécs, Healthcare Financial Management Department<br>Ervik, Rune; Uni Research Rokkan Centre<br>Vallve, Claudia; Consorci Institut D'Investigacions Biomediques August Pi i Sunyer (IDIBAPS)<br>Huic, Mirjana; Agency for Quality and Accreditation in Health Care and Social Welfare<br>Czypionka, Thomas; Institute of Advanced Studies<br>Boland, Melinde; Erasmus Universiteit Rotterdam, Erasmus School of Health Policy & Management<br>Rutten-van Mölken, Maureen; Erasmus Universiteit Rotterdam, Erasmus School of Health Policy & Management; Institute for Medical Technology Assessment, Erasmus University |
| <b>Primary Subject Heading</b>: | Patient-centred medicine |
| Secondary Subject Heading: | General practice / Family practice, Health economics, Health services research, Public health, Qualitative research |
| Keywords: | PREVENTIVE MEDICINE, QUALITATIVE RESEARCH, person-centered, multi-morbidity, evaluation outcomes, integrated care |
| | |

SCHOLARONE™
Manuscripts

**Manuscript title:** Defining good health and care from the perspective of persons with multi-morbidity: Results from a qualitative study of focus groups in eight European countries


**Authors**

Fenna Leijten[a], Maaike Hoedemakers[a], Verena Struckmann[b], Markus Kraus[c], Sudeh Cheraghi-Sohi[d], Antal Zemplényi[e,f], Rune Ervik[g], Claudia Vallvé[h], Mirjana Huiĉ[i], Thomas Czypionka[c], Melinde Boland[a], Maureen Rutten-van Mölken[a,j]*, on behalf of the SELFIE consortium

**Affiliations**

a) Erasmus University Rotterdam, Erasmus School of Health Policy & Management, Rotterdam, the Netherlands
b) Berlin University of Technology, Department of Health Care Management, Germany
c) Institute of Advanced Studies, Vienna, Austria
d) University of Manchester, NIHR Greater Manchester Patient Safety Translational Research Centre, Manchester, United Kingdom
e) Syreon Research Institute, Budapest, Hungary
f) University of Pécs, Healthcare Financial Management Department, Pécs, Hungary
g) Uni Research Rokkan Centre, Bergen, Norway
h) Consorci Institut D'Investigacions Biomediques August Pi i Sunyer (IDIBAPS), Barcelona, Spain
i) Agency for Quality and Accreditation in Health Care and Social Welfare, Zagreb, Croatia
j) Institute for Medical Technology Assessment, Erasmus University Rotterdam, Rotterdam, the Netherlands

*Corresponding author: Maureen Rutten-van Mölken: m.rutten@bmg.eur.nl / Tel: +31 10 4088559


For peer review only



For peer review only - http://bmjopen.bmj.com/site/about/guidelines.xhtml

**Abstract**

**Objectives**: The prevalence of multi-morbidity is increasing in many Western countries. Persons with multi-morbidity often experience a lack of alignment in the care that multiple health- and social care organisations provide. As a response, integrated care programmes are appearing. It is a challenge to evaluate such programmes and to choose appropriate outcome measures. To gain insight into meaningful outcomes, focus groups were held with persons with multi-morbidity in eight European countries, in which participants defined 'good health and well-being' and a 'good care process'.

**Methods**: In 2016, eight focus groups were organised with persons with multi-morbidity in: Austria, Croatia, Germany, Hungary, the Netherlands, Norway, Spain, and the UK (total N=58). Each focus group followed the same two-part procedure: 1) defining a) good health and well-being, b) a good care process and 2) group discussion on prioritising the most important concepts derived from part one and from a list extracted from the literature. Inductive and deductive analyses were done.

**Results**: Overall, the participants in all focus groups concentrated more on the care process than on health. Persons with multi-morbidity defined good health as being able to conduct and plan normal daily activities, having meaningful social relationships, and accepting and coping with the current situation. Absence of shame, fear, and/or stigma, being able to enjoy life, and overall psychological well-being were also important facets of good health. Being approached holistically by care professionals was said to be vital to a good care process. Continuity of care and trusting professionals were also described as important. Across countries little variation in health definitions were found but variation in defining a good care process was seen.

**Conclusion**: A variety of health outcomes that entail well-being, social, and psychological facets and especially experience with care outcomes should be included when evaluating integrated care programmes for persons with multi-morbidity.



For peer review only - http://bmjopen.bmj.com/site/about/guidelines.xhtml

**Strengths and limitations of this study**

- Focus groups were held in 8 European countries amongst persons with multi-morbidity.
- The qualitative, focus group, approach applied in this study ensures that the perspectives of persons with multi-morbidity are incorporated in the discussion on what meaningful evaluation outcomes are.
- Only participants in one focus group (in the UK) explicitly named clinical psychological health problems as a co-morbidity, which may suggest that perspectives from persons with this type of health problem are under-represented in this study.
- Creating an overarching top-10 list of important outcomes in health and care is a summation of a qualitative process and thus this list should be interpreted cautiously.
- Although different countries and multi-morbidities were included, the applied qualitative method does not mean that the findings are necessarily transferable to all persons with multi-morbidity in all countries.

For peer review only



For peer review only - http://bmjopen.bmj.com/site/about/guidelines.xhtml

**Introduction**

With ageing populations in Western societies, there is an increasing prevalence of multi-morbidity, i.e., the co-occurrence of two or more health problems within one person at one time [1, 2]. Persons with multi-morbidity therefore often require care from different types of professionals. These professionals may work in different healthcare sectors (e.g., primary and secondary care) and may also work in social or community care. It is important that persons with multi-morbidity receive well-integrated care, in order to avoid the risk of fragmentation or overlap in the care received and interactions in treatment [3].

Integrated care is defined as structured efforts to provide coordinated, pro-active, person-centred, multidisciplinary care by two or more care professionals that effectively communicate and collaborate. There are different integrated care programmes for multi-morbidity being implemented across Europe that may offer a solution to the aforementioned risks that this population faces [4-7]. The evidence-base of such programmes is still limited and findings are not yet wholly convincing [5-7]. Increasing this evidence-base is important for the durability, wider implementation, and more sustainable reimbursement/financing of such programmes. Often, decisions on these matters are informed by economic evaluations in which costs per Quality Adjusted Life Years (QALYs) are calculated. However, it can be questioned whether the current (economic) evaluation framework provides sufficient insight into the broad range of outcomes that such integrated care programmes aim to improve. Integrated care programmes are complex interventions: they consist of various interacting components, target individuals but also groups and organisations, have a variety of intended outcomes, are amendable to tailoring through adaptation and learning feedback loops, and their effectiveness is impacted by the behaviour of those delivering and receiving the intervention [8]. Common generic outcomes such as QALYs may not fully capture what these programmes are actually trying to achieve in persons with multi-morbidity. Their aims may go beyond the improvement of life expectancy and health-related quality of life, and include the improvement of well-being, the maintenance of independence, and increasing satisfaction with the care process.

In a time when the scarcity of resources is evident and evidence-based decisions on the spending of these resources are warranted, it is crucial to set up appropriate evaluations that can be used to convince decision-makers. Outcomes in the evaluations of complex care programmes often correspond at the higher level to the so-called ‘Triple Aim’: improving population health [and well-being], improving the patient’s experience with care, and reducing cost(-growth) [9, 10]. These higher level outcomes, however, can be interpreted in different ways. Health can be defined as the absence of disease [11], or a wider perspective can be applied whereby health is seen as the complete physical, mental, and social well-being beyond merely the absence of disease [12]. More



For peer review only - http://bmjopen.bmj.com/site/about/guidelines.xhtml

recent definitions turn health into a more active term, as the ability to adapt [13] and as a 'meta-capability' that can be used to attain human value [14]. Similarly, experience with care can include many different aspects, such as the extent to which care is proactive or person-centred (e.g., two domains of the Patient Assessment of Chronic Illness Care (PACIC) instrument) [15]. Scientific literature found for a recent review also conducted in the context of the SELFIE research project, as the current study is (see Box 1), provided insight into which outcomes have been used in recent integrated care programme evaluations [5]. We saw that a wide array of indicators, corresponding to the Triple Aim, are being used. This is also reiterated by the findings in the review by Linton et al. (2016), whereby a total of 99 instruments to assess well-being were found, with a great variety between these [16].

Different perspectives and approaches can be taken when operationalising the Triple Aim and meaningful outcomes of integrated care programmes. In the current study we aim to better understand what good health or well-being and a good care process mean from the perspective of persons with multi-morbidity and to identify what they find most important in each. We hereby thus focus on two of the three 'Triple Aims'. We look at this from a cross-country perspective by conducting focus groups in eight European countries involved in the SELFIE research project on integrated care in multi-morbidity: the Netherlands, Austria, Croatia, Germany, Hungary, Norway, Spain, and the UK (see Box 1). Using a qualitative focus group approach encourages interaction between persons to take place that may allow for novel concepts and themes to arise.

<<<**Insert here Box 1:** Information on the SELFIE project>>>

For peer review only



For peer review only - http://bmjopen.bmj.com/site/about/guidelines.xhtml

**Methods**

Focus groups were chosen because we were interested in the perspectives of persons with multi-morbidity themselves when it comes to health/well-being and care. The qualitative focus group methodology allows for novel concepts to arise, and interaction between persons can strengthen this process. The Consolidated criteria for reporting qualitative research (COREQ) checklist was used to structure this manuscript [17]. Eight focus groups were conducted with persons with multi-morbidity, one in each SELFIE partner-country: the Netherlands (Rotterdam), Austria (Vienna), Croatia (Zagreb), Germany (Berlin), Hungary (Budapest), Norway (Bergen), Spain (Barcelona), and the UK (Manchester). These countries have a long history of universal health care systems that are either tax-funded or funded through insurance premiums. However, they each have a unique health- and social care provider system in which persons with multi-morbidity might face different challenges. For more information on the organisation of care in each context, see the macro level descriptions of each country in the 'thick description' reports available on the SELFIE website (www.selfie2020.eu). The Dutch SELFIE team provided all other partners with a protocol on how to conduct and report on the focus groups, held a teleconference with each partner to discuss the protocol, and provided additional support throughout the process. All focus groups were organised between spring and fall of 2016.

**Recruitment & patient and public involvement**

The goal was to recruit 6-8 persons for each focus group, with a mix of gender, age distribution, and types of multi-morbidity ($\geq$2 health and/or social problems) being desired. Different recruitment strategies were applied: participants were recruited via patient organisations (AT, DE, HR, NL, NO), medical professional organisations (HR), self-help groups (AT, DE), medical centres (AT, ES, HU), non-profit care organisations (e.g., Red Cross) (AT), and patient and public involvement groups (UK). We specified that we were searching for persons with multiple health- and/or social problems. Participants were also recruited via SELFIE national Stakeholder Advisory Boards (SAB) (AT, DE, HU, NL, NO). Each country has a SAB that reflects on SELFIE findings and consists of five stakeholder groups (5P's): Patients (persons with multi-morbidity), Partners (informal caregivers), Professionals, Payers, and Policy makers. At the time that the focus groups were being organised, a SAB meeting had just taken place in each country, with two to four persons with multi-morbidity present. These persons were reached out to for the focus groups and we tried to snowball via their networks, e.g., also via patient organisations.

Participants were reimbursed for their travel costs and in some countries a gratuity was made available either as a token of appreciation or to aid recruitment (AT, DE, NL). As in most cases



For peer review only - http://bmjopen.bmj.com/site/about/guidelines.xhtml

no verbatim transcripts were made, these were not returned to participants. However, in some cases, participants were emailed after the meeting to thank them for their participation (DE, ES, HU, NL, NO, UK) and also in some cases were sent notes (NL). About one year after the focus groups, all participants were sent an update on the overall focus group results across countries. The current publication is also to be shared with participants.

**Procedure**

An extensive protocol was developed (see Supplementary file Box S.1) that was followed in each focus group. The first focus group was held by the Dutch team, who added 'lessons learned' to the protocol to aid the subsequent focus groups. Each country made a protocol in their own language to use during the actual focus group, which was held in their respective language. The focus groups were all held in a meeting room in an office, university, or clinical setting, with two to four researchers from the respective country present, one being the chair and at least one taking extensive notes throughout the meeting. At least one of these researchers is a co-author on this publication (FL, VS, MK, SCS, AZ, RE, CV, MiH, MB, MRM). The focus groups were also recorded. These researchers had a minimum of a bachelor's degree and experience/training in patient-contact and/or qualitative research.

The focus groups consisted of an introduction and two main parts: 1) defining a) good health and well-being, and b) a good care process, and 2) discussion on most important concepts and creating 'top-10' lists. The planned duration of the focus groups was two hours.

At the onset, the researchers welcomed everyone and introduced themselves, as only some researchers present had already been in touch with participants via telephone or email beforehand. The researchers stated their names, current position, and background expertise/occupation. Following this, in the introduction the researchers stated the purpose of the focus group: to discuss what is important from the perspective of an individual with multiple health- and/or social problems when it comes to health and care. The agenda of the focus group (introduction, part 1, part 2) was described. Next, the 'rules' of the focus group were introduced (see Supplementary File Box S.1) (e.g., respectful interaction, phones off). Participants signed the informed consent form (see Ethics statement below). The recorder was then turned on and participants were asked to introduce themselves and briefly describe their multi-morbidities.

In part one of the focus group, participants were asked to define good health/well-being and a good care process. First, a discussion about good health/well-being was held, followed by a discussion about a good care process. We started by asking participants to complete the sentence "For me, being in great health means…" and "I'd be really satisfied with all of the care / the overall



For peer review only - http://bmjopen.bmj.com/site/about/guidelines.xhtml

care that I receive, if…" Answers were discussed and written on flip-over boards. During this discussion a researcher asked triggering questions when needed and tried to focus the discussion on either health/well-being or care. After this, a researcher went through the statements on the flip-over board and the group tried to move from specific examples to general outcomes. These more general outcomes were highlighted / marked on the flip-overs and written on cards. Again a researcher needed to ask thought provoking questions: What do these original statements really boil down to? How can you generalise this so that it is applicable for others in the room? Throughout both steps a researcher also could pose the question as to how this is especially relevant or different for multi-morbidity.

After part one, there was a 15-30 minute break. During the break, the researchers arranged the cards with concepts, or outcomes, mentioned during part one (e.g., on tables or white/magnet boards). They also sorted through the a priori made cards from the literature and added those *not* mentioned during part one to the display. These cards were made a priori by the Dutch team, and distributed to each organiser. The outcomes on the a priori made cards stemmed from publications on integrated care programmes for multi-morbidity that were included in a large scientific literature review that was also conducted in the SELFIE project [5]. All outcomes included in those integrated care evaluation publications were ordered according to the Triple Aim and, where possible, overlap was removed. This resulted in a list of 77 potentially relevant outcomes (51 health/well-being, 22 experience, 4 cost) (see Supplementary File Box S.2). Each focus group organiser had been asked to translate these outcomes to their respective languages and write them on cards, using different colours for each Triple Aim. The purpose of including also these outcomes in the second part of the focus groups was to provide participants with a large and 'complete' (on the basis of the available literature) overview of possible outcomes.

In part two, the researchers briefly explained to the group that the cards from part one were now on display, and that they also added novel cards on the basis of findings in the literature. These new concepts were mentioned one-by-one and, where unclear, explained. Participants were asked to look at all the concepts. They were asked to write down the ten concepts that were most important to them on a sheet of paper. Hereafter, a discussion was opened as to what was on everyone's 'top-10' list.

**Analyses**

All focus groups were recorded and extensive notes were made, pictures of flip-overs and cards were made, and the top-10 lists were collected. In two cases, verbatim transcriptions were also made (HR, NO). Reports were made in English by each SELFIE partner on their respective focus



For peer review only - http://bmjopen.bmj.com/site/about/guidelines.xhtml

group, following a predefined template: structure of the focus group, recruitment, participant characteristics, reflection on the process, findings part one (concepts reflecting good health/well-being and a good care process), findings part two (summation of top-10 lists), and conclusion/discussion.

Three analytical steps can be distinguished. First, throughout the reporting and summarising done for each focus group, the researchers present at the focus group reflected on their respective focus group; at least one of these researchers is a co-author. Next, these reports were analysed independently by the first, second, and second-to-last author from the Dutch team. During this analysis step themes and priorities, corresponding to part one and two, were extracted from each focus group and these were compared across focus groups. Third, these themes and priorities were discussed amongst all co-authors, thus including at least one of author of each initial report who was present at the focus group. The analysis of part one was done applying an inductive (bottom-up) approach, whereby different specific concepts mentioned by participants were analysed by describing these, looking at their overlap and differences, and when possible clustering these into more general concepts. By deciding upfront that the procedure and our findings would be structured according to the Triple Aim, we also applied a deductive (top-down) approach. For part two, further clustering was done to present the findings in a clearer way, and a summation was used to identify concepts that were found most important. No specific qualitative analysis software was used in analysing the data. The goal from the outset was to conduct one focus group per country, thus data saturation was not discussed a priori.

**Ethics statement**

This study was conducted in line with the Declaration of Helsinki. According to the laws of most participating countries (Austria, Croatia, Germany, Hungary, the Netherlands, Norway) this type of study is exempt from formal ethical approval by a Medical Ethics Committee. In line with their national laws two countries (Spain and UK) obtained ethical approval. This is not a study in which participants were allocated to treatment groups or underwent any treatment; participants were not recruited as 'patients' but merely as persons living in the community with multi-morbidity. Participation was completely voluntary. All recordings, transcripts, and analyses were treated anonymously. All focus group participants signed an informed consent form; they received this form prior to their attendance to the focus group and at the start of the focus group had time to ask questions and sign the form. This form was developed by the Dutch team on the basis of the WHO template for informed consent for qualitative research [18] and translated by each country into their respective language. The informed consent consisted of information on the study, the purpose of



For peer review only - http://bmjopen.bmj.com/site/about/guidelines.xhtml

254 the research, type of research, participant selection, voluntary participation, procedure, duration, potential risks, benefits, reimbursements, confidentiality, sharing of the results, right to refuse / withdraw, and contact information.

For peer review only



For peer review only - http://bmjopen.bmj.com/site/about/guidelines.xhtml

## Results

### Participant and focus group characteristics

In total 58 persons participated across the eight focus groups. Only in some cases specific persons did not participate, this was because we could not get in touch with these persons, they had a conflicting agenda/schedule (e.g., holiday), were too busy, or in some cases had to cancel last minute due to illness or last minute (healthcare) appointments.

In all focus groups six or seven persons participated, except in Germany where 12 persons participated. During part two of the focus group in Germany, two subgroups were created to ease discussion. The mean age per focus group is presented in Table 1 below. The overall mean age was 65 years (range 31-86).

During the focus groups most frequently three researchers (min 2, max 4) were present, whereby one led [part of] the discussion and one took notes (total researchers present = 20, M/F = 4/16). The focus groups took 2.5 hours on average (min 2, max 3).

The majority of persons named three morbidities during the introduction of the focus groups (Table 1). Health problems were categorised according to the WHO 2010 International Classification of Diseases (ICD-10) [19]. For an overview of the specific morbidities mentioned by each participant at the start of the focus groups see Table 2. Across countries, diseases of the circulatory system, musculoskeletal system, and endocrine disorders were most common morbidities amongst participants (Table 2). Specific examples of frequently mentioned diseases were high blood pressure, rheumatism, arthritis, and diabetes mellitus. It is of note that only in the UK mental health problems were explicitly named at the start of the focus groups (Table 2).

<<<**Insert here Table 1:** Focus group participant characteristics>>>

<<<**Insert here Table 2:** Morbidities of participants per country categorised according to the ICD-10>>

### Defining health and care (part 1)

*Defining health*

Across all focus groups the idea of good health being the ability to do **'normal' daily activities** was mentioned. This for example included activities such as going outside and undertaking activities (ES), being able to use a computer (UK), and doing physical activities within realistic reach:



For peer review only - http://bmjopen.bmj.com/site/about/guidelines.xhtml

"…Also being active is necessary for well-being. I was always very athletic nowadays only limited, but it works if you adapt your activities to your physical ability." (P2; AT)

*Response*: "That's true, though I cannot climb mountains anymore, but I can walk through different parks and I can still use the stairways." (P7; AT)

In three focus groups it was also said that especially in the case of multi-morbidity it is not only about doing such daily activities, but being able **to plan** them and **structure** them yourself (DE, NO, UK). This is related to having realistic expectations about what one can do and relates to acceptance. **Acceptance** was mentioned in terms of self-acceptance and acceptance by others (AT, DE, HU, NL). For example:

"Part of it is acceptance, I can do what I can do, and I should leave the other things." (P4; NL)

"How to handle the pain and the disease plays an important role. Integration of your diseases in your daily activities, accepting the pain and especially not feeling bad all the time although the diseases are permanent is a big step." (P11; DE)

"For me, I even feel good, if no additional things come. In my case, all of my conditions and problems that I was dealing with are considered as end-stage or final stage, and this is accepted. Therefore if my condition is not worsening, then I am fine." (P5, HU)

Daily activities are also linked to the desire to maintain **social relationships** and **participation** in society (AT, DE, ES, HR, HU, NL, NO).

"I want to be consciously active in society. With everything, doing what I want to do, being useful for others." (P7; NL)

"For me, good health means being psychosocially active…" (P1; HR) (i.e., having social relationships that are meaningful, being an active participant in everyday life)

In many countries good health was also associated with the **absence of shame, discrimination, fear, and/or stigma** (ES, HR, HU, NL, NO, UK). These feelings could relate to the wider public, the patient-provider, or the personal realm. For example, in Spain the examples of fear of walking alone and



For peer review only - http://bmjopen.bmj.com/site/about/guidelines.xhtml

being vulnerable or shame of being seen with medical apparatuses such as an oxygen machine were mentioned. In Hungary shame was mentioned in the patient-provider relation, e.g., not being able to keep to a diet. In Croatia these concepts were summarised as personal vulnerability. Fear of the future and not knowing how the disease trajectory will go was also discussed (HU, NL, NO). Doubts and worries about sharing updates on one's diagnoses with family and the impact and burden that might have was mentioned (AT). In the Spanish focus group the discussion on fear and shame was heated, as persons dealt and coped with this issue differently. This relates to a point that also came up, of not only accepting the health problems, but also **coping**, being resilient, managing, and having responsibility for the diseases themselves, which may be perceived to be especially difficult in the case of multi-morbidity (DE, HU, NL, NO, UK).

> "Oh yeah, I think if you have multiple [diseases] it just adds to the whole workload really, how to cope with different things, oh yeah." (P1; UK)

The general idea of good health being defined as **feeling safe** also was discussed in various focus groups (ES, HU, NL, NO, UK). This can be seen as the result of being absent from the aforementioned negative states (e.g., fear). Feeling safe was also discussed in terms of trusting professionals (discussed more below). In Norway, feeling safe also extended to the economic realm:

> "…fear, fear for one day having to give up your work for example … if I don't manage any more, if I am unable to work anymore, then we do not have the economic resources to live here anymore, this was in my thoughts when the illness hits me and then fear…" (P2; NO).

Lastly, during the open discussion in part one, participants mentioned having a positive frame of mind, being able to enjoy life, and the importance of **psychological well-being** was mentioned in all focus groups.

> "I think psychological problems ought to be mentioned. Many people with chronic illnesses are also struggling psychologically. It could be because you have bad conscience, because you are dreading something, because you do not know if you will manage and suffer from performance anxiety". (P1; NO)

> "I want to enjoy my life, even though I've these diseases. That means, just being full of life." (P4; AT)



For peer review only - http://bmjopen.bmj.com/site/about/guidelines.xhtml

*Defining care*

When considering the wide array of themes discussed when defining health/well-being, it is not surprising that good care was defined by many persons as being approached as a whole person and **being treated holistically** (AT, NO, UK).

> "I wish that people treat me in a respectful manner, because it's true, I am sick, but the disease is not me. I don't want to be reduced to my diseases." (P2; AT)

> "So I would have wished for a doctor that, to put it this way, had the overview of the whole human being, that he shouldn't treat a heart disease just in isolation, you have another disease, and a third..." (P2; NO)

Persons explained that being approached holistically also means receiving **holistic support**, including informal caregiver support (HR, NL, NO, UK), good information provision, and especially **emotional and psychological support.** Participants mentioned that support should take the form of more extensive, easily accessible, and timely information on the health problems at hand and medication, but also psychological support, support with self-management, and self-help groups for example (AT, DE, ES, HR, HU, NL). Concerning emotional and psychological support specifically, this is needed even when this is not the 'main' problem at hand (AT, ES, HR, NL, UK). For example, in the Netherlands participants mentioned that in the case of multi-morbidity it takes time to accept the new health problems that arise on top of existing health problems and that there should be support for this adjustment period. In the UK a disconnection between the patient- and provider's priorities was seen, as it was mentioned that the healthcare professionals do not focus on the mental health issues, when they should:

> "Yeah, for me I'd like the mental health to be bigger…for me all my healthcare professionals see the [physical disease] as the big thing with me, I don't, I see my depression as the big thing because that's what affects me day to day." (P4; UK)

> "What I see is that there's no psychological aid. When you are told you are a chronic patient and you have to take a drug all your life, and that this is for all your life, some people are depressed, needs psychological aid…" (P1; ES)



For peer review only - http://bmjopen.bmj.com/site/about/guidelines.xhtml

A wider theme discussed was **trust in professionals and the system**. This is in part linked to the emotional and psychological support, whereby participants felt that two-way trust is needed in the relationship between the patient and professional so that psychological issues can be discussed, the professional really listens, and so that they can embark on the care process together (AT, NL, NO, UK). Related to this, respectful interaction between professional and patient was also often mentioned (AT, DE, NL, NO, UK, HU). Specifically in the UK, the direction of trust was mentioned with participants saying that they needed to be 'believed' by the professionals (P2, P4, UK).

Trust also pertained to being able to rely on the professional in being able to help, based on their skills and knowledge (DE, ES, HR, NL, NO, UK). Participants spoke of having a 'prepared' care professional to talk to, and trusting their expertise and education.

> "Yeah, but there are difficulties with medication when you've got multiple things..." (P5; UK)
> *Response*: "Oh yeah, but you'd expect your GPs to keep on top of that, it is debatable as to whether that actually happens or not." (P1; UK)

In Hungary, Germany, and the Netherlands the trust also pertained to the professionals in general:

> "Trust in the medical world, well I'm sceptical about that, a lot of things happen around you, that you think, did they not see that?... Yeah, then you lose faith/trust because of the things that happen to you…" (P2; NL)

> "If I visit my doctor, I wish I could reproduce what he is doing there, which services are provided and I would like to sign for them. I want to be more informed about what is done. This way, I often cannot trust my physicians." (P8; DE)

As may be especially relevant in multi-morbidity, issues surrounding **continuity of care** were mentioned in all focus groups. This pertained to clear responsibilities, a clear contact point, transfer and 'after care', good communication, and good collaboration and teamwork. These points about continuity often also related to **sharing information or medical records** between providers and organisations (DE, ES, HR, HU, NO, NL).

> "The problem is the coordination between the primary health centres with the reference hospitals. The doctor at the hospital should see all the information, and the family doctor as well…" (P5; ES)



For peer review only - http://bmjopen.bmj.com/site/about/guidelines.xhtml

"I would expect that all the institutions and all the GPs in this city would be connected to the same electronical [IT] system. In this case I would not need to go with all those stacks of papers when I see a doctor. (...) There should be a system which can be seen by everyone and not only by some particular segments of the care." (P1, HU)

**Cross-country comparisons in defining good health and care (part 1)**

Little variation was seen between countries when defining health (part 1). Themes surrounding the definition of a good care process differed more between countries. Although aspects of access and availability were mentioned in all countries, this concept took a different form between the countries. In some cases this pertained to timely care in terms of waiting lists and enough time (ES, HU, NL). In Hungary, however, this specifically had to do with the lack of information on the waiting times. Persons emphasized that waiting times were more acceptable if they knew in advance how long they had to wait.

"The worst thing is waiting… They could calculate an order with some gap in between. There are problems with the information sharing" (P6; HU)

Access in the form of time also referred to professionals having enough time for persons with multi-morbidity in Germany, Hungary, and Norway.

Availability also had different definitions per country – in Croatia, Germany, and the UK this was treatment, care, and provider availability and the freedom to choose between them. In Norway, Spain, and the UK this was also geographical- availability and access.

Several concepts were only brought up in part one by certain countries. Namely, bureaucracy or the reduction of the burden thereof was mentioned in Germany and the Netherlands, for example with regard to care services that fall within our outside of the insurance package.

"There are persons who are physically not able to walk to their physiotherapist anymore. In fact they are in need of transport, but you have to apply for transportation for every single therapeutic unit. These are very difficult circumstances due to too much bureaucracy. This is disastrous…" (P9; DE)

In Austria participants discussed the need for more self-help and support groups. In contrast, in the Netherlands this was mentioned as a positive aspect already present and available. Participants in



For peer review only - http://bmjopen.bmj.com/site/about/guidelines.xhtml

Austria were aware that these things exist in other countries, and felt it was missing in their own context.

Lastly, physical surroundings in care provision were mentioned in Hungary that were related to cleanliness, enough personal space, and enough seats.

> "Because if we consider our life or our homes, we do not like if it is dirty and messy. In a good facility there should be cleanliness, order and discipline." (P2; HU)

> "At the … [department] patients need to wait in a very narrow corridor with one row of chairs. There are about three times more patients than chairs. (P5; HU)

Furthermore, in Hungary systematically organised operating procedures in care was an important requirement mentioned by multiple participants. The lack of organised procedures is exemplified by the fact that the care process in hospitals is sometimes unreasonably long, the staff is rushed, there are no clear instructions on the next steps of the procedure, there are redundant diagnostic tests, and information is not shared appropriately among professionals.

**Most important concepts (part 2)**

In all focus groups, in part 2, participants were asked to make a top-10 list of most important concepts. These concepts were those identified in part 1 and supplemented with 'missing' concepts from the literature (see Supplementary File Box S.2). These top-10 lists were discussed. There was large variation within focus groups (i.e., countries) as to what concepts were deemed most important and on participants' top-10 lists. In Supplementary File Box S.3 all concepts written on persons' top-10 lists are presented, including those from the literature and 'novel' concepts derived during part 1 of the focus groups.

In Table 3 below an overview of the most frequently mentioned concepts is presented. These are concepts written on the top-10 lists of at least 10 persons (out of the 58 participants in total) across all countries. The following health and well-being concepts were most frequently noted by participants on their top-10 lists: social relationships, a positive frame of mind or resilience, enjoyment of life, and maintaining independence. A positive frame of mind or resilience, was mentioned by at least one person in each country's focus group. Many facets pertaining to good interactions between care professionals and persons with multi-morbidity were on the top-10 lists of participants, such as good communication, shared decision-making, and respect. Furthermore, individualised care planning and a holistic assessment and understanding of the problems at hand



For peer review only - http://bmjopen.bmj.com/site/about/guidelines.xhtml

were aspects of the care process also frequently on the top-10 lists. Lastly, pro-active, prevention-oriented, care was found important by many participants. As can be seen, there is great variety in concepts that participants put on their top-10 lists, both within and across countries. Furthermore, some phrases and words are quite specific whereas others are broad; participants were free to determine at what conceptual level they wrote their top-10 outcomes.

**<<<Insert here Table 3:** Most frequently listed outcomes on the ‘top-10’ lists of focus group participants across the eight focus groups>>>

For peer review only



For peer review only - http://bmjopen.bmj.com/site/about/guidelines.xhtml

## Discussion

### Main findings

Participants defined good health and well-being in terms of being able to conduct 'normal' daily activities, being able to plan and structure these, and having social relationships and participating in society. Acceptance by oneself and by others and coping with one's current health situation were deemed aspects of good health. Absence of shame, discrimination, fear, and/or stigma from the public, care professionals and oneself, and on the other end feeling safe and psychological well-being were also facets of good health. Social relationships, resilience, enjoyment of life, and maintaining independence were considered the *most* important aspects of good health across participants from all countries. A good care process was defined as one whereby persons are approached and supported holistically, with specific attention for emotional and psychological support, there is confidence and trust in professionals and the system, continuity of care is guaranteed, and where information is shared and accessible within a reasonable time. Concepts deemed *most* important were good communication, shared decision-making, and respect between care professional and the person with multi-morbidity, as well as individualised care planning and pro-active, prevention-oriented, care. Little cross-country variation in health themes were found, however, in defining care differences did exist, for example in terms of the exact type of access referred to (e.g., geographical distance to care providers, timely access).

### Interpreting findings

Several themes brought up during the focus groups were explicitly mentioned to be more relevant for persons with multi-morbidity; this was especially the case when defining care. One such concept was enough time: in Norway persons mentioned that it is difficult when their issues are not immediately visible for a care professional and that they need time to explain the multiple problems at hand. Also confidence in professionals' skills are related to their needing to address or at least be aware of multiple problems and often multiple medications, some of which may go beyond their specialist area of expertise. Considering the different combinations of morbidities represented in the focus groups and the fact that participants are living in different health- and social care contexts, it is interesting to note that regardless of whether the main care professional came from primary, secondary, or social care, these aspects relating to respect, providing emotional support, and expertise were important for many participants. This might be central to the fact that persons with multi-morbidity deal with multiple care professionals who need to have attention for the person as a whole, considering all their morbidities and the emotional burden that comes alongside these. In line with this, well-coordinated and smooth transitions (i.e., continuity of care) are particularly



For peer review only - http://bmjopen.bmj.com/site/about/guidelines.xhtml

important for persons with multi-morbidity, who often cross provider-, organisation- and sector boundaries throughout their care trajectories. In the realm of continuity of care, in many focus groups sharing information for example via shared electronic medical records was explicitly mentioned. It is of note that issues surrounding privacy were not mentioned by participants, although EMRs are in most cases not in place yet due to such issues at a wider system level.

Aspects especially to do with defining care seemed relatively unique and extra relevant for multi-morbidity. However, there was a large degree of overlap in the concepts mentioned in defining health in the current focus groups and the existing definitions presented in the introduction by the WHO, Huber and Vankatapuram, and similar studies conducted on defining health [12-14]. Namely, absence of shame, discrimination, fear, and/or stigma, feeling safe, psychological well-being, and social relationships and participation especially overlap with the mental and social well-being aspects of the 1946 WHO definition of health [12]. The importance of mental health outcomes has also been recently found in a study amongst a UK general population on what aspects of the EuroQol 5-dimensions (EQ-5D) are currently missing or receive too little attention, i.e., mental health [20]. This is also in line with a study by Ebrahimi and colleagues (2012) amongst frail elderly where being able to 'master' daily life (e.g., coping and acceptance) and being happy and satisfied with life (e.g., enjoyment of life) were described as characteristics of health [21].

Other concepts found in the current focus groups relate more to the definition by Huber and colleagues of health as the ability to adapt, i.e., acceptance, coping, resilience [13]. Also overlap was seen with our finding on the ability to conduct normal daily activities and the aforementioned study on frail elderly where the ability to live the routine life persons are accustomed to was identified as an aspect of health [21]. Lastly, especially Venkatapuram's definition of health as the capability to attain human value, seems to overlap with the general idea of the ability to participate in normal daily activities and social participation (a means to attain human value) and enjoyment of life as mentioned throughout the current focus groups [14].

The facets of good health found in the current study for a large part overlap with facets of functioning as set out by the International Classification of Functioning (ICF) [22]. The goal of the ICF is to describe a person's functioning, irrespective of their specific health conditions, considering their context. The ICF can be used to classify aspects of functioning that can be measured when assessing the impact of, for example, integrated care programmes. However, in the current study we aimed to identify aspects of 1) health and wellbeing, and 2) experience with care, that can be used when evaluating the impact of integrated care programmes on the Triple Aim. Our findings are reaffirmed by the fact that the different themes and concepts that persons in the focus groups used to define good health can be retraced to the ICF.


For peer review only - http://bmjopen.bmj.com/site/about/guidelines.xhtml

It was noticeable that, across all focus groups, aspects relating to care were already mentioned in defining good health. It appeared that persons could not always clearly distinguish the two. This might be due to the fact that persons more easily can identify these care-related factors as 'changeable'. When exploring which aspects of health/well-being and care were most important in part two of the focus groups, we saw across countries that these overlapped with those mentioned during the first part of the discussions but also that often concepts were included from the literature-cards. As these were introduced and explained after the break, participants reacted positively to them. This indicates conceptual overlap in what is currently being assessed in evaluations, and thus found in the literature a priori, and what participants of our focus groups found important [5].

**Strengths & limitations**

Several limitations in the current study should be mentioned. Only in the UK mental health problems were explicitly mentioned by participants when they listed their morbidities at the start of the focus groups. The difference in explicitly naming mental health problems between countries may relate to culturally-related stigma issues. For example, throughout the Dutch focus group it became apparent that depression issues were also present amongst participants. Also in the German focus groups mental health problems, such as depression, were mentioned as 'side effects' of other health problems. It is possible that our findings would have differed had more persons with (diagnosed) psychological multi-morbidities been present at the focus groups. It could be that different aspects related to health and care would have been pointed out, for example because in many health- and social care systems of the included eight countries there is fragmentation between psychological- and somatic health care provisions. It is possible that certain findings would have been strengthened, such as the importance of being approached holistically and that somatic care professionals pay attention to psychological health problems and provide emotional support. Although having a more varied group of participants in this sense would be desirable, it is not a requirement nor a goal in qualitative studies to attain a representative sample.

A strength of the current study is that inductively and deductively derived outcomes were used in the second part of the focus groups. We chose to do so in order to provide participants with a broad set of possible outcomes to choose from. The added outcomes from the literature were, alongside novel concepts from part one of the focus groups, on participants' 'top-10' lists (see Supplementary File Box S.1). This indicates that simply because an outcome was not mentioned inductively in part one of the focus group, this did not mean that participants did not find it important.



For peer review only - http://bmjopen.bmj.com/site/about/guidelines.xhtml

For peer review only

Initially, the goal of the second part of the focus groups was to reach a group 'top-10', in which participants would discuss their individual lists and try to create a group list. However, due to time restrictions this was not possible. Thus the results of part two reflect each individual's opinion. We opted to present findings in the results section mentioned by ten or more persons across the focus groups, but this is a relatively arbitrary cut-off point, and the full findings are shown in the Supplementary File Box S.3. The total number of cards from which participants could select concepts for their top-10 list differed in size depending on from which country they are from. The level of specificity in concepts also differs greatly, for example the overall satisfaction with care process is mentioned, as well as the specific facet that a provider has enough time to listen to a patient (see Supplementary File Box S.3). Quantifying such a qualitative process is thus to be done cautiously. In the Supplementary File Box S.3 concepts are clustered into broader categories. There are, however, different categorisations possible, since the concepts are all interrelated and different definitions can be applied.

We consider it a strength of the current research that the participating countries in SELFIE differ in geographical size, population [density], culture, and welfare and health care systems – providing a wide scope. Furthermore, it is a main strength that persons with multi-morbidity were so actively approached and included in the current study and in the wider SELFIE study, introduced in Box 1. As will be described below, the results of these focus groups form the predominant input for the core set of outcomes to be included in the SELFIE evaluations of 17 integrated care programmes: participants were given a direct voice and greatly valued this. Another main strength of the current study and great addition to the literature is the fact that we asked participants in our focus groups to not only reflect on health, but also on experience with care. In situations whereby health improvements, especially in the traditional physical and clinical health indicators, are not feasible, focusing on satisfactory and even positive care process experiences becomes more important. And, as was seen in the current study, experience with care is often inextricably linked to the definition of health. Although we did not explicitly incorporate costs in the first part of the focus groups when discussing what outcomes are important, participants did mention several cost concepts such as commuting costs and out-of-pocket fees. In the future, it would be interesting to also have an explicit discussion on what role costs play in, especially, the choice of and experiences with care.

**Next steps**

When evaluating integrated care programmes for multi-morbidity, a wide array of health- and care outcomes should be included. Few aspects related to physical functioning, a traditionally used outcome, were found on the top-10 lists of importance, i.e., only 'energy and fatigue' was



For peer review only - http://bmjopen.bmj.com/site/about/guidelines.xhtml

mentioned by more than 10 persons across all focus groups. Instead, more well-being, social, and psychological facets of health are important and should thus be included in evaluations. This finding overlaps with current trends in conducting more comprehensive evaluations. To this end, in SELFIE a core set of outcomes for the evaluation of 17 promising integrated care programmes for multi-morbidity was developed that places a lot of focus on such outcomes. The core set consists of the following outcomes: physical functioning, psychological well-being, social relationships & participation, enjoyment of life, resilience, person-centeredness, continuity of care, and total health- and social care costs. This core set was developed on the basis of four sources, of which the focus groups described in the current article had the most prominent influence. The other three sources were: i) findings from a literature review conducted to develop a framework on integrated care for multi-morbidity and to identify existing programme evaluations [5], ii) (inter)national stakeholder advisory board discussions in the eight SELFIE partner countries with Patients, Partners, Professionals, Payers, and Policy makers, and iii) the aims of the aforementioned 17 programmes and current indicators included in these programmes.  A set of criteria was used to come to this list (see Supplementary File Box S.4), and some concepts mentioned in the focus group were not included in the core set, because these did not meet these criteria. This is the case for the more system- and cultural-wide concepts which cannot be used to assess the performance of one specific care programme, such as for example the absence of public stigma.

**Conclusion**

In the current study persons with multi-morbidity in eight European countries described what good health- and care means to them. Beyond traditional outcomes, aspects such as acceptance, absence of shame, and enjoyment of life came forth from the discussions. Especially important for persons with multi-morbidity was to be approached holistically and to have continuity of care. Thus a variety of health outcomes that entail well-being, social, and psychological facets should be included when evaluating integrated care programmes for persons with multi-morbidity. Even more imperative is that experience with care outcomes be included in such evaluations that tackle the complexity of multi-morbidity care provision. In conclusion, important next steps include moving towards harmonising evaluation frameworks and the specific indicators used in such evaluations.



For peer review only - http://bmjopen.bmj.com/site/about/guidelines.xhtml

**Funding statement**

The SELFIE project has received funding from the European Union's Horizon 2020 research and innovation programme under grant agreement No 634288. The content of this publication reflects only the SELFIE groups' views and the European Commission is not liable for any use that may be made of the information contained herein.

**Acknowledgements**

We would like to thank the focus group participants for making this research possible. We would also like to thank the entire SELFIE consortium for their support.

For peer review only

**Data sharing statement**

The recordings and available verbatim transcripts will not be made available, to protect the anonymity of participants. Upon request to the corresponding author, we may consider in collaboration with the respective SELFIE-partner, whether a country-specific focus group report can be shared. Other findings from the overarching SELFIE study can be found on the SELFIE website (www.selfie2020.eu).

**Competing interests**

None of the involved co-authors had a competing interest.

**Author's contribution**

FL, MB, and MRM drafted the focus group procedure document and analysis instruction document, and organised and were present at the Dutch focus group. VS, MK, SCS, AZ, RE, CV, MiH, and TC were involved in the translation and organisation of the focus groups in their respective countries and in the analysis of their focus group and reporting thereof. FL, MB, and MRM analysed the Dutch group; FL, MaH, MB, and MRM analysed all focus group reports and conducted the overarching analyses. FL, MaH, and MRM drafted the manuscript and the revision; all co-authors critically reviewed and contributed to the manuscript and revision, e.g., by appraising themes and providing fitting quotes.



For peer review only - http://bmjopen.bmj.com/site/about/guidelines.xhtml

**References**


1. van Oostrum SH, Picavet HSJ, van Gelder BM, et al. Multimorbidity and comorbidity in the Dutch population – data from general practices. BMC Public Health. 2012;12:715.
2. Barnett K, Mercer SW, Norbury M, et al. Epidemiology of multimorbidity and implications for health care, research, and medical education: a cross-sectional study. Lancet. 2012;380(9836):37-43.
3. Mair FS & May CR. Thinking about the burden of treatment. BMJ. 2014;348:g6680.
4. van der Heide I, Snoeijs S, Melchiorre MG, et al. Innovating care for people with multiple chronic conditions in Europe: An overview. Utrecht, the Netherlands: ICARE4EU, NIVEL, 2015.
5. Struckmann V, Leijten FRM, van Ginneken E, et al. Relevant models and elements of integrated care for multi-morbidity: results of a scoping review. Health Policy. 2018;122:23-35.
6. de Bruin SR, Versnel N, Lemmens LC, et al. Comprehensive care programs for patients with multiple chronic conditions: A systematic review. Health Policy. 2012;107(2-3):108-145.
7. Hopman P, de Bruin SR, Forjaz MJ, et al. Effectiveness of comprehensive care programs for patients with multiple chronic conditions or frailty: a systematic literature review. Health Policy. 2016;120:818-832.
8. Craig P, Dieppe P, Macintyre S, et al. Developing and evaluating complex interventions: the new Medical Research Council guidance. BMJ. 2008;337:a1655.
9. Berwick DM, Nolan TW, Whittington J. The Triple Aim: Care, health, and cost. Health Aff. 2008;27(3):759-769.
10. Whittington JW, Nolan K, Lewis N, et al. Pursing the Triple Aim: The first 7-years. Milbank Q. 2015;93(2):263-300.
11. Boorse C. Health as a theoretical concept. Philosophy of Science. 1977;44(4):542-573.
12. World Health Organization. WHO Constitution. 1946.
13. Huber M, Knottnerus JA, Green L, et al. How should we define health? BMJ. 2011;343:d4163.
14. Venkatapuram, S. Health Justice. Cambridge: Polity Press, 2011.
15. Glasgow RE, Wagner EH, Schaefer J, et al. Development and validation of the Patient Assessment of Chronic Illness Care (PACIC). Med Care. 2005;43:436-444.
16. Linton M, Dieppe P, Medina-Lara A. Review of 99 self-report measures for assessing well-being in adults: exploring dimensions of well-being and developments over time. BMJ Open. 2016;6:e010641.


For peer review only



For peer review only - http://bmjopen.bmj.com/site/about/guidelines.xhtml

17. Tong A, Sainsbury P, Craig J. Consolidated criteria for reporting qualitative research (COREQ): a 32-item checklist for interviews and focus groups. Int J for Qual Health Care. 2007;19:349-357.

18. World Health Organization. Informed Consent Form Template for Qualitative Studies. <http://www.who.int/rpc/research_ethics/informed_consent/en/>

19. World Health Organization (WHO). International Classification of Diseases (ICD-10). Geneva: WHO; 2010.

20. Shah KK, Mulhern B, Longworth L, Janssen MF. Views of the UK general public on important aspects of health not captured by EQ-5D. Patient. Online first, 2017. DOI: 10.1007/s40271-017-0240-1

21. Ebrahimi Z, Wilhelmson K, Moore CD, et al. Frail elders' experiences with and perceptions of health. Qual Health Res. 2012;22:1513-1523.

22. World Health Organization (WHO). International Classification of Functioning (ICF). Geneva: WHO; 2001.

For peer review only

## Tables & Figures

**Box 1:** Information on the SELFIE project

**SELFIE** (Sustainable intEgrated chronic care modeLs for multi-morbidity: delivery, FInancing, and performancE) is a Horizon2020 funded EU project that aims to contribute to the improvement of person-centred care for persons with multi-morbidity by proposing evidence-based, economically sustainable, integrated care programmes that stimulate cooperation across health and social care and are supported by appropriate financing and payment schemes. More specifically, SELFIE aims to:

- Develop a taxonomy of promising integrated care programmes for persons with multi-morbidity;
- Provide evidence-based advice on matching financing/payment schemes with adequate incentives to implement integrated care;
- Provide empirical evidence of the impact of promising integrated care on a wide range of outcomes using Multi-Criteria Decision Analysis (MCDA);
- Develop implementation and change strategies tailored to different care settings and contexts in Europe, especially Central and Eastern Europe.

The SELFIE consortium includes eight organisations in the following countries: the Netherlands (coordinator) (NL), Austria (AT), Croatia (HR), Germany (DE), Hungary (HU), Norway (NO), Spain (ES), and the UK. Each country has a unique health- and social care system, that varies in the extent to which health- and social care are integrated and that vary in how the financing is arranged. On the SELFIE website (www.selfie2020.eu) a macro level description of the systems can be found that provides background context.

Seventeen promising integrated care programmes for persons with multi-morbidity across these eight countries are being evaluated in SELFIE. The evaluations apply MCDA and each use a common set of core outcomes as well as programme-type specific outcomes. The latter depend on whether a programme is i) a population health management programme, ii) a programme targeting frail elderly, iii) a programme targeting persons with problems in multiple life domains, or iv) an oncology or palliative care programme.

[Grant Agreement No 634288]

**Table 1:** Focus group participant characteristics

| | # (M/F) | Mean age (Min-Max) | Mean number of morbidities |
|---|---|---|---|
| **Austria (AT)** | 7 (5/2) | 72.9 (62-84) | 2.3 |
| **Croatia (HR)** | 7 (4/3) | 51.7 (31-69) | 3 |
| **Germany (DE)** | 12 (4/8) | 62.4 (37-78) | 3.9 |
| **Hungary (HU)** | 6 (1/5) | 64.5 (47-78) | 3 |
| **Netherlands (NL)** | 7 (5/2) | 66.3 (53-75) | 5 |
| **Norway (NO)** | 7 (2/5) | 65.4 (42-76) | 3.1 |
| **Spain (ES)** | 6 (5/1) | 70.2 (60-81) | 2.7 |
| **United Kingdom (UK)** | 6 (4/2) | 68.8 (58-86) | 4 |
| **Total** | 58 (30/28) | 65.3 (37-86) | 3.4 |



For peer review only - http://bmjopen.bmj.com/site/about/guidelines.xhtml

**Table 2:** Morbidities of participants per country categorised according to the ICD-10

| | **Total** | **Austria** | **Croatia** | **Germany** | **Hungary** | **Netherlands** | **Norway** | **Spain** | **UK** |
|---|---|---|---|---|---|---|---|---|---|
| **Neoplasms** | 8 | 3 | 1 | 2 | | 1 | | 1 | |
| Diseases of the **blood** and disorders involving the immune mechanism | 3 | | 2 | | | | 1 | | |
| **Endocrine**, nutritional, and metabolic disease | 31 | 2 | 1 | 6 | 5 | 5 | 4 | 3 | 5 |
| **Mental** and **behavioural** disorders | 6 | | | | | | | | 6 |
| Diseases of the **nervous** system | 20 | 1 | 2 | 12 | 2 | | 1 | | 2 |
| Diseases of the **eye** and adnexa | 7 | | | | 1 | 6 | | | |
| Diseases of the **ear** and mastoid process | 5 | | | 3 | | | 1 | | 1 |
| Diseases of the **circulatory** system | 44 | 1 | 5 | 12 | 8 | 7 | 4 | 4 | 3 |
| Diseases of the **respiratory** system | 17 | | 1 | | | 6 | 4 | 6 | |
| Diseases of the **digestive** system | 10 | 1 | 1 | 1 | 1 | 2 | 1 | 1 | 2 |
| Diseases of the **skin** and subcutaneous tissue | 3 | 1 | 1 | | | | | | 1 |
| Diseases of the **musculoskeletal system** and connective tissue | 36 | 6 | 6 | 9 | 1 | 4 | 5 | 1 | 4 |
| Diseases of the **genitourinary** system | 4 | | | | | 4 | | | |
| **Congenital malformations**, **chromosomal abnormalities** | 1 | 1 | | | | | | | |
| Symptoms, signs, **abnormal clinical and lab findings**, **not else classified** | 2 | | | 2 | | | | | |
| **Injury**, **poisoning** and other consequences of external causes | 1 | | | | | | 1 | | |

Note: ICD-10: International Statistical Classification of Diseases and Related Health Problems, 10th revision.

For peer review only

**Table 3:** Most frequently listed outcomes on the 'top-10' lists of focus group participants across the eight focus groups

| | # / 58 | Austria (AT) | Croatia (HR) | Germany[1] (DE) | Hungary (HU) | Netherlands (NL) | Norway (NO) | Spain (ES) | United Kingdom (UK) |
|---|---|---|---|---|---|---|---|---|---|
| *Health and well-being* | | | | | | | | | |
| Energy & fatigue | **12** | 4/7 | | | 1/6 | 4/7 | 2/7 | | 1/6 |
| Feeling safe | **10** | | 1/7 | | 3/6 | 3/7 | | 3/6 | |
| Cognitive functioning | **12** | 4/7 | | 1/6 | | 2/7 | 1/7 | 3/6 | 1/6 |
| Maintaining independence | **16** | | 2/7 | 5/6 | 1/6 | 1/7 | | 1/6 | 1/6 |
| Enjoyment of life | **16** | 2/7 | | 4/6 | 1/6 | 3/7 | 3/7 | | 3/6 |
| Positive frame of mind, resilience | **16** | 2/7 | 3/7 | 2/6 | 2/6 | 1/7 | 2/7 | 2/6 | 2/6 |
| Self-esteem | **11** | 1/7 | 1/7 | 3/6 | | 3/7 | 2/7 | | 1/6 |
| Social relationships | **17** | 3/7 | | 5/6 | 2/6 | 4/7 | | 2/6 | 1/6 |
| Societal participation | **12** | 1/7 | 3/7 | 3/6 | | 3/7 | | 1/6 | 1/6 |
| *Experience* | | | | | | | | | |
| Individualised care planning / tailored care | **13** | 1/7 | | 4/6 | 1/6 | 2/7 | 4/7 | | 1/6 |
| Holistic assessment/understanding | **11** | 1/7 | 1/7 | 4/6 | 2/6 | 2/7 | | | 1/6 |
| Good communication between provider-patient | **14** | 1/7 | 1/7 | 2/6 | | 2/7 | 1/7 | 3/6 | 4/6 |
| Shared decision-making professional-patient | **13** | 2/7 | | 4/6 | 1/6 | 1/7 | 1/7 | | 4/6 |
| Respectful interaction between professional-patient | **12** | 2/7 | 1/7 | 5/6 | | | | | 4/6 |
| Shared information between professionals | **10** | 2/7 | 1/7 | 1/6 | 2/6 | 2/7 | | 1/6 | 1/6 |
| Team work between professionals & providers | **10** | | 1/7 | | 2/6 | | 2/7 | 2/6 | 3/6 |
| Confidence in knowledge and skills in professionals | **10** | 1/7 | 2/7 | 3/6 | 2/6 | 1/7 | | 1/6 | |
| Pro-active, prevention-oriented, care | **13** | | 1/7 | 1/6 | 4/6 | 4/7 | | | 3/6 |

Note: 1 = Group split into 2, so each time # out of 6 persons, each group discussed either health/well-being and costs or care and costs.

For peer review only



For peer review only - http://bmjopen.bmj.com/site/about/guidelines.xhtml

**Supplementary files** accompanying the manuscript:

Defining good health and care from the perspective of persons with multi-morbidity: Results from a qualitative study of focus groups in eight European countries

**Box S.1:** Extended protocol of the focus group

**Introduction**

- Purpose: to discuss what is important from the perspective of an individual with multiple health- and/or social problems in health/well-being and in care
- Overarching aim: the results of the discussion will be used to improve the care for persons with multiple health- and social problems
- Agenda explanation
- 'Rules'
    - No right / wrong answers, only differing points of view
    - Not necessary to agree with others, but must listen respectfully as others share their views
    - Talk to one-another, researchers only have role as moderator in guiding the discussion
    - Privacy / anonymity
    - The discussion will be recorded, and notes will be made
    - Turn off phones
- Informed consent
- Introduction of participants to one-another

**Part 1: Defining good health/well-being and care**

Step i):

- When you think about your health and well-being: try to complete this sentence / thought: "For me, being in great health means…"
- When you think about the care that you receive, have received, or may receive in the future, try to complete this sentence / thought: "I'd be really satisfied with all of the care / the overall care that I receive, if…"

**Write this question on a large white board / flip-over*

- Provide examples if participants are having trouble understanding what you mean:
    - …being able to work full-time. (health)
    - …being able to spend time with family and friends. (health)
    - …being able to live for many more years. (health)
    - …not going to the doctor so often. (health)
    - …my doctor took more time to explain things to me. (care)
    - …my partner would always be allowed to join me and discuss with the doctors as well. (care)
    - …my care plan was discussed with me. (care)
    - …I had one overall written care plan. (care)
    - …I always knew I could contact one person in case of questions. (care)

**The researcher needs to ask triggering and thought-provoking questions here.*

**The discussion should be about health and well-being in general, not about care specifically (yet).*

**Be careful not to provide too many examples and to steer the answers in one direction.*

**The researcher leading the discussion will be writing these thoughts out on a white board / flip-over.*

**The researchers need to allow the participants to reflect on one-another's ideas.*

For peer review only

*Close the discussion with a final question: i.e., have we missed anything?*

Step ii):

- Once it seems as though all participants have said what is important to them, the researcher will go through the points on the board, and with the help of the participants try to extrapolate what these things mean, in order to go from **specific** examples to **general** outcomes**.** For example:
  - "For me, being in good health means being able to work full-time", does this mean:
    - Participating in society?
    - Being able to support one-self financially? → Self-sufficiency
    - Having social contacts?
    - Having a daily structure?
  - "For me, being in good health means not going to the doctor so often", does this mean:
    - Having no acute health problems?
    - Having the ability to deal with minor health problems on your own? (self-management)
  - "I'd be really satisfied with all of the care / the overall care that I receive, if my doctor took more time to explain things to me", does this mean:
    - Good communication?
    - Shared-decision making?
    - More/enough time?
  - "I'd be really satisfied with all of the care / the overall care that I receive, if my partner could always be allowed to join me and discuss with the doctors as well.", does this mean:
    - Understanding your personal situation?
    - Involvement of the informal caregiver?
    - Good communication?

*The researcher needs to write key words that come forth on large notecards and place them in the centre of the table for all participants to be able to see.*

*The researcher needs to ask triggering and thought-provoking questions here – what do the points mentioned in step 1 really mean? What does it come down to? What would it mean for the person sitting next to you? How can you generalise this so that it is applicable for others in the room? Try to distinguish between individual opinions and actual group consensus.*

*In asking further questions, try to focus on multi-morbidity – how is this specific to you as a person with multiple health- and/or social problems, would this be different if you had a single disease?*

*For each initial statement, multiple cards can be made.*

*Close the discussion with a final question: i.e., have we covered all elements?*

**Break**

*Place cards from part one onto the table, sort them by 'triple aim'*

*Sort through original 77 cards, and add cards to the table from these (refrain from overlap, choose card from part one in words of participants over literature-cards)*

For peer review only

**Part 2: Selecting most important concepts**

- Instructions: "In the discussion during part 1 you've come up with points that are important for you when it comes to defining health and care. As you saw, we've been trying to write this into more general terms on these cards."
- In our research team we've also been looking at concepts that are often mentioned in research/literature as being important, we've added these to the table as well."

**Explain the new concepts in case anything is unclear.*

- "We'd like to ask each of you to look at all of these cards and write down for yourself which 10 are most important to you. This can be a mix of health/well-being, care, and cost cards."
- "Now we'd like you all to discuss your top 10 and try to see whether you can reach a group consensus, and make a top 10 of cards at the centre of the table."

**Ask a participant to first put his/hers down or read his/her list first, through discussion see whether some kind of consensus can be reached.*

**Box S.2:** List of literature-based outcomes used on cards in part two

**Health / well-being**

- Biomedical outcomes: e.g., blood sugar, blood pressure, lung function, kidney function
- Life expectancy
- Maintaining independence
- Activities of daily living: e.g. eating, bathing
- Independence from medical aids: e.g., wheelchair, oxygen
- Energy and fatigue
- Pain and discomfort
- Disability
- Physical mobility: e.g., being able to walk
- Work ability: being physically and mentally able to work
- Cognitive functioning: e.g., thinking, memory, concentration and learning
- Anxiety and depression
- Stress
- Worrying
- Listless: no spirit, apathetic, indifferent
- Self-esteem: a good feeling of one-self, self-respect
- Respect from others
- Social relationships: staying in contact with family/friends/acquaintances
- Maintaining social status: in terms of 'societal class', 'occupation', how you think others look at you in society.
- Societal participation: e.g., volunteer work, club memberships, community activities
- Social support: emotional, instrumental and financial assistance from the social network
- Loneliness
- Investing in future health and wellbeing
- Self-efficacy: belief in one's capacity to follow a needed or desired course of action
- Positive frame of mind, resilience: ability to maintain a positive perspective regarding the future, despite setbacks or change
- Coping
- Being able to deal with problems, losses, adverse events in an effective manner

For peer review only

| |
|---|
| • Health literacy: capacity to obtain, process, and understand information about illness and health<br>• Compliance/adherence to treatment<br>• Lifestyle: e.g., smoking, nutrition, physical activity, alcohol and drug use<br>• Feeling safe<br>• Enjoyment of life<br>• Maintaining dignity<br>• Living comfortably<br>• Caregiver burden |
| **Experience with care** |
| • Holistic assessment<br>• Shared decision-making<br>• Tailored care<br>• Individualised care planning<br>• Pro-active, prevention-oriented care<br>• Clear responsibilities and accountability<br>• Named coordinator<br>• Team work and collaboration between professionals<br>• Good communication<br>• Shared information between professionals and providers<br>• Continuity of care: pertains to continuity (e.g., follow-up, monitoring, stability, transfer) throughout time and between professionals and organisations)<br>• Confidence in knowledge and skills in professionals<br>• Respectful interaction between provider-patient: feeling of being treated with respect and being 'heard'<br>• Geographical access: e.g., facilities close to home<br>• Physical access: e.g., building is wheelchair accessible<br>• Timely access: e.g., no/short waiting lists<br>• Satisfaction with care process |
| **Costs** |
| • Service and support coverage: co-payments/deductible and financial access to services<br>• Travel and parking costs<br>• Loss of income – patient<br>• Loss of income - informal caregiver |

For peer review only

**Box S.3:** Most important concepts in health/well-being, care, and costs per focus group participant per country (part 2 of the focus groups)

| | # / 58 | Austria | Croatia | Germany[1] | Hungary | Netherlands | Norway | Spain | UK |
|---|---|---|---|---|---|---|---|---|---|
| *Health and well-being* | | | | | | | | | |
| **Physical well-being** | | | | | | | | | |
| Energy & fatigue | **12** | 4/7 | | | 1/6 | 4/7 | 2/7 | | 1/6 |
| Pain & discomfort | **4** | 3/7 | | | | | | | 1/6 |
| Biomedical outcomes | **3** | 2/7 | | | 1/6 | | | | |
| Disability | **5** | | 2/7 | | 1/6 | | 1/7 | | 1/6 |
| Progression free health state* | **2** | | | | 2/6 | | | | |
| Physical mobility | **5** | | 1/7 | 1/6 | 1/6 | 1/7 | 2/7 | | |
| Activities of daily living | **7** | | 1/7 | 3/6 | 1/6 | 1/7 | | | 1/6 |
| Life expectancy | **1** | | | | | 1/7 | | | |
| To be active* | **2** | | | | | | 2/7 | | |
| **Feeling safe** | **10** | | 1/7 | | 3/6 | 3/7 | | 3/6 | |
| Discrimination* | **3** | | 3/7 | | | | | | |
| **Cognitive functioning** | **12** | 4/7 | | 1/6 | | 2/7 | 1/7 | 3/6 | 1/6 |
| **Independence** | | | | | | | | | |
| Being able to choose what you do / want to do* | **1** | | | | | | | 1/6 | |
| Moving freely around street * | **1** | | | | | | | 1/6 | |
| Independence from medical aids | **3** | | | | 1/6 | | | 1/6 | 1/6 |
| Maintaining independence | **16** | | 2/7 | 5/6 | 1/6 | 1/7 | | 1/6 | 1/6 |
| Living comfortably | **5** | 1/7 | | 2/6 | | 1/7 | | | 1/6 |
| **Psychological well-being*** | **3** | | | 3/6 | | | | | |
| Listlessness | **3** | 2/7 | | | | | | 1/6 | |
| Loneliness | **2** | 2/7 | | | | | | | |
| Empathy* | **1** | | 1/7 | | | | | | |
| Stress/worrying | **7** | | | | 3/6 | | 1/7 | | 3/6 |
| Anxiety & depression | **2** | | | | 1/6 | | | | 1/6 |
| **Enjoyment of life** | **16** | 2/7 | | 4/6 | 1/6 | 3/7 | 3/7 | | 3/6 |
| **Positive frame of mind, resilience** | **16** | 2/7 | 3/7 | 2/6 | 2/6 | 1/7 | 2/7 | 2/6 | 2/6 |
| Creativity* | **2** | | 2/7 | | | | | | |
| Investing in future | **1** | | | | | | | 1/6 | |
| Coping | **5** | 1/7 | | | | | 2/7 | 1/6 | 1/6 |
| Maintaining dignity | **9** | 1/7 | | | 4/6 | | | 2/6 | 2/6 |
| Self-esteem | **11** | 1/7 | 1/7 | 3/6 | | 3/7 | 2/7 | | 1/6 |
| Acceptance* | **4** | | | 3/6 | | 1/7 | | | |
| Look out for possibilities* | **1** | | | | | | 1/7 | | |
| Hope | **1** | | | | | | 1/7 | | |

For peer review only

| | # / 58 | Austria | Croatia | Germany[1] | Hungary | Netherlands | Norway | Spain | UK |
|---|---|---|---|---|---|---|---|---|---|
| **Social relationships & participation** | | | | | | | | | |
| Social relationships | **17** | 3/7 | | 5/6 | 2/6 | 4/7 | | 2/6 | 1/6 |
| Societal participation | **12** | 1/7 | 3/7 | 3/6 | | 3/7 | | 1/6 | 1/6 |
| Respect from others | **6** | | | | | 2/7 | 2/7 | 1/6 | 1/6 |
| To be taken seriously* | **3** | | | | | | 3/7 | | |
| Social support | **5** | 1/7 | 1/7 | | | | 2/7 | 1/6 | |
| Being able to relate to other people without physical limits* | **1** | | | | | | | 1/6 | |
| Feeling useful* | **2** | | | | | | | 2/6 | |
| Get something off one's chest* | **2** | 2/7 | | | | | | | |
| (Maintaining good) social status | **5** | 2/7 | | 3/6 | | | | | |
| Not being reduced to the disease* | **6** | 1/7 | | | | | | | |
| To be regarded as whole human being* | | | | | | | 4/7 | | |
| Seeing past the outside/surface of a person* | | | | | | | 1/7 | | |
| Surrounding area takes care of someone's needs* | **1** | 1/7 | | | | | | | |
| Treatments compatible with work* | **1** | 1/7 | | | | | | | |
| Work ability | **6** | | 3/7 | | 1/6 | 2/7 | | | |
| Social expectations* | **1** | | 1/7 | | | | | | |
| Daily structure* | **4** | | | 4/6 | | | | | |
| Being able to plan* | **2** | | | | | | 2/7 | | |
| Burden for informal caregivers* | **3** | | | 2/6 | | | 1/7 | | |
| **Self-management*** | **7** | | | 6/6 | | 1/7 | | | |
| Lifestyle | **3** | 2/7 | | | | | | | 1/6 |
| Compliance/adherence to treatment | **5** | 1/7 | | 2/6 | 1/6 | | | | 1/6 |
| Self-efficacy | **6** | | 2/7 | | 1/6 | | | 1/6 | 2/6 |
| Investing in future health & well-being | **4** | | 1/7 | 2/6 | | 1/7 | | | |
| Health literacy | **7** | | | 5/6 | 1/6 | 1/7 | | | |
| **Daily quality of life** | **1** | | | | | | 1/7 | | |
| *Experience* | | | | | | | | | |
| **Individualised care planning / tailored care** | **13** | 1/7 | | 4/6 | 1/6 | 2/7 | 4/7 | | 1/6 |
| **Holistic assessment/understanding** | **11** | 1/7 | 1/7 | 4/6 | 2/6 | 2/7 | | | 1/6 |
| **Good interactions providers-patients** | | | | | | | | | |
| Good communication | **14** | 1/7 | 1/7 | 2/6 | | 2/7 | 1/7 | 3/6 | 4/6 |
| Encourage patients* | **1** | | | | | | | 1/6 | |
| Psychological support* | **2** | | | 1/6 | | 1/7 | | | |
| Optimist reactions* | **1** | | | | | | | 1/6 | |
| Shared decision-making | **13** | 2/7 | | 4/6 | 1/6 | 1/7 | 1/7 | | 4/6 |
| Respectful interaction between provider-patient | **12** | 2/7 | 1/7 | 5/6 | | | | | 4/6 |
| Trust* | **7** | | | 3/6 | | 4/7 | | | |
| Predictability of care service delivery* (good info) | **1** | | | | 1/6 | | | | |
| Flow of information* | **2** | | | | | | 2/7 | | |

For peer review only

| | # / 58 | Austria | Croatia | Germany[1] | Hungary | Netherlands | Norway | Spain | UK |
|---|---|---|---|---|---|---|---|---|---|
| Being listened to and believed* | **2** | | | | | | | | 2/6 |
| **Good collaborations providers** | | | | | | | | | |
| Shared information between providers* | **10** | 2/7 | 1/7 | 1/6 | 2/6 | 2/7 | | 1/6 | 1/6 |
| Data transparency* | **3** | | | 3/6 | | | | | |
| Team work between providers | **10** | | 1/7 | | 2/6 | | 2/7 | 2/6 | 3/6 |
| Solidary distribution between funds* | **1** | 1/7 | | | | | | | |
| Clear responsibility and accountability | **5** | | 1/7 | 3/6 | | | | | 1/6 |
| Named coordinator | **7** | | 1/7 | 4/6 | | 1/7 | 1/7 | | |
| **Continuity of care** | **9** | | 2/7 | 2/6 | 3/6 | 1/7 | | 1/6 | |
| Collaboration across sectors* | **5** | | | 4/6 | | | | | 1/6 |
| Coordination between primary care and hospitals* | **1** | | | | | | | 1/6 | |
| Prevent and avoid going to ER* | **2** | | | | | | | 2/6 | |
| Administrative barriers* | **1** | | 1/7 | | | | | | |
| Transferring of responsibility* | **1** | | 1/7 | | | | | | |
| Transfer-care* | **2** | | | 1/6 | | 1/7 | | | |
| After-care* | **2** | | | 1/6 | | 1/7 | | | |
| Systematic operating procedures in care* | **3** | | | | 3/6 | | | | |
| Utilisation of EMRs* | **2** | | | 3/6 | 2/6 | 2/7 | | | |
| Careful use of patient files / privacy | **2** | | | 2/6 | | | | | |
| **Confidence in knowledge and skills in providers** | **10** | 1/7 | 2/7 | 3/6 | 2/6 | 1/7 | | 1/6 | |
| Education of health professionals* | **3** | | 3/7 | | | | | | |
| Importance and function of centres of excellence* | **1** | | 1/7 | | | | | | |
| Broader specialisations* | **1** | | | | | | 1/7 | | |
| **Access & availability** | | | | | | | | | |
| Physical access | **5** | | 1/7 | 1/6 | | 2/7 | | 1/6 | |
| Timely access* (i.e., short waiting times) | **9** | | 2/7 | 1/6 | 2/6 | 1/7 | | | 4/6 |
| Enough time* (provider for patient) | **8** | 4/7 | | | 1/6 | 1/7 | | 2/6 | |
| Being seen by specialist when needed* | **2** | | | | | | | 2/6 | |
| Geographical access | **3** | | 1/7 | | | | | 1/6 | 1/6 |
| Availability of treatment* | **3** | | 3/7 | | | | | | |
| Home care (available) | **1** | | | 1/6 | | | | | |
| Transport* | **1** | | | 1/6 | | | | | |
| **Avoid unnecessary treatment*** | **2** | 2/7 | | | | | | | |
| **Pro-active, prevention-oriented, care** | **13** | | 1/7 | 1/6 | 4/6 | 4/7 | | | 3/6 |
| **Patient-education and support (groups)** | | | | | | | | | |
| Integration of self-help groups* | **3** | 3/7 | | | | | | | |
| Importance of associations of patients* | **1** | | 1/7 | | | | | | |
| Good trainings offered* | **1** | 1/7 | | | | | | | |
| Medication help* | **5** | | | 1/6 | | 4/7 | | | |
| Self-management support* | **1** | | | | | 1/7 | | | |

For peer review only

| | # / 58 | Austria | Croatia | Germany[1] | Hungary | Netherlands | Norway | Spain | UK |
|---|---|---|---|---|---|---|---|---|---|
| Good info for patients on health | **1** | | | | | | 1/7 | | |
| Family support* | **3** | | 3/7 | | | | | | |
| **Bureaucratic hurdles*** | **5** | | | 4/6 | | 1/7 | | | |
| **Physical surroundings** | | | | | | | | | |
| Material conditions in institutions* | **1** | | 1/7 | | | | | | |
| **Macro-level** | | | | | | | | | |
| Non-cooperation with health policy makers* | **2** | | 2/7 | | | | | | |
| Importance of media pressure | **2** | | 2/7 | | | | | | |
| Learning from different countries' experiences* | **1** | 1/7 | | | | | | | |
| **Overall satisfaction with care process** | **8** | 1/7 | | 4/6 | 2/6 | | | 1/6 | |
| *Costs* | | | | | | | | | |
| **Loss of income** | | | | | | | | | |
| Loss of income informal caregiver | **3** | | 1/7 | | | | | 2/6 | |
| Loss of income patient | **7** | 1/7 | 3/7 | | 1/6 | | | 1/6 | 1/6 |
| Overall costs in relation to income* | **1** | | | | | 1/7 | | | |
| **Service and support coverage** | **6** | 2/7 | 1/7 | 1/6 | 1/6 | | 1/7 | | |
| **Need for cost-benefit analysis*** | **2** | | 2/7 | | | | | | |
| **Money follows the patient*** (bundled type payment that crosses all care level-borders) | **1** | | 1/7 | | | | | | |
| **Travel and parking costs** | **2** | | | | 1/6 | | | | 1/6 |

Note: 1 = Group split into 2, so each time # out of 6 persons, each group discussed either health/well-being and costs or care and costs. Btw = between, Profs = professional care providers, EMRs = Electronic Medical Records, ER = Emergency Room. * Concepts/phrases that were novel and written on cards based on part 1 of the focus groups, and not on the basis of the a priori literature search.

**Box S.4:** Criteria used to come to a core set of outcomes to evaluate integrated care programmes

- **Cover the Triple Aim**: 1) improving population health/well-being and 2) patient experience, and 3) reducing cost (growth);
- **Measurable at an individual level** using PROMS and PREMS (this only applies to the first and second of the three aims);
- **Relevant for multi-morbidity** in different contexts and population groups;
- **Relevant for the 5P stakeholders: p**atients (persons with multi-morbidity), **p**artners (informal caregivers), **p**rofessionals, **p**ayers, **p**olicy makers.
- **Relevant across the** 17 selected **promising integrated care programmes**;
- **Timing of intervention effects**: the intervention effect (i.e., the impact of a programme on the outcome) in newly enrolled patients is likely to start occurring within the SELFIE evaluation period (6-18 months);
- The **outcomes are not redundant**, meaning that they are all necessary and not superfluous (e.g., one outcome does not overlap much with another outcome);
- **Preference independence**, i.e., the weight of one outcome can be elicited independently from the performance of another outcome;
- **Feasible to measure** performance.

For peer review only

# COREQ (COnsolidated criteria for REporting Qualitative research) Checklist

A checklist of items that should be included in reports of qualitative research. You must report the page number in your manuscript where you consider each of the items listed in this checklist. If you have not included this information, either revise your manuscript accordingly before submitting or note N/A.

| Topic | Item No. | Guide Questions/Description | Reported on Page No. |
|---|---|---|---|
| **Domain 1: Research team and reflexivity** | | | |
| *Personal characteristics* | | | |
| Interviewer/facilitator | 1 | Which author/s conducted the interview or focus group? | 8 |
| Credentials | 2 | What were the researcher's credentials? E.g. PhD, MD | 8 (1) |
| Occupation | 3 | What was their occupation at the time of the study? | 8 (1) |
| Gender | 4 | Was the researcher male or female? | 11 |
| Experience and training | 5 | What experience or training did the researcher have? | 8 |
| *Relationship with participants* | | | |
| Relationship established | 6 | Was a relationship established prior to study commencement? | 7 |
| Participant knowledge of the interviewer | 7 | What did the participants know about the researcher? e.g. personal goals, reasons for doing the research | 8 |
| Interviewer characteristics | 8 | What characteristics were reported about the inter viewer/facilitator? e.g. Bias, assumptions, reasons and interests in the research topic | 8, 11 |
| **Domain 2: Study design** | | | |
| *Theoretical framework* | | | |
| Methodological orientation and Theory | 9 | What methodological orientation was stated to underpin the study? e.g. grounded theory, discourse analysis, ethnography, phenomenology, content analysis | 9 |
| *Participant selection* | | | |
| Sampling | 10 | How were participants selected? e.g. purposive, convenience, consecutive, snowball | 7 |
| Method of approach | 11 | How were participants approached? e.g. face-to-face, telephone, mail, email | 7 |
| Sample size | 12 | How many participants were in the study? | 11 |
| Non-participation | 13 | How many people refused to participate or dropped out? Reasons? | 11 |
| *Setting* | | | |
| Setting of data collection | 14 | Where was the data collected? e.g. home, clinic, workplace | 7 |
| Presence of non-participants | 15 | Was anyone else present besides the participants and researchers? | 7, 11 |
| Description of sample | 16 | What are the important characteristics of the sample? e.g. demographic data, date | 7, 11 |
| *Data collection* | | | |
| Interview guide | 17 | Were questions, prompts, guides provided by the authors? Was it pilot tested? | 7-8 |
| Repeat interviews | 18 | Were repeat inter views carried out? If yes, how many? | 7-8 |
| Audio/visual recording | 19 | Did the research use audio or visual recording to collect the data? | 8 |
| Field notes | 20 | Were field notes made during and/or after the inter view or focus group? | 8-10 |
| Duration | 21 | What was the duration of the inter views or focus group? | 11 |
| Data saturation | 22 | Was data saturation discussed? | 9 |
| Transcripts returned | 23 | Were transcripts returned to participants for comment and/or | 9-10 |

For peer review only - http://bmjopen.bmj.com/site/about/guidelines.xhtml

| Topic | Item No. | Guide Questions/Description | Reported on Page No. |
|---|---|---|---|
| | | correction? | |
| **Domain 3: analysis and findings** | | | |
| *Data analysis* | | | |
| Number of data coders | 24 | How many data coders coded the data? | 9 |
| Description of the coding tree | 25 | Did authors provide a description of the coding tree? | 9 |
| Derivation of themes | 26 | Were themes identified in advance or derived from the data? | 9 |
| Software | 27 | What software, if applicable, was used to manage the data? | 9 |
| Participant checking | 28 | Did participants provide feedback on the findings? | 9-10 |
| *Reporting* | | | |
| Quotations presented | 29 | Were participant quotations presented to illustrate the themes/findings? Was each quotation identified? e.g. participant number | 9-10 (results) |
| Data and findings consistent | 30 | Was there consistency between the data presented and the findings? | 9-10 |
| Clarity of major themes | 31 | Were major themes clearly presented in the findings? | 9-10 |
| Clarity of minor themes | 32 | Is there a description of diverse cases or discussion of minor themes? | 9-10 |

Developed from: Tong A, Sainsbury P, Craig J. Consolidated criteria for reporting qualitative research (COREQ): a 32-item checklist for interviews and focus groups. *International Journal for Quality in Health Care*. 2007. Volume 19, Number 6: pp. 349 – 357

**Once you have completed this checklist, please save a copy and upload it as part of your submission. DO NOT include this checklist as part of the main manuscript document. It must be uploaded as a separate file.**

For peer review only

BMJ Open

# Defining good health and care from the perspective of persons with multi-morbidity: Results from a qualitative study of focus groups in eight European countries

| | |
|---|---|
| Journal: | *BMJ Open* |
| Manuscript ID | bmjopen-2017-021072.R2 |
| Article Type: | Research |
| Date Submitted by the Author: | 30-May-2018 |
| Complete List of Authors: | Leijten, Fenna; Erasmus Universiteit Rotterdam, Erasmus School of Health Policy & Management<br>Hoedemakers, Maaike; Erasmus Universiteit Rotterdam, Erasmus School of Health Policy & Management<br>Struckmann, Verena; Berlin University of Technology, Department of Health Care Management<br>Kraus, Markus; Institute of Advanced Studies<br>Cheraghi-Sohi, Sudeh; University of Manchester, NIHR Greater Manchester Patient Safety Translational Research Centre<br>Zemplenyi, Antal; Syreon Research Institute; University of Pécs, Healthcare Financial Management Department<br>Ervik, Rune; Uni Research Rokkan Centre<br>Vallve, Claudia; Consorci Institut D'Investigacions Biomediques August Pi i Sunyer (IDIBAPS)<br>Huic, Mirjana; Agency for Quality and Accreditation in Health Care and Social Welfare<br>Czypionka, Thomas; Institute of Advanced Studies<br>Boland, Melinde; Erasmus Universiteit Rotterdam, Erasmus School of Health Policy & Management<br>Rutten-van Mölken, Maureen; Erasmus Universiteit Rotterdam, Erasmus School of Health Policy & Management; Institute for Medical Technology Assessment, Erasmus University |
| <b>Primary Subject Heading</b>: | Patient-centred medicine |
| Secondary Subject Heading: | General practice / Family practice, Health economics, Health services research, Public health, Qualitative research |
| Keywords: | PREVENTIVE MEDICINE, QUALITATIVE RESEARCH, person-centered, multi-morbidity, evaluation outcomes, integrated care |
| | |

SCHOLARONE™ Manuscripts

**Manuscript title:** Defining good health and care from the perspective of persons with multi-morbidity: Results from a qualitative study of focus groups in eight European countries


## Authors

Fenna Leijten[a], Maaike Hoedemakers[a], Verena Struckmann[b], Markus Kraus[c], Sudeh Cheraghi-Sohi[d], Antal Zemplényi[e,f], Rune Ervik[g], Claudia Vallvé[h], Mirjana Huić[i], Thomas Czypionka[c], Melinde Boland[a], Maureen Rutten-van Mölken[a,j]*, on behalf of the SELFIE consortium

## Affiliations

a) Erasmus University Rotterdam, Erasmus School of Health Policy & Management, Rotterdam, the Netherlands
b) Berlin University of Technology, Department of Health Care Management, Germany
c) Institute of Advanced Studies, Vienna, Austria
d) University of Manchester, NIHR Greater Manchester Patient Safety Translational Research Centre, Manchester, United Kingdom
e) Syreon Research Institute, Budapest, Hungary
f) University of Pécs, Healthcare Financial Management Department, Pécs, Hungary
g) Uni Research Rokkan Centre, Bergen, Norway
h) Consorci Institut D'Investigacions Biomediques August Pi i Sunyer (IDIBAPS), Barcelona, Spain
i) Agency for Quality and Accreditation in Health Care and Social Welfare, Zagreb, Croatia
j) Institute for Medical Technology Assessment, Erasmus University Rotterdam, Rotterdam, the Netherlands

*Corresponding author: Maureen Rutten-van Mölken: m.rutten@bmg.eur.nl / Tel: +31 10 4088559


For peer review only



For peer review only - http://bmjopen.bmj.com/site/about/guidelines.xhtml

## Abstract

**Objectives**: The prevalence of multi-morbidity is increasing in many Western countries. Persons with multi-morbidity often experience a lack of alignment in the care that multiple health- and social care organisations provide. As a response, integrated care programmes are appearing. It is a challenge to evaluate these and to choose appropriate outcome measures. Focus groups were held with persons with multi-morbidity in eight European countries to better understand what good health and a good care process means to them and to identify what they find most important in each.

**Methods**: In 2016, eight focus groups were organised with persons with multi-morbidity in: Austria, Croatia, Germany, Hungary, the Netherlands, Norway, Spain, and the UK (total N=58). Each focus group followed the same two-part procedure: 1) defining a) good health and well-being, b) a good care process and 2) group discussion on prioritising the most important concepts derived from part one and from a list extracted from the literature. Inductive and deductive analyses were done.

**Results**: Overall, the participants in all focus groups concentrated more on the care process than on health. Persons with multi-morbidity defined good health as being able to conduct and plan normal daily activities, having meaningful social relationships, and accepting the current situation. Absence of shame, fear, and/or stigma, being able to enjoy life, and overall psychological well-being were also important facets of good health. Being approached holistically by care professionals was said to be vital to a good care process. Continuity of care and trusting professionals were also described as important. Across countries little variation in health definitions were found but variation in defining a good care process was seen.

**Conclusion**: A variety of health outcomes that entail well-being, social, and psychological facets and especially experience with care outcomes should be included when evaluating integrated care programmes for persons with multi-morbidity.




For peer review only - http://bmjopen.bmj.com/site/about/guidelines.xhtml

**Strengths and limitations of this study**

- Focus groups were held in 8 European countries amongst persons with multi-morbidity.
- The qualitative, focus group, approach applied in this study ensures that the perspectives of persons with multi-morbidity are incorporated in the discussion on what meaningful evaluation outcomes are.
- Only participants in one focus group (in the UK) explicitly named clinical psychological health problems as a co-morbidity, which may suggest that perspectives from persons with this type of health problem are under-represented in this study.
- Creating an overarching top-10 list of important outcomes in health and care is a summation of a qualitative process and thus this list should be interpreted cautiously.
- Although different countries and multi-morbidities were included, the applied qualitative method does not mean that the findings are necessarily transferable to all persons with multi-morbidity in all countries.

For peer review only

## Introduction

With ageing populations in Western societies, there is an increasing prevalence of multi-morbidity, i.e., the co-occurrence of two or more health problems within one person at one time [1, 2]. Persons with multi-morbidity therefore often require care from different types of professionals. These professionals may work in different healthcare sectors (e.g., primary and secondary care) and may also work in social or community care. It is important that persons with multi-morbidity receive well-integrated care, in order to avoid the risk of fragmentation or overlap in the care received and interactions in treatment [3].

Integrated care is defined as structured efforts to provide coordinated, pro-active, person-centred, multidisciplinary care by two or more care professionals that effectively communicate and collaborate. There are different integrated care programmes for multi-morbidity being implemented across Europe that may offer a solution to the aforementioned risks that this population faces [4-7]. The evidence-base of such programmes is still limited and findings are not yet wholly convincing [5-7]. Increasing this evidence-base is important for the durability, wider implementation, and more sustainable reimbursement/financing of such programmes. Often, decisions on these matters are informed by economic evaluations in which costs per Quality Adjusted Life Years (QALYs) are calculated. However, it can be questioned whether the current (economic) evaluation framework provides sufficient insight into the broad range of outcomes that such integrated care programmes aim to improve. Integrated care programmes are complex interventions: they consist of various interacting components, target individuals but also groups and organisations, have a variety of intended outcomes, are amendable to tailoring through adaptation and learning feedback loops, and their effectiveness is impacted by the behaviour of those delivering and receiving the intervention [8]. Common generic outcomes such as QALYs may not fully capture what these programmes are actually trying to achieve in persons with multi-morbidity. Their aims may go beyond the improvement of life expectancy and health-related quality of life, and include the improvement of well-being, the maintenance of independence, and increasing satisfaction with the care process.

In a time when the scarcity of resources is evident and evidence-based decisions on the spending of these resources are warranted, it is crucial to set up appropriate evaluations that can be used to convince decision-makers. Outcomes in the evaluations of complex care programmes often correspond at the higher level to the so-called 'Triple Aim': improving population health [and well-being], improving the patient's experience with care, and reducing cost(-growth) [9, 10]. These higher level outcomes, however, can be interpreted in different ways. Health can be defined as the absence of disease [11], or a wider perspective can be applied whereby health is seen as the complete physical, mental, and social well-being beyond merely the absence of disease [12]. More



For peer review only - http://bmjopen.bmj.com/site/about/guidelines.xhtml

recent definitions turn health into a more active term, as the ability to adapt [13] and as a ‘meta-capability’ that can be used to attain human value [14]. Similarly, experience with care can include many different aspects, such as the extent to which care is proactive or person-centred (e.g., two domains of the Patient Assessment of Chronic Illness Care (PACIC) instrument) [15]. Scientific literature found for a recent review also conducted in the context of the SELFIE research project, as the current study is (see Box 1), provided insight into which outcomes have been used in recent integrated care programme evaluations [5]. We saw that a wide array of indicators, corresponding to the Triple Aim, are being used. This is also reiterated by the findings in the review by Linton et al. (2016), whereby a total of 99 instruments to assess well-being were found, with a great variety between these [16].

Different perspectives and approaches can be taken when operationalising the Triple Aim and meaningful outcomes of integrated care programmes. In the current study we aim to better understand what good health or well-being and a good care process mean from the perspective of persons with multi-morbidity and to identify what they find most important in each. We hereby thus focus on two of the three ‘Triple Aims’. We look at this from a cross-country perspective by conducting focus groups in eight European countries involved in the SELFIE research project on integrated care in multi-morbidity: the Netherlands, Austria, Croatia, Germany, Hungary, Norway, Spain, and the UK (see Box 1). Using a qualitative focus group approach encourages interaction between persons to take place that may allow for novel concepts and themes to arise.

**<<<Insert here Box 1:** Information on the SELFIE project>>>

For peer review only



For peer review only - http://bmjopen.bmj.com/site/about/guidelines.xhtml

**Methods**

Focus groups were chosen because we were interested in the perspectives of persons with multi-morbidity themselves when it comes to health/well-being and care. The qualitative focus group methodology allows for novel concepts to arise, and interaction between persons can strengthen this process. The Consolidated criteria for reporting qualitative research (COREQ) checklist was used to structure this manuscript [17]. Eight focus groups were conducted with persons with multi-morbidity, one in each SELFIE partner-country: the Netherlands (Rotterdam), Austria (Vienna), Croatia (Zagreb), Germany (Berlin), Hungary (Budapest), Norway (Bergen), Spain (Barcelona), and the UK (Manchester). These countries have a long history of universal health care systems that are either tax-funded or funded through insurance premiums. However, they each have a unique health- and social care provider system in which persons with multi-morbidity might face different challenges. For more information on the organisation of care in each context, see the macro level descriptions of each country in the 'thick description' reports available on the SELFIE website (www.selfie2020.eu). The Dutch SELFIE team provided all other partners with a protocol on how to conduct and report on the focus groups, held a teleconference with each partner to discuss the protocol, and provided additional support throughout the process. All focus groups were organised between spring and fall of 2016.

**Recruitment & patient and public involvement**

The goal was to recruit 6-8 persons for each focus group, with a mix of gender, age distribution, and types of multi-morbidity ($\geq$2 health and/or social problems) being desired. Different recruitment strategies were applied: participants were recruited via patient organisations (AT, DE, HR, NL, NO), medical professional organisations (HR), self-help groups (AT, DE), medical centres (AT, ES, HU), non-profit care organisations (e.g., Red Cross) (AT), and patient and public involvement groups (UK). We specified that we were searching for persons with multiple health- and/or social problems. Participants were also recruited via SELFIE national Stakeholder Advisory Boards (SAB) (AT, DE, HU, NL, NO). Each country has a SAB that reflects on SELFIE findings and consists of five stakeholder groups (5P's): Patients (persons with multi-morbidity), Partners (informal caregivers), Professionals, Payers, and Policy makers. At the time that the focus groups were being organised, a SAB meeting had just taken place in each country, with two to four persons with multi-morbidity present. These persons were reached out to for the focus groups and we tried to snowball via their networks, e.g., also via patient organisations.

Participants were reimbursed for their travel costs and in some countries a gratuity was made available either as a token of appreciation or to aid recruitment (AT, DE, NL). As in most cases



For peer review only - http://bmjopen.bmj.com/site/about/guidelines.xhtml

no verbatim transcripts were made, these were not returned to participants. However, in some cases, participants were emailed after the meeting to thank them for their participation (DE, ES, HU, NL, NO, UK) and also in some cases were sent notes (NL). About one year after the focus groups, all participants were sent an update on the overall focus group results across countries. The current publication is also to be shared with participants.

**Procedure**

An extensive protocol was developed (see Supplementary file Box S.1) that was followed in each focus group. The first focus group was held by the Dutch team, who added 'lessons learned' to the protocol to aid the subsequent focus groups. Each country made a protocol in their own language to use during the actual focus group, which was held in their respective language. The focus groups were all held in a meeting room in an office, university, or clinical setting, with two to four researchers from the respective country present, one being the chair and at least one taking extensive notes throughout the meeting. At least one of these researchers is a co-author on this publication (FL, VS, MK, SCS, AZ, RE, CV, MiH, MB, MRM). The focus groups were also recorded. These researchers had a minimum of a bachelor's degree and experience/training in patient-contact and/or qualitative research.

The focus groups consisted of an introduction and two main parts: 1) defining a) good health and well-being, and b) a good care process, and 2) discussion on most important concepts and creating 'top-10' lists. The planned duration of the focus groups was two hours.

At the onset, the researchers welcomed everyone and introduced themselves, as only some researchers present had already been in touch with participants via telephone or email beforehand. The researchers stated their names, current position, and background expertise/occupation. Following this, in the introduction the researchers stated the purpose of the focus group: to discuss what is important from the perspective of an individual with multiple health- and/or social problems when it comes to health and care. The agenda of the focus group (introduction, part 1, part 2) was described. Next, the 'rules' of the focus group were introduced (see Supplementary File Box S.1) (e.g., respectful interaction, phones off). Participants signed the informed consent form (see Ethics statement below). The recorder was then turned on and participants were asked to introduce themselves and briefly describe their multi-morbidities.

In part one of the focus group, participants were asked to define good health/well-being and a good care process. First, a discussion about good health/well-being was held, followed by a discussion about a good care process. We started by asking participants to complete the sentence "For me, being in great health means…" and "I'd be really satisfied with all of the care / the overall



For peer review only - http://bmjopen.bmj.com/site/about/guidelines.xhtml

care that I receive, if…" Answers were discussed and written on flip-over boards. During this discussion a researcher asked triggering questions when needed and tried to focus the discussion on either health/well-being or care. After this, a researcher went through the statements on the flip-over board and the group tried to move from specific examples to general outcomes. These more general outcomes were highlighted / marked on the flip-overs and written on cards. Again a researcher needed to ask thought provoking questions: What do these original statements really boil down to? How can you generalise this so that it is applicable for others in the room? Throughout both steps a researcher also could pose the question as to how this is especially relevant or different for multi-morbidity.

After part one, there was a 15-30 minute break. During the break, the researchers arranged the cards with concepts, or outcomes, mentioned during part one (e.g., on tables or white/magnet boards). They also sorted through the a priori made cards from the literature and added those *not* mentioned during part one to the display. These cards were made a priori by the Dutch team, and distributed to each organiser. The outcomes on the a priori made cards stemmed from publications on integrated care programmes for multi-morbidity that were included in a large scientific literature review that was also conducted in the SELFIE project [5]. All outcomes included in those integrated care evaluation publications were ordered according to the Triple Aim and, where possible, overlap was removed. This resulted in a list of 77 potentially relevant outcomes (51 health/well-being, 22 experience, 4 cost) (see Supplementary File Box S.2). Each focus group organiser had been asked to translate these outcomes to their respective languages and write them on cards, using different colours for each Triple Aim. The purpose of including also these outcomes in the second part of the focus groups was to provide participants with a large and 'complete' (on the basis of the available literature) overview of possible outcomes.

In part two, the researchers briefly explained to the group that the cards from part one were now on display, and that they also added novel cards on the basis of findings in the literature. These new concepts were mentioned one-by-one and, where unclear, explained. Participants were asked to look at all the concepts. They were asked to write down the ten concepts that were most important to them on a sheet of paper. Hereafter, a discussion was opened as to what was on everyone's 'top-10' list.

**Analyses**

All focus groups were recorded and extensive notes were made, pictures of flip-overs and cards were made, and the top-10 lists were collected. In two cases, verbatim transcriptions were also made (HR, NO). Reports were made in English by each SELFIE partner on their respective focus



For peer review only - http://bmjopen.bmj.com/site/about/guidelines.xhtml

group, following a predefined template: structure of the focus group, recruitment, participant characteristics, reflection on the process, findings part one (concepts reflecting good health/well-being and a good care process), findings part two (summation of top-10 lists), and conclusion/discussion.

Three analytical steps can be distinguished. First, throughout the reporting and summarising done for each focus group, the researchers present at the focus group reflected on their respective focus group; at least one of these researchers is a co-author. Next, these reports were analysed independently by the first, second, and second-to-last author from the Dutch team. During this analysis step themes and priorities, corresponding to part one and two, were extracted from each focus group and these were compared across focus groups. Third, these themes and priorities were discussed amongst all co-authors, thus including at least one of author of each initial report who was present at the focus group. The analysis of part one was done applying an inductive (bottom-up) approach, whereby different specific concepts mentioned by participants were analysed by describing these, looking at their overlap and differences, and when possible clustering these into more general concepts. By deciding upfront that the procedure and our findings would be structured according to the Triple Aim, we also applied a deductive (top-down) approach. For part two, further clustering was done to present the findings in a clearer way, and a summation was used to identify concepts that were found most important. No specific qualitative analysis software was used in analysing the data. The goal from the outset was to conduct one focus group per country, thus data saturation was not discussed a priori.

**Ethics statement**

This study was conducted in line with the Declaration of Helsinki. According to the laws of most participating countries (Austria, Croatia, Germany, Hungary, the Netherlands, Norway) this type of study is exempt from formal ethical approval by a Medical Ethics Committee. In line with their national laws two countries (Spain and UK) obtained ethical approval. This is not a study in which participants were allocated to treatment groups or underwent any treatment; participants were not recruited as ‘patients’ but merely as persons living in the community with multi-morbidity. Participation was completely voluntary. All recordings, transcripts, and analyses were treated anonymously. All focus group participants signed an informed consent form; they received this form prior to their attendance to the focus group and at the start of the focus group had time to ask questions and sign the form. This form was developed by the Dutch team on the basis of the WHO template for informed consent for qualitative research [18] and translated by each country into their respective language. The informed consent consisted of information on the study, the purpose of



For peer review only - http://bmjopen.bmj.com/site/about/guidelines.xhtml

254 the research, type of research, participant selection, voluntary participation, procedure, duration, potential risks, benefits, reimbursements, confidentiality, sharing of the results, right to refuse / withdraw, and contact information.

For peer review only



For peer review only - http://bmjopen.bmj.com/site/about/guidelines.xhtml

## Results

### Participant and focus group characteristics

In total 58 persons participated across the eight focus groups. Only in some cases specific persons did not participate, this was because we could not get in touch with these persons, they had a conflicting agenda/schedule (e.g., holiday), were too busy, or in some cases had to cancel last minute due to illness or last minute (healthcare) appointments.

In all focus groups six or seven persons participated, except in Germany where 12 persons participated. During part two of the focus group in Germany, two subgroups were created to ease discussion. The mean age per focus group is presented in Table 1 below. The overall mean age was 65 years (range 31-86).

During the focus groups most frequently three researchers (min 2, max 4) were present, whereby one led [part of] the discussion and one took notes (total researchers present = 20, M/F = 4/16). The focus groups took 2.5 hours on average (min 2, max 3).

The majority of persons named three morbidities during the introduction of the focus groups (Table 1). Health problems were categorised according to the WHO 2010 International Classification of Diseases (ICD-10) [19]. For an overview of the specific morbidities mentioned by each participant at the start of the focus groups see Table 2. Across countries, diseases of the circulatory system, musculoskeletal system, and endocrine disorders were most common morbidities amongst participants (Table 2). Specific examples of frequently mentioned diseases were high blood pressure, rheumatism, arthritis, and diabetes mellitus. It is of note that only in the UK mental health problems were explicitly named at the start of the focus groups (Table 2).

<<<**Insert here Table 1:** Focus group participant characteristics>>>

<<<**Insert here Table 2:** Morbidities of participants per country categorised according to the ICD-10>>

### Defining health and care (part 1)

*Defining health*

Across all focus groups the idea of good health being the ability to do **'normal' daily activities** was mentioned. This for example included activities such as going outside and undertaking activities (ES), being able to use a computer (UK), and doing physical activities within realistic reach:



For peer review only - http://bmjopen.bmj.com/site/about/guidelines.xhtml

"…Also being active is necessary for well-being. I was always very athletic nowadays only limited, but it works if you adapt your activities to your physical ability." (P2; AT)

*Response*: "That's true, though I cannot climb mountains anymore, but I can walk through different parks and I can still use the stairways." (P7; AT)

In three focus groups it was also said that especially in the case of multi-morbidity it is not only about doing such daily activities, but being able **to plan** them and **structure** them yourself (DE, NO, UK). This is related to having realistic expectations about what one can do and relates to acceptance. **Acceptance** was mentioned in terms of self-acceptance and acceptance by others (AT, DE, HU, NL). For example:

"Part of it is acceptance, I can do what I can do, and I should leave the other things." (P4; NL)

"How to handle the pain and the disease plays an important role. Integration of your diseases in your daily activities, accepting the pain and especially not feeling bad all the time although the diseases are permanent is a big step." (P11; DE)

"For me, I even feel good, if no additional things come. In my case, all of my conditions and problems that I was dealing with are considered as end-stage or final stage, and this is accepted. Therefore if my condition is not worsening, then I am fine." (P5, HU)

Daily activities are also linked to the desire to maintain **social relationships** and **participation** in society (AT, DE, ES, HR, HU, NL, NO).

"I want to be consciously active in society. With everything, doing what I want to do, being useful for others." (P7; NL)

"For me, good health means being psychosocially active…" (P1; HR) (i.e., having social relationships that are meaningful, being an active participant in everyday life)

In many countries good health was also associated with the **absence of shame, discrimination, fear, and/or stigma** (ES, HR, HU, NL, NO, UK). These feelings could relate to the wider public, the patient-provider, or the personal realm. For example, in Spain the examples of fear of walking alone and



For peer review only - http://bmjopen.bmj.com/site/about/guidelines.xhtml

being vulnerable or shame of being seen with medical apparatuses such as an oxygen machine were mentioned. In Hungary shame was mentioned in the patient-provider relation, e.g., not being able to keep to a diet. In Croatia these concepts were summarised as personal vulnerability. Fear of the future and not knowing how the disease trajectory will go was also discussed (HU, NL, NO). Doubts and worries about sharing updates on one's diagnoses with family and the impact and burden that might have was mentioned (AT). In the Spanish focus group the discussion on fear and shame was heated, as persons dealt and coped with this issue differently. This relates to a point that also came up, of not only accepting the health problems, but also **coping**, being resilient, managing, and having responsibility for the diseases themselves, which may be perceived to be especially difficult in the case of multi-morbidity (DE, HU, NL, NO, UK).

> "Oh yeah, I think if you have multiple [diseases] it just adds to the whole workload really, how to cope with different things, oh yeah." (P1; UK)

The general idea of good health being defined as **feeling safe** also was discussed in various focus groups (ES, HU, NL, NO, UK). This can be seen as the result of being absent from the aforementioned negative states (e.g., fear). Feeling safe was also discussed in terms of trusting professionals (discussed more below). In Norway, feeling safe also extended to the economic realm:

> "…fear, fear for one day having to give up your work for example … if I don't manage any more, if I am unable to work anymore, then we do not have the economic resources to live here anymore, this was in my thoughts when the illness hits me and then fear…" (P2; NO).

Lastly, during the open discussion in part one, participants mentioned having a positive frame of mind, being able to enjoy life, and the importance of **psychological well-being** was mentioned in all focus groups.

> "I think psychological problems ought to be mentioned. Many people with chronic illnesses are also struggling psychologically. It could be because you have bad conscience, because you are dreading something, because you do not know if you will manage and suffer from performance anxiety". (P1; NO)

> "I want to enjoy my life, even though I've these diseases. That means, just being full of life." (P4; AT)



For peer review only - http://bmjopen.bmj.com/site/about/guidelines.xhtml

*Defining care*

When considering the wide array of themes discussed when defining health/well-being, it is not surprising that good care was defined by many persons as being approached as a whole person and **being treated holistically** (AT, NO, UK).

> "I wish that people treat me in a respectful manner, because it's true, I am sick, but the disease is not me. I don't want to be reduced to my diseases." (P2; AT)

> "So I would have wished for a doctor that, to put it this way, had the overview of the whole human being, that he shouldn't treat a heart disease just in isolation, you have another disease, and a third..." (P2; NO)

Persons explained that being approached holistically also means receiving **holistic support**, including informal caregiver support (HR, NL, NO, UK), good information provision, and especially **emotional and psychological support.** Participants mentioned that support should take the form of more extensive, easily accessible, and timely information on the health problems at hand and medication, but also psychological support, support with self-management, and self-help groups for example (AT, DE, ES, HR, HU, NL). Concerning emotional and psychological support specifically, this is needed even when this is not the 'main' problem at hand (AT, ES, HR, NL, UK). For example, in the Netherlands participants mentioned that in the case of multi-morbidity it takes time to accept the new health problems that arise on top of existing health problems and that there should be support for this adjustment period. In the UK a disconnection between the patient- and provider's priorities was seen, as it was mentioned that the healthcare professionals do not focus on the mental health issues, when they should:

> "Yeah, for me I'd like the mental health to be bigger…for me all my healthcare professionals see the [physical disease] as the big thing with me, I don't, I see my depression as the big thing because that's what affects me day to day." (P4; UK)

> "What I see is that there's no psychological aid. When you are told you are a chronic patient and you have to take a drug all your life, and that this is for all your life, some people are depressed, needs psychological aid…" (P1; ES)



For peer review only - http://bmjopen.bmj.com/site/about/guidelines.xhtml

A wider theme discussed was **trust in professionals and the system**. This is in part linked to the emotional and psychological support, whereby participants felt that two-way trust is needed in the relationship between the patient and professional so that psychological issues can be discussed, the professional really listens, and so that they can embark on the care process together (AT, NL, NO, UK). Related to this, respectful interaction between professional and patient was also often mentioned (AT, DE, NL, NO, UK, HU). Specifically in the UK, the direction of trust was mentioned with participants saying that they needed to be 'believed' by the professionals (P2, P4, UK).

Trust also pertained to being able to rely on the professional in being able to help, based on their skills and knowledge (DE, ES, HR, NL, NO, UK). Participants spoke of having a 'prepared' care professional to talk to, and trusting their expertise and education.

> "Yeah, but there are difficulties with medication when you've got multiple things..." (P5; UK)
> *Response*: "Oh yeah, but you'd expect your GPs to keep on top of that, it is debatable as to whether that actually happens or not." (P1; UK)

In Hungary, Germany, and the Netherlands the trust also pertained to the professionals in general:

> "Trust in the medical world, well I'm sceptical about that, a lot of things happen around you, that you think, did they not see that?... Yeah, then you lose faith/trust because of the things that happen to you…" (P2; NL)

> "If I visit my doctor, I wish I could reproduce what he is doing there, which services are provided and I would like to sign for them. I want to be more informed about what is done. This way, I often cannot trust my physicians." (P8; DE)

As may be especially relevant in multi-morbidity, issues surrounding **continuity of care** were mentioned in all focus groups. This pertained to clear responsibilities, a clear contact point, transfer and 'after care', good communication, and good collaboration and teamwork. These points about continuity often also related to **sharing information or medical records** between providers and organisations (DE, ES, HR, HU, NO, NL).

> "The problem is the coordination between the primary health centres with the reference hospitals. The doctor at the hospital should see all the information, and the family doctor as well…" (P5; ES)



For peer review only - http://bmjopen.bmj.com/site/about/guidelines.xhtml

"I would expect that all the institutions and all the GPs in this city would be connected to the same electronical [IT] system. In this case I would not need to go with all those stacks of papers when I see a doctor. (...) There should be a system which can be seen by everyone and not only by some particular segments of the care." (P1, HU)

**Cross-country comparisons in defining good health and care (part 1)**

Little variation was seen between countries when defining health (part 1). Themes surrounding the definition of a good care process differed more between countries. Although aspects of access and availability were mentioned in all countries, this concept took a different form between the countries. In some cases this pertained to timely care in terms of waiting lists and enough time (ES, HU, NL). In Hungary, however, this specifically had to do with the lack of information on the waiting times. Persons emphasized that waiting times were more acceptable if they knew in advance how long they had to wait.

"The worst thing is waiting… They could calculate an order with some gap in between. There are problems with the information sharing" (P6; HU)

Access in the form of time also referred to professionals having enough time for persons with multi-morbidity in Germany, Hungary, and Norway.

Availability also had different definitions per country – in Croatia, Germany, and the UK this was treatment, care, and provider availability and the freedom to choose between them. In Norway, Spain, and the UK this was also geographical- availability and access.

Several concepts were only brought up in part one by certain countries. Namely, bureaucracy or the reduction of the burden thereof was mentioned in Germany and the Netherlands, for example with regard to care services that fall within our outside of the insurance package.

"There are persons who are physically not able to walk to their physiotherapist anymore. In fact they are in need of transport, but you have to apply for transportation for every single therapeutic unit. These are very difficult circumstances due to too much bureaucracy. This is disastrous…" (P9; DE)

In Austria participants discussed the need for more self-help and support groups. In contrast, in the Netherlands this was mentioned as a positive aspect already present and available. Participants in



For peer review only - http://bmjopen.bmj.com/site/about/guidelines.xhtml

Austria were aware that these things exist in other countries, and felt it was missing in their own context.

Lastly, physical surroundings in care provision were mentioned in Hungary that were related to cleanliness, enough personal space, and enough seats.

> “Because if we consider our life or our homes, we do not like if it is dirty and messy. In a good facility there should be cleanliness, order and discipline.” (P2; HU)

> “At the … [department] patients need to wait in a very narrow corridor with one row of chairs. There are about three times more patients than chairs. (P5; HU)

Furthermore, in Hungary systematically organised operating procedures in care was an important requirement mentioned by multiple participants. The lack of organised procedures is exemplified by the fact that the care process in hospitals is sometimes unreasonably long, the staff is rushed, there are no clear instructions on the next steps of the procedure, there are redundant diagnostic tests, and information is not shared appropriately among professionals.

**Most important concepts (part 2)**

In all focus groups, in part 2, participants were asked to make a top-10 list of most important concepts. These concepts were those identified in part 1 and supplemented with ‘missing’ concepts from the literature (see Supplementary File Box S.2). These top-10 lists were discussed. There was large variation within focus groups (i.e., countries) as to what concepts were deemed most important and on participants’ top-10 lists. In Supplementary File Box S.3 all concepts written on persons’ top-10 lists are presented, including those from the literature and ‘novel’ concepts derived during part 1 of the focus groups.

In Table 3 below an overview of the most frequently mentioned concepts is presented. These are concepts written on the top-10 lists of at least 10 persons (out of the 58 participants in total) across all countries. The following health and well-being concepts were most frequently noted by participants on their top-10 lists: social relationships, a positive frame of mind or resilience, enjoyment of life, and maintaining independence. A positive frame of mind or resilience, was mentioned by at least one person in each country’s focus group. Many facets pertaining to good interactions between care professionals and persons with multi-morbidity were on the top-10 lists of participants, such as good communication, shared decision-making, and respect. Furthermore, individualised care planning and a holistic assessment and understanding of the problems at hand



For peer review only - http://bmjopen.bmj.com/site/about/guidelines.xhtml

were aspects of the care process also frequently on the top-10 lists. Lastly, pro-active, prevention-oriented, care was found important by many participants. As can be seen, there is great variety in concepts that participants put on their top-10 lists, both within and across countries. Furthermore, some phrases and words are quite specific whereas others are broad; participants were free to determine at what conceptual level they wrote their top-10 outcomes.

**<<<Insert here Table 3:** Most frequently listed outcomes on the ‘top-10’ lists of focus group participants across the eight focus groups>>>

For peer review only



For peer review only - http://bmjopen.bmj.com/site/about/guidelines.xhtml

## Discussion

### Main findings

Participants defined good health and well-being in terms of being able to conduct 'normal' daily activities, being able to plan and structure these, and having social relationships and participating in society. Acceptance by oneself and by others and coping with one's current health situation were deemed aspects of good health. Absence of shame, discrimination, fear, and/or stigma from the public, care professionals and oneself, and on the other end feeling safe and psychological well-being were also facets of good health. Social relationships, resilience, enjoyment of life, and maintaining independence were considered the *most* important aspects of good health across participants from all countries. A good care process was defined as one whereby persons are approached and supported holistically, with specific attention for emotional and psychological support, there is confidence and trust in professionals and the system, continuity of care is guaranteed, and where information is shared and accessible within a reasonable time. Concepts deemed *most* important were good communication, shared decision-making, and respect between care professional and the person with multi-morbidity, as well as individualised care planning and pro-active, prevention-oriented, care. Little cross-country variation in health themes were found, however, in defining care differences did exist, for example in terms of the exact type of access referred to (e.g., geographical distance to care providers, timely access).

### Interpreting findings

Several themes brought up during the focus groups were explicitly mentioned to be more relevant for persons with multi-morbidity; this was especially the case when defining care. One such concept was enough time: in Norway persons mentioned that it is difficult when their issues are not immediately visible for a care professional and that they need time to explain the multiple problems at hand. Also confidence in professionals' skills are related to their needing to address or at least be aware of multiple problems and often multiple medications, some of which may go beyond their specialist area of expertise. Considering the different combinations of morbidities represented in the focus groups and the fact that participants are living in different health- and social care contexts, it is interesting to note that regardless of whether the main care professional came from primary, secondary, or social care, these aspects relating to respect, providing emotional support, and expertise were important for many participants. This might be central to the fact that persons with multi-morbidity deal with multiple care professionals who need to have attention for the person as a whole, considering all their morbidities and the emotional burden that comes alongside these. In line with this, well-coordinated and smooth transitions (i.e., continuity of care) are particularly



For peer review only - http://bmjopen.bmj.com/site/about/guidelines.xhtml

important for persons with multi-morbidity, who often cross provider-, organisation- and sector boundaries throughout their care trajectories. In the realm of continuity of care, in many focus groups sharing information for example via shared electronic medical records was explicitly mentioned. It is of note that issues surrounding privacy were not mentioned by participants, although EMRs are in most cases not in place yet due to such issues at a wider system level.

Aspects especially to do with defining care seemed relatively unique and extra relevant for multi-morbidity. However, there was a large degree of overlap in the concepts mentioned in defining health in the current focus groups and the existing definitions presented in the introduction by the WHO, Huber and Vankatapuram, and similar studies conducted on defining health [12-14]. Namely, absence of shame, discrimination, fear, and/or stigma, feeling safe, psychological well-being, and social relationships and participation especially overlap with the mental and social well-being aspects of the 1946 WHO definition of health [12]. The importance of mental health outcomes has also been recently found in a study amongst a UK general population on what aspects of the EuroQol 5-dimensions (EQ-5D) are currently missing or receive too little attention, i.e., mental health [20]. This is also in line with a study by Ebrahimi and colleagues (2012) amongst frail elderly where being able to 'master' daily life (e.g., coping and acceptance) and being happy and satisfied with life (e.g., enjoyment of life) were described as characteristics of health [21].

Other concepts found in the current focus groups relate more to the definition by Huber and colleagues of health as the ability to adapt, i.e., acceptance, coping, resilience [13]. Also overlap was seen with our finding on the ability to conduct normal daily activities and the aforementioned study on frail elderly where the ability to live the routine life persons are accustomed to was identified as an aspect of health [21]. Lastly, especially Venkatapuram's definition of health as the capability to attain human value, seems to overlap with the general idea of the ability to participate in normal daily activities and social participation (a means to attain human value) and enjoyment of life as mentioned throughout the current focus groups [14].

The facets of good health found in the current study for a large part overlap with facets of functioning as set out by the International Classification of Functioning (ICF) [22]. The goal of the ICF is to describe a person's functioning, irrespective of their specific health conditions, considering their context. The ICF can be used to classify aspects of functioning that can be measured when assessing the impact of, for example, integrated care programmes. However, in the current study we aimed to identify aspects of 1) health and wellbeing, and 2) experience with care, that can be used when evaluating the impact of integrated care programmes on the Triple Aim. Our findings are reaffirmed by the fact that the different themes and concepts that persons in the focus groups used to define good health can be retraced to the ICF.


For peer review only - http://bmjopen.bmj.com/site/about/guidelines.xhtml

It was noticeable that, across all focus groups, aspects relating to care were already mentioned in defining good health. It appeared that persons could not always clearly distinguish the two. This might be due to the fact that persons more easily can identify these care-related factors as 'changeable'. When exploring which aspects of health/well-being and care were most important in part two of the focus groups, we saw across countries that these overlapped with those mentioned during the first part of the discussions but also that often concepts were included from the literature-cards. As these were introduced and explained after the break, participants reacted positively to them. This indicates conceptual overlap in what is currently being assessed in evaluations, and thus found in the literature a priori, and what participants of our focus groups found important [5].

**Strengths & limitations**

Several strengths and potential limitations in the current study are described below. Strengths include the use of a broad set of inductively- and deductively deduced outcomes, the variation between the countries in which the focus groups were held, the fact that persons with multi-morbidity were so actively involved in this research, and that the focus was not only on health but also on the care process. Potential limitations may be the cross-country differences in discussing mental health problems, the time restriction that slightly changed the planned structure of the focus groups, and the variation in specificity between concepts.

A difference in explicitly naming mental health problems between countries was observed. Culturally-related stigma issues may play a role. In the UK mental health problems were explicitly mentioned by participants when they listed their morbidities. This was not the case in the Netherlands, although throughout the focus group it became apparent that depression issues were also present amongst participants. Also in the German focus groups mental health problems, such as depression, were mentioned as 'side effects' of other health problems. It is possible that our findings would have differed had more persons with (diagnosed) psychological multi-morbidities been present at the focus groups. It could be that different aspects related to health and care would have been pointed out, for example because in many health- and social care systems of the included eight countries there is fragmentation between psychological- and somatic health care provisions. It is possible that certain findings would have been strengthened, such as the importance of being approached holistically and that somatic care professionals pay attention to psychological health problems and provide emotional support. Although having a more varied group of participants in this sense would be desirable, it is not a requirement nor a goal in qualitative studies to attain a representative sample.



For peer review only - http://bmjopen.bmj.com/site/about/guidelines.xhtml

For peer review only

A strength of the current study is that inductively and deductively derived outcomes were used in the second part of the focus groups. We chose to do so in order to provide participants with a broad set of possible outcomes to choose from. The added outcomes from the literature were, alongside novel concepts from part one of the focus groups, on participants' 'top-10' lists (see Supplementary File Box S.1). This indicates that simply because an outcome was not mentioned inductively in part one of the focus group, this did not mean that participants did not find it important.

Initially, the goal of the second part of the focus groups was to reach a group 'top-10', in which participants would discuss their individual lists and try to create a group list. However, due to time restrictions this was not possible. Thus the results of part two reflect each individual's opinion. We opted to present findings in the results section mentioned by ten or more persons across the focus groups, but this is a relatively arbitrary cut-off point, and the full findings are shown in the Supplementary File Box S.3. The total number of cards from which participants could select concepts for their top-10 list differed in size depending on from which country they are from. The level of specificity in concepts also differs greatly, for example the overall satisfaction with care process is mentioned, as well as the specific facet that a provider has enough time to listen to a patient (see Supplementary File Box S.3). Quantifying such a qualitative process is thus to be done cautiously. In the Supplementary File Box S.3 concepts are clustered into broader categories. There are, however, different categorisations possible, since the concepts are all interrelated and different definitions can be applied.

We consider it a strength of the current research that the participating countries in SELFIE differ in geographical size, population [density], culture, and welfare and health care systems – providing a wide scope. Furthermore, it is a main strength that persons with multi-morbidity were so actively approached and included in the current study and in the wider SELFIE study, introduced in Box 1. As will be described below, the results of these focus groups form the predominant input for the core set of outcomes to be included in the SELFIE evaluations of 17 integrated care programmes: participants were given a direct voice and greatly valued this. Another main strength of the current study and great addition to the literature is the fact that we asked participants in our focus groups to not only reflect on health, but also on experience with care. In situations whereby health improvements, especially in the traditional physical and clinical health indicators, are not feasible, focusing on satisfactory and even positive care process experiences becomes more important. And, as was seen in the current study, experience with care is often inextricably linked to the definition of health. Although we did not explicitly incorporate costs in the first part of the focus groups when discussing what outcomes are important, participants did mention several cost concepts such as

For peer review only



For peer review only - http://bmjopen.bmj.com/site/about/guidelines.xhtml

commuting costs and out-of-pocket fees. In the future, it would be interesting to also have an explicit discussion on what role costs play in, especially, the choice of and experiences with care.

**Next steps**

When evaluating integrated care programmes for multi-morbidity, a wide array of health- and care outcomes should be included. Few aspects related to physical functioning, a traditionally used outcome, were found on the top-10 lists of importance, i.e., only 'energy and fatigue' was mentioned by more than 10 persons across all focus groups. Instead, more well-being, social, and psychological facets of health are important and should thus be included in evaluations. This finding overlaps with current trends in conducting more comprehensive evaluations. To this end, in SELFIE a core set of outcomes for the evaluation of 17 promising integrated care programmes for multi-morbidity was developed that places a lot of focus on such outcomes. The core set consists of the following outcomes: physical functioning, psychological well-being, social relationships & participation, enjoyment of life, resilience, person-centeredness, continuity of care, and total health- and social care costs. This core set was developed on the basis of four sources, of which the focus groups described in the current article had the most prominent influence. The other three sources were: i) findings from a literature review conducted to develop a framework on integrated care for multi-morbidity and to identify existing programme evaluations [5], ii) (inter)national stakeholder advisory board discussions in the eight SELFIE partner countries with Patients, Partners, Professionals, Payers, and Policy makers, and iii) the aims of the aforementioned 17 programmes and current indicators included in these programmes. A set of criteria was used to come to this list (see Supplementary File Box S.4), and some concepts mentioned in the focus group were not included in the core set, because these did not meet these criteria. This is the case for the more system- and cultural-wide concepts which cannot be used to assess the performance of one specific care programme, such as for example the absence of public stigma.

**Conclusion**

In the current study persons with multi-morbidity in eight European countries described what good health- and care means to them. Beyond traditional outcomes, aspects such as acceptance, absence of shame, and enjoyment of life came forth from the discussions. Especially important for persons with multi-morbidity was to be approached holistically and to have continuity of care. Thus a variety of health outcomes that entail well-being, social, and psychological facets should be included when evaluating integrated care programmes for persons with multi-morbidity. Even more imperative is that experience with care outcomes be included in such evaluations that tackle the complexity of



For peer review only - http://bmjopen.bmj.com/site/about/guidelines.xhtml

multi-morbidity care provision. In conclusion, important next steps include moving towards harmonising evaluation frameworks and the specific indicators used in such evaluations.

For peer review only



For peer review only - http://bmjopen.bmj.com/site/about/guidelines.xhtml

**Funding statement**

The SELFIE project has received funding from the European Union's Horizon 2020 research and innovation programme under grant agreement No 634288. The content of this publication reflects only the SELFIE groups' views and the European Commission is not liable for any use that may be made of the information contained herein.

**Acknowledgements**

We would like to thank the focus group participants for making this research possible. We would also like to thank the entire SELFIE consortium for their support.

**Data sharing statement**

The recordings and available verbatim transcripts will not be made available, to protect the anonymity of participants. Upon request to the corresponding author, we may consider in collaboration with the respective SELFIE-partner, whether a country-specific focus group report can be shared. Other findings from the overarching SELFIE study can be found on the SELFIE website (www.selfie2020.eu).

**Competing interests**

None of the involved co-authors had a competing interest.

**Author's contribution**

FL, MB, and MRM drafted the focus group procedure document and analysis instruction document, and organised and were present at the Dutch focus group. VS, MK, SCS, AZ, RE, CV, MiH, and TC were involved in the translation and organisation of the focus groups in their respective countries and in the analysis of their focus group and reporting thereof. FL, MB, and MRM analysed the Dutch group; FL, MaH, MB, and MRM analysed all focus group reports and conducted the overarching analyses. FL, MaH, and MRM drafted the manuscript and the revision; all co-authors critically reviewed and contributed to the manuscript and revision, e.g., by appraising themes and providing fitting quotes.

For peer review only

**References**


1. van Oostrum SH, Picavet HSJ, van Gelder BM, et al. Multimorbidity and comorbidity in the Dutch population – data from general practices. BMC Public Health. 2012;12:715.
2. Barnett K, Mercer SW, Norbury M, et al. Epidemiology of multimorbidity and implications for health care, research, and medical education: a cross-sectional study. Lancet. 2012;380(9836):37-43.
3. Mair FS & May CR. Thinking about the burden of treatment. BMJ. 2014;348:g6680.
4. van der Heide I, Snoeijs S, Melchiorre MG, et al. Innovating care for people with multiple chronic conditions in Europe: An overview. Utrecht, the Netherlands: ICARE4EU, NIVEL, 2015.
5. Struckmann V, Leijten FRM, van Ginneken E, et al. Relevant models and elements of integrated care for multi-morbidity: results of a scoping review. Health Policy. 2018;122:23-35.
6. de Bruin SR, Versnel N, Lemmens LC, et al. Comprehensive care programs for patients with multiple chronic conditions: A systematic review. Health Policy. 2012;107(2-3):108-145.
7. Hopman P, de Bruin SR, Forjaz MJ, et al. Effectiveness of comprehensive care programs for patients with multiple chronic conditions or frailty: a systematic literature review. Health Policy. 2016;120:818-832.
8. Craig P, Dieppe P, Macintyre S, et al. Developing and evaluating complex interventions: the new Medical Research Council guidance. BMJ. 2008;337:a1655.
9. Berwick DM, Nolan TW, Whittington J. The Triple Aim: Care, health, and cost. Health Aff. 2008;27(3):759-769.
10. Whittington JW, Nolan K, Lewis N, et al. Pursing the Triple Aim: The first 7-years. Milbank Q. 2015;93(2):263-300.
11. Boorse C. Health as a theoretical concept. Philosophy of Science. 1977;44(4):542-573.
12. World Health Organization. WHO Constitution. 1946.
13. Huber M, Knottnerus JA, Green L, et al. How should we define health? BMJ. 2011;343:d4163.
14. Venkatapuram, S. Health Justice. Cambridge: Polity Press, 2011.
15. Glasgow RE, Wagner EH, Schaefer J, et al. Development and validation of the Patient Assessment of Chronic Illness Care (PACIC). Med Care. 2005;43:436-444.
16. Linton M, Dieppe P, Medina-Lara A. Review of 99 self-report measures for assessing well-being in adults: exploring dimensions of well-being and developments over time. BMJ Open. 2016;6:e010641.




For peer review only - http://bmjopen.bmj.com/site/about/guidelines.xhtml

17. Tong A, Sainsbury P, Craig J. Consolidated criteria for reporting qualitative research (COREQ): a 32-item checklist for interviews and focus groups. Int J for Qual Health Care. 2007;19:349-357.

18. World Health Organization. Informed Consent Form Template for Qualitative Studies. <http://www.who.int/rpc/research_ethics/informed_consent/en/>

19. World Health Organization (WHO). International Classification of Diseases (ICD-10). Geneva: WHO; 2010.

20. Shah KK, Mulhern B, Longworth L, Janssen MF. Views of the UK general public on important aspects of health not captured by EQ-5D. Patient. Online first, 2017. DOI: 10.1007/s40271-017-0240-1

21. Ebrahimi Z, Wilhelmson K, Moore CD, et al. Frail elders' experiences with and perceptions of health. Qual Health Res. 2012;22:1513-1523.

22. World Health Organization (WHO). International Classification of Functioning (ICF). Geneva: WHO; 2001.

For peer review only



For peer review only - http://bmjopen.bmj.com/site/about/guidelines.xhtml

## Tables & Figures

**Box 1:** Information on the SELFIE project

**SELFIE** (Sustainable intEgrated chronic care modeLs for multi-morbidity: delivery, FInancing, and performancE) is a Horizon2020 funded EU project that aims to contribute to the improvement of person-centred care for persons with multi-morbidity by proposing evidence-based, economically sustainable, integrated care programmes that stimulate cooperation across health and social care and are supported by appropriate financing and payment schemes. More specifically, SELFIE aims to:

- Develop a taxonomy of promising integrated care programmes for persons with multi-morbidity;
- Provide evidence-based advice on matching financing/payment schemes with adequate incentives to implement integrated care;
- Provide empirical evidence of the impact of promising integrated care on a wide range of outcomes using Multi-Criteria Decision Analysis (MCDA);
- Develop implementation and change strategies tailored to different care settings and contexts in Europe, especially Central and Eastern Europe.

The SELFIE consortium includes eight organisations in the following countries: the Netherlands (coordinator) (NL), Austria (AT), Croatia (HR), Germany (DE), Hungary (HU), Norway (NO), Spain (ES), and the UK. Each country has a unique health- and social care system, that varies in the extent to which health- and social care are integrated and that vary in how the financing is arranged. On the SELFIE website (www.selfie2020.eu) a macro level description of the systems can be found that provides background context.

Seventeen promising integrated care programmes for persons with multi-morbidity across these eight countries are being evaluated in SELFIE. The evaluations apply MCDA and each use a common set of core outcomes as well as programme-type specific outcomes. The latter depend on whether a programme is i) a population health management programme, ii) a programme targeting frail elderly, iii) a programme targeting persons with problems in multiple life domains, or iv) an oncology or palliative care programme.

[Grant Agreement No 634288]

**Table 1:** Focus group participant characteristics

| | **# (M/F)** | **Mean age (Min-Max)** | **Mean number of morbidities** |
|---|---|---|---|
| **Austria (AT)** | 7 (5/2) | 72.9 (62-84) | 2.3 |
| **Croatia (HR)** | 7 (4/3) | 51.7 (31-69) | 3 |
| **Germany (DE)** | 12 (4/8) | 62.4 (37-78) | 3.9 |
| **Hungary (HU)** | 6 (1/5) | 64.5 (47-78) | 3 |
| **Netherlands (NL)** | 7 (5/2) | 66.3 (53-75) | 5 |
| **Norway (NO)** | 7 (2/5) | 65.4 (42-76) | 3.1 |
| **Spain (ES)** | 6 (5/1) | 70.2 (60-81) | 2.7 |
| **United Kingdom (UK)** | 6 (4/2) | 68.8 (58-86) | 4 |
| **Total** | 58 (30/28) | 65.3 (37-86) | 3.4 |



For peer review only - http://bmjopen.bmj.com/site/about/guidelines.xhtml

**Table 2:** Morbidities of participants per country categorised according to the ICD-10

| | **Total** | **Austria** | **Croatia** | **Germany** | **Hungary** | **Netherlands** | **Norway** | **Spain** | **UK** |
|---|---|---|---|---|---|---|---|---|---|
| **Neoplasms** | 8 | 3 | 1 | 2 | | 1 | | 1 | |
| Diseases of the **blood** and disorders involving the immune mechanism | 3 | | 2 | | | | 1 | | |
| **Endocrine**, nutritional, and metabolic disease | 31 | 2 | 1 | 6 | 5 | 5 | 4 | 3 | 5 |
| **Mental** and **behavioural** disorders | 6 | | | | | | | | 6 |
| Diseases of the **nervous** system | 20 | 1 | 2 | 12 | 2 | | 1 | | 2 |
| Diseases of the **eye** and adnexa | 7 | | | | 1 | 6 | | | |
| Diseases of the **ear** and mastoid process | 5 | | | 3 | | | 1 | | 1 |
| Diseases of the **circulatory** system | 44 | 1 | 5 | 12 | 8 | 7 | 4 | 4 | 3 |
| Diseases of the **respiratory** system | 17 | | 1 | | | 6 | 4 | 6 | |
| Diseases of the **digestive** system | 10 | 1 | 1 | 1 | 1 | 2 | 1 | 1 | 2 |
| Diseases of the **skin** and subcutaneous tissue | 3 | 1 | 1 | | | | | | 1 |
| Diseases of the **musculoskeletal system** and connective tissue | 36 | 6 | 6 | 9 | 1 | 4 | 5 | 1 | 4 |
| Diseases of the **genitourinary** system | 4 | | | | | 4 | | | |
| **Congenital malformations**, **chromosomal abnormalities** | 1 | 1 | | | | | | | |
| Symptoms, signs, **abnormal clinical and lab findings**, **not else classified** | 2 | | | 2 | | | | | |
| **Injury**, **poisoning** and other consequences of external causes | 1 | | | | | | 1 | | |

Note: ICD-10: International Statistical Classification of Diseases and Related Health Problems, 10th revision.

For peer review only



For peer review only - http://bmjopen.bmj.com/site/about/guidelines.xhtml

**Table 3:** Most frequently listed outcomes on the 'top-10' lists of focus group participants across the eight focus groups

| | **# / 58** | **Austria (AT)** | **Croatia (HR)** | **Germany[1] (DE)** | **Hungary (HU)** | **Netherlands (NL)** | **Norway (NO)** | **Spain (ES)** | **United Kingdom (UK)** |
|---|---|---|---|---|---|---|---|---|---|
| *Health and well-being* | | | | | | | | | |
| Energy & fatigue | **12** | 4/7 | | | 1/6 | 4/7 | 2/7 | | 1/6 |
| Feeling safe | **10** | | 1/7 | | 3/6 | 3/7 | | 3/6 | |
| Cognitive functioning | **12** | 4/7 | | 1/6 | | 2/7 | 1/7 | 3/6 | 1/6 |
| Maintaining independence | **16** | | 2/7 | 5/6 | 1/6 | 1/7 | | 1/6 | 1/6 |
| Enjoyment of life | **16** | 2/7 | | 4/6 | 1/6 | 3/7 | 3/7 | | 3/6 |
| Positive frame of mind, resilience | **16** | 2/7 | 3/7 | 2/6 | 2/6 | 1/7 | 2/7 | 2/6 | 2/6 |
| Self-esteem | **11** | 1/7 | 1/7 | 3/6 | | 3/7 | 2/7 | | 1/6 |
| Social relationships | **17** | 3/7 | | 5/6 | 2/6 | 4/7 | | 2/6 | 1/6 |
| Societal participation | **12** | 1/7 | 3/7 | 3/6 | | 3/7 | | 1/6 | 1/6 |
| *Experience* | | | | | | | | | |
| Individualised care planning / tailored care | **13** | 1/7 | | 4/6 | 1/6 | 2/7 | 4/7 | | 1/6 |
| Holistic assessment/understanding | **11** | 1/7 | 1/7 | 4/6 | 2/6 | 2/7 | | | 1/6 |
| Good communication between provider-patient | **14** | 1/7 | 1/7 | 2/6 | | 2/7 | 1/7 | 3/6 | 4/6 |
| Shared decision-making professional-patient | **13** | 2/7 | | 4/6 | 1/6 | 1/7 | 1/7 | | 4/6 |
| Respectful interaction between professional-patient | **12** | 2/7 | 1/7 | 5/6 | | | | | 4/6 |
| Shared information between professionals | **10** | 2/7 | 1/7 | 1/6 | 2/6 | 2/7 | | 1/6 | 1/6 |
| Team work between professionals & providers | **10** | | 1/7 | | 2/6 | | 2/7 | 2/6 | 3/6 |
| Confidence in knowledge and skills in professionals | **10** | 1/7 | 2/7 | 3/6 | 2/6 | 1/7 | | 1/6 | |
| Pro-active, prevention-oriented, care | **13** | | 1/7 | 1/6 | 4/6 | 4/7 | | | 3/6 |

Note: 1 = Group split into 2, so each time # out of 6 persons, each group discussed either health/well-being and costs or care and costs.



For peer review only - http://bmjopen.bmj.com/site/about/guidelines.xhtml

**Supplementary files** accompanying the manuscript:

Defining good health and care from the perspective of persons with multi-morbidity: Results from a qualitative study of focus groups in eight European countries

**Box S.1:** Extended protocol of the focus group

**Introduction**

- Purpose: to discuss what is important from the perspective of an individual with multiple health- and/or social problems in health/well-being and in care
- Overarching aim: the results of the discussion will be used to improve the care for persons with multiple health- and social problems
- Agenda explanation
- 'Rules'
    - No right / wrong answers, only differing points of view
    - Not necessary to agree with others, but must listen respectfully as others share their views
    - Talk to one-another, researchers only have role as moderator in guiding the discussion
    - Privacy / anonymity
    - The discussion will be recorded, and notes will be made
    - Turn off phones
- Informed consent
- Introduction of participants to one-another

**Part 1: Defining good health/well-being and care**

Step i):

- When you think about your health and well-being: try to complete this sentence / thought: "For me, being in great health means…"
- When you think about the care that you receive, have received, or may receive in the future, try to complete this sentence / thought: "I'd be really satisfied with all of the care / the overall care that I receive, if…"

**Write this question on a large white board / flip-over*

- Provide examples if participants are having trouble understanding what you mean:
    - …being able to work full-time. (health)
    - …being able to spend time with family and friends. (health)
    - …being able to live for many more years. (health)
    - …not going to the doctor so often. (health)
    - …my doctor took more time to explain things to me. (care)
    - …my partner would always be allowed to join me and discuss with the doctors as well. (care)
    - …my care plan was discussed with me. (care)
    - …I had one overall written care plan. (care)
    - …I always knew I could contact one person in case of questions. (care)

**The researcher needs to ask triggering and thought-provoking questions here.*

**The discussion should be about health and well-being in general, not about care specifically (yet).*

**Be careful not to provide too many examples and to steer the answers in one direction.*

**The researcher leading the discussion will be writing these thoughts out on a white board / flip-over.*

**The researchers need to allow the participants to reflect on one-another's ideas.*

*Close the discussion with a final question: i.e., have we missed anything?*

Step ii):

- Once it seems as though all participants have said what is important to them, the researcher will go through the points on the board, and with the help of the participants try to extrapolate what these things mean, in order to go from **specific** examples to **general** outcomes**.** For example:
  - "For me, being in good health means being able to work full-time", does this mean:
    - Participating in society?
    - Being able to support one-self financially? → Self-sufficiency
    - Having social contacts?
    - Having a daily structure?
  - "For me, being in good health means not going to the doctor so often", does this mean:
    - Having no acute health problems?
    - Having the ability to deal with minor health problems on your own? (self-management)
  - "I'd be really satisfied with all of the care / the overall care that I receive, if my doctor took more time to explain things to me", does this mean:
    - Good communication?
    - Shared-decision making?
    - More/enough time?
  - "I'd be really satisfied with all of the care / the overall care that I receive, if my partner could always be allowed to join me and discuss with the doctors as well.", does this mean:
    - Understanding your personal situation?
    - Involvement of the informal caregiver?
    - Good communication?

*The researcher needs to write key words that come forth on large notecards and place them in the centre of the table for all participants to be able to see.*
*The researcher needs to ask triggering and thought-provoking questions here – what do the points mentioned in step 1 really mean? What does it come down to? What would it mean for the person sitting next to you? How can you generalise this so that it is applicable for others in the room? Try to distinguish between individual opinions and actual group consensus.*
*In asking further questions, try to focus on multi-morbidity – how is this specific to you as a person with multiple health- and/or social problems, would this be different if you had a single disease?*
*For each initial statement, multiple cards can be made.*
*Close the discussion with a final question: i.e., have we covered all elements?*

**Break**

*Place cards from part one onto the table, sort them by 'triple aim'*
*Sort through original 77 cards, and add cards to the table from these (refrain from overlap, choose card from part one in words of participants over literature-cards)*

For peer review only

**Part 2: Selecting most important concepts**

- Instructions: "In the discussion during part 1 you've come up with points that are important for you when it comes to defining health and care. As you saw, we've been trying to write this into more general terms on these cards."
- In our research team we've also been looking at concepts that are often mentioned in research/literature as being important, we've added these to the table as well."

**Explain the new concepts in case anything is unclear.*

- "We'd like to ask each of you to look at all of these cards and write down for yourself which 10 are most important to you. This can be a mix of health/well-being, care, and cost cards."
- "Now we'd like you all to discuss your top 10 and try to see whether you can reach a group consensus, and make a top 10 of cards at the centre of the table."

**Ask a participant to first put his/hers down or read his/her list first, through discussion see whether some kind of consensus can be reached.*

**Box S.2:** List of literature-based outcomes used on cards in part two

**Health / well-being**

- Biomedical outcomes: e.g., blood sugar, blood pressure, lung function, kidney function
- Life expectancy
- Maintaining independence
- Activities of daily living: e.g. eating, bathing
- Independence from medical aids: e.g., wheelchair, oxygen
- Energy and fatigue
- Pain and discomfort
- Disability
- Physical mobility: e.g., being able to walk
- Work ability: being physically and mentally able to work
- Cognitive functioning: e.g., thinking, memory, concentration and learning
- Anxiety and depression
- Stress
- Worrying
- Listless: no spirit, apathetic, indifferent
- Self-esteem: a good feeling of one-self, self-respect
- Respect from others
- Social relationships: staying in contact with family/friends/acquaintances
- Maintaining social status: in terms of 'societal class', 'occupation', how you think others look at you in society.
- Societal participation: e.g., volunteer work, club memberships, community activities
- Social support: emotional, instrumental and financial assistance from the social network
- Loneliness
- Investing in future health and wellbeing
- Self-efficacy: belief in one's capacity to follow a needed or desired course of action
- Positive frame of mind, resilience: ability to maintain a positive perspective regarding the future, despite setbacks or change
- Coping
- Being able to deal with problems, losses, adverse events in an effective manner

For peer review only

| |
|---|
| • Health literacy: capacity to obtain, process, and understand information about illness and health<br>• Compliance/adherence to treatment<br>• Lifestyle: e.g., smoking, nutrition, physical activity, alcohol and drug use<br>• Feeling safe<br>• Enjoyment of life<br>• Maintaining dignity<br>• Living comfortably<br>• Caregiver burden |
| **Experience with care** |
| • Holistic assessment<br>• Shared decision-making<br>• Tailored care<br>• Individualised care planning<br>• Pro-active, prevention-oriented care<br>• Clear responsibilities and accountability<br>• Named coordinator<br>• Team work and collaboration between professionals<br>• Good communication<br>• Shared information between professionals and providers<br>• Continuity of care: pertains to continuity (e.g., follow-up, monitoring, stability, transfer) throughout time and between professionals and organisations)<br>• Confidence in knowledge and skills in professionals<br>• Respectful interaction between provider-patient: feeling of being treated with respect and being 'heard'<br>• Geographical access: e.g., facilities close to home<br>• Physical access: e.g., building is wheelchair accessible<br>• Timely access: e.g., no/short waiting lists<br>• Satisfaction with care process |
| **Costs** |
| • Service and support coverage: co-payments/deductible and financial access to services<br>• Travel and parking costs<br>• Loss of income – patient<br>• Loss of income - informal caregiver |

For peer review only

**Box S.3:** Most important concepts in health/well-being, care, and costs per focus group participant per country (part 2 of the focus groups)

| | # / 58 | Austria | Croatia | Germany[1] | Hungary | Netherlands | Norway | Spain | UK |
|---|---|---|---|---|---|---|---|---|---|
| *Health and well-being* | | | | | | | | | |
| **Physical well-being** | | | | | | | | | |
| Energy & fatigue | **12** | 4/7 | | | 1/6 | 4/7 | 2/7 | | 1/6 |
| Pain & discomfort | **4** | 3/7 | | | | | | | 1/6 |
| Biomedical outcomes | **3** | 2/7 | | | 1/6 | | | | |
| Disability | **5** | | 2/7 | | 1/6 | | 1/7 | | 1/6 |
| Progression free health state* | **2** | | | | 2/6 | | | | |
| Physical mobility | **5** | | 1/7 | 1/6 | 1/6 | 1/7 | 2/7 | | |
| Activities of daily living | **7** | | 1/7 | 3/6 | 1/6 | 1/7 | | | 1/6 |
| Life expectancy | **1** | | | | | 1/7 | | | |
| To be active* | **2** | | | | | | 2/7 | | |
| **Feeling safe** | **10** | | 1/7 | | 3/6 | 3/7 | | 3/6 | |
| Discrimination* | **3** | | 3/7 | | | | | | |
| **Cognitive functioning** | **12** | 4/7 | | 1/6 | | 2/7 | 1/7 | 3/6 | 1/6 |
| **Independence** | | | | | | | | | |
| Being able to choose what you do / want to do* | **1** | | | | | | | 1/6 | |
| Moving freely around street * | **1** | | | | | | | 1/6 | |
| Independence from medical aids | **3** | | | | 1/6 | | | 1/6 | 1/6 |
| Maintaining independence | **16** | | 2/7 | 5/6 | 1/6 | 1/7 | | 1/6 | 1/6 |
| Living comfortably | **5** | 1/7 | | 2/6 | | 1/7 | | | 1/6 |
| **Psychological well-being*** | **3** | | | 3/6 | | | | | |
| Listlessness | **3** | 2/7 | | | | | | 1/6 | |
| Loneliness | **2** | 2/7 | | | | | | | |
| Empathy* | **1** | | 1/7 | | | | | | |
| Stress/worrying | **7** | | | | 3/6 | | 1/7 | | 3/6 |
| Anxiety & depression | **2** | | | | 1/6 | | | | 1/6 |
| **Enjoyment of life** | **16** | 2/7 | | 4/6 | 1/6 | 3/7 | 3/7 | | 3/6 |
| **Positive frame of mind, resilience** | **16** | 2/7 | 3/7 | 2/6 | 2/6 | 1/7 | 2/7 | 2/6 | 2/6 |
| Creativity* | **2** | | 2/7 | | | | | | |
| Investing in future | **1** | | | | | | | 1/6 | |
| Coping | **5** | 1/7 | | | | | 2/7 | 1/6 | 1/6 |
| Maintaining dignity | **9** | 1/7 | | | 4/6 | | | 2/6 | 2/6 |
| Self-esteem | **11** | 1/7 | 1/7 | 3/6 | | 3/7 | 2/7 | | 1/6 |
| Acceptance* | **4** | | | 3/6 | | 1/7 | | | |
| Look out for possibilities* | **1** | | | | | | 1/7 | | |
| Hope | **1** | | | | | | 1/7 | | |

For peer review only

| | # / 58 | Austria | Croatia | Germany[1] | Hungary | Netherlands | Norway | Spain | UK |
|---|---|---|---|---|---|---|---|---|---|
| **Social relationships & participation** | | | | | | | | | |
| Social relationships | **17** | 3/7 | | 5/6 | 2/6 | 4/7 | | 2/6 | 1/6 |
| Societal participation | **12** | 1/7 | 3/7 | 3/6 | | 3/7 | | 1/6 | 1/6 |
| Respect from others | **6** | | | | | 2/7 | 2/7 | 1/6 | 1/6 |
| To be taken seriously* | **3** | | | | | | 3/7 | | |
| Social support | **5** | 1/7 | 1/7 | | | | 2/7 | 1/6 | |
| Being able to relate to other people without physical limits* | **1** | | | | | | | 1/6 | |
| Feeling useful* | **2** | | | | | | | 2/6 | |
| Get something off one's chest* | **2** | 2/7 | | | | | | | |
| (Maintaining good) social status | **5** | 2/7 | | 3/6 | | | | | |
| Not being reduced to the disease* | **6** | 1/7 | | | | | | | |
| To be regarded as whole human being* | | | | | | | 4/7 | | |
| Seeing past the outside/surface of a person* | | | | | | | 1/7 | | |
| Surrounding area takes care of someone's needs* | **1** | 1/7 | | | | | | | |
| Treatments compatible with work* | **1** | 1/7 | | | | | | | |
| Work ability | **6** | | 3/7 | | 1/6 | 2/7 | | | |
| Social expectations* | **1** | | 1/7 | | | | | | |
| Daily structure* | **4** | | | 4/6 | | | | | |
| Being able to plan* | **2** | | | | | | 2/7 | | |
| Burden for informal caregivers* | **3** | | | 2/6 | | | 1/7 | | |
| **Self-management*** | **7** | | | 6/6 | | 1/7 | | | |
| Lifestyle | **3** | 2/7 | | | | | | | 1/6 |
| Compliance/adherence to treatment | **5** | 1/7 | | 2/6 | 1/6 | | | | 1/6 |
| Self-efficacy | **6** | | 2/7 | | 1/6 | | | 1/6 | 2/6 |
| Investing in future health & well-being | **4** | | 1/7 | 2/6 | | 1/7 | | | |
| Health literacy | **7** | | | 5/6 | 1/6 | 1/7 | | | |
| **Daily quality of life** | **1** | | | | | | 1/7 | | |
| *Experience* | | | | | | | | | |
| **Individualised care planning / tailored care** | **13** | 1/7 | | 4/6 | 1/6 | 2/7 | 4/7 | | 1/6 |
| **Holistic assessment/understanding** | **11** | 1/7 | 1/7 | 4/6 | 2/6 | 2/7 | | | 1/6 |
| **Good interactions providers-patients** | | | | | | | | | |
| Good communication | **14** | 1/7 | 1/7 | 2/6 | | 2/7 | 1/7 | 3/6 | 4/6 |
| Encourage patients* | **1** | | | | | | | 1/6 | |
| Psychological support* | **2** | | | 1/6 | | 1/7 | | | |
| Optimist reactions* | **1** | | | | | | | 1/6 | |
| Shared decision-making | **13** | 2/7 | | 4/6 | 1/6 | 1/7 | 1/7 | | 4/6 |
| Respectful interaction between provider-patient | **12** | 2/7 | 1/7 | 5/6 | | | | | 4/6 |
| Trust* | **7** | | | 3/6 | | 4/7 | | | |
| Predictability of care service delivery* (good info) | **1** | | | | 1/6 | | | | |
| Flow of information* | **2** | | | | | | 2/7 | | |

For peer review only

| | # / 58 | Austria | Croatia | Germany[1] | Hungary | Netherlands | Norway | Spain | UK |
|---|---|---|---|---|---|---|---|---|---|
| Being listened to and believed* | **2** | | | | | | | | 2/6 |
| **Good collaborations providers** | | | | | | | | | |
| Shared information between providers* | **10** | 2/7 | 1/7 | 1/6 | 2/6 | 2/7 | | 1/6 | 1/6 |
| Data transparency* | **3** | | | 3/6 | | | | | |
| Team work between providers | **10** | | 1/7 | | 2/6 | | 2/7 | 2/6 | 3/6 |
| Solidary distribution between funds* | **1** | 1/7 | | | | | | | |
| Clear responsibility and accountability | **5** | | 1/7 | 3/6 | | | | | 1/6 |
| Named coordinator | **7** | | 1/7 | 4/6 | | 1/7 | 1/7 | | |
| **Continuity of care** | **9** | | 2/7 | 2/6 | 3/6 | 1/7 | | 1/6 | |
| Collaboration across sectors* | **5** | | | 4/6 | | | | | 1/6 |
| Coordination between primary care and hospitals* | **1** | | | | | | | 1/6 | |
| Prevent and avoid going to ER* | **2** | | | | | | | 2/6 | |
| Administrative barriers* | **1** | | 1/7 | | | | | | |
| Transferring of responsibility* | **1** | | 1/7 | | | | | | |
| Transfer-care* | **2** | | | 1/6 | | 1/7 | | | |
| After-care* | **2** | | | 1/6 | | 1/7 | | | |
| Systematic operating procedures in care* | **3** | | | | 3/6 | | | | |
| Utilisation of EMRs* | **2** | | | 3/6 | 2/6 | 2/7 | | | |
| Careful use of patient files / privacy | **2** | | | 2/6 | | | | | |
| **Confidence in knowledge and skills in providers** | **10** | 1/7 | 2/7 | 3/6 | 2/6 | 1/7 | | 1/6 | |
| Education of health professionals* | **3** | | 3/7 | | | | | | |
| Importance and function of centres of excellence* | **1** | | 1/7 | | | | | | |
| Broader specialisations* | **1** | | | | | | 1/7 | | |
| **Access & availability** | | | | | | | | | |
| Physical access | **5** | | 1/7 | 1/6 | | 2/7 | | 1/6 | |
| Timely access* (i.e., short waiting times) | **9** | | 2/7 | 1/6 | 2/6 | 1/7 | | | 4/6 |
| Enough time* (provider for patient) | **8** | 4/7 | | | 1/6 | 1/7 | | 2/6 | |
| Being seen by specialist when needed* | **2** | | | | | | | 2/6 | |
| Geographical access | **3** | | 1/7 | | | | | 1/6 | 1/6 |
| Availability of treatment* | **3** | | 3/7 | | | | | | |
| Home care (available) | **1** | | | 1/6 | | | | | |
| Transport* | **1** | | | 1/6 | | | | | |
| **Avoid unnecessary treatment*** | **2** | 2/7 | | | | | | | |
| **Pro-active, prevention-oriented, care** | **13** | | 1/7 | 1/6 | 4/6 | 4/7 | | | 3/6 |
| **Patient-education and support (groups)** | | | | | | | | | |
| Integration of self-help groups* | **3** | 3/7 | | | | | | | |
| Importance of associations of patients* | **1** | | 1/7 | | | | | | |
| Good trainings offered* | **1** | 1/7 | | | | | | | |
| Medication help* | **5** | | | 1/6 | | 4/7 | | | |
| Self-management support* | **1** | | | | | 1/7 | | | |

For peer review only

| | # / 58 | Austria | Croatia | Germany[1] | Hungary | Netherlands | Norway | Spain | UK |
|---|---|---|---|---|---|---|---|---|---|
| Good info for patients on health | **1** | | | | | | 1/7 | | |
| Family support* | **3** | | 3/7 | | | | | | |
| **Bureaucratic hurdles*** | **5** | | | 4/6 | | 1/7 | | | |
| **Physical surroundings** | | | | | | | | | |
| Material conditions in institutions* | **1** | | 1/7 | | | | | | |
| **Macro-level** | | | | | | | | | |
| Non-cooperation with health policy makers* | **2** | | 2/7 | | | | | | |
| Importance of media pressure | **2** | | 2/7 | | | | | | |
| Learning from different countries' experiences* | **1** | 1/7 | | | | | | | |
| **Overall satisfaction with care process** | **8** | 1/7 | | 4/6 | 2/6 | | | 1/6 | |
| *Costs* | | | | | | | | | |
| **Loss of income** | | | | | | | | | |
| Loss of income informal caregiver | **3** | | 1/7 | | | | | 2/6 | |
| Loss of income patient | **7** | 1/7 | 3/7 | | 1/6 | | | 1/6 | 1/6 |
| Overall costs in relation to income* | **1** | | | | | 1/7 | | | |
| **Service and support coverage** | **6** | 2/7 | 1/7 | 1/6 | 1/6 | | 1/7 | | |
| **Need for cost-benefit analysis*** | **2** | | 2/7 | | | | | | |
| **Money follows the patient*** (bundled type payment that crosses all care level-borders) | **1** | | 1/7 | | | | | | |
| **Travel and parking costs** | **2** | | | | 1/6 | | | | 1/6 |

Note: 1 = Group split into 2, so each time # out of 6 persons, each group discussed either health/well-being and costs or care and costs. Btw = between, Profs = professional care providers, EMRs = Electronic Medical Records, ER = Emergency Room. * Concepts/phrases that were novel and written on cards based on part 1 of the focus groups, and not on the basis of the a priori literature search.

For peer review only

**Box S.4:** Criteria used to come to a core set of outcomes to evaluate integrated care programmes

- **Cover the Triple Aim**: 1) improving population health/well-being and 2) patient experience, and 3) reducing cost (growth);
- **Measurable at an individual level** using PROMS and PREMS (this only applies to the first and second of the three aims);
- **Relevant for multi-morbidity** in different contexts and population groups;
- **Relevant for the 5P stakeholders: p**atients (persons with multi-morbidity), **p**artners (informal caregivers), **p**rofessionals, **p**ayers, **p**olicy makers.
- **Relevant across the** 17 selected **promising integrated care programmes**;
- **Timing of intervention effects**: the intervention effect (i.e., the impact of a programme on the outcome) in newly enrolled patients is likely to start occurring within the SELFIE evaluation period (6-18 months);
- The **outcomes are not redundant**, meaning that they are all necessary and not superfluous (e.g., one outcome does not overlap much with another outcome);
- **Preference independence**, i.e., the weight of one outcome can be elicited independently from the performance of another outcome;
- **Feasible to measure** performance.

For peer review only

## COREQ (COnsolidated criteria for REporting Qualitative research) Checklist

A checklist of items that should be included in reports of qualitative research. You must report the page number in your manuscript where you consider each of the items listed in this checklist. If you have not included this information, either revise your manuscript accordingly before submitting or note N/A.

| Topic | Item No. | Guide Questions/Description | Reported on Page No. |
|---|---|---|---|
| **Domain 1: Research team and reflexivity** | | | |
| *Personal characteristics* | | | |
| Interviewer/facilitator | 1 | Which author/s conducted the interview or focus group? | 8 |
| Credentials | 2 | What were the researcher's credentials? E.g. PhD, MD | 8 (1) |
| Occupation | 3 | What was their occupation at the time of the study? | 8 (1) |
| Gender | 4 | Was the researcher male or female? | 11 |
| Experience and training | 5 | What experience or training did the researcher have? | 8 |
| *Relationship with participants* | | | |
| Relationship established | 6 | Was a relationship established prior to study commencement? | 7 |
| Participant knowledge of the interviewer | 7 | What did the participants know about the researcher? e.g. personal goals, reasons for doing the research | 8 |
| Interviewer characteristics | 8 | What characteristics were reported about the inter viewer/facilitator? e.g. Bias, assumptions, reasons and interests in the research topic | 8, 11 |
| **Domain 2: Study design** | | | |
| *Theoretical framework* | | | |
| Methodological orientation and Theory | 9 | What methodological orientation was stated to underpin the study? e.g. grounded theory, discourse analysis, ethnography, phenomenology, content analysis | 9 |
| *Participant selection* | | | |
| Sampling | 10 | How were participants selected? e.g. purposive, convenience, consecutive, snowball | 7 |
| Method of approach | 11 | How were participants approached? e.g. face-to-face, telephone, mail, email | 7 |
| Sample size | 12 | How many participants were in the study? | 11 |
| Non-participation | 13 | How many people refused to participate or dropped out? Reasons? | 11 |
| *Setting* | | | |
| Setting of data collection | 14 | Where was the data collected? e.g. home, clinic, workplace | 7 |
| Presence of non-participants | 15 | Was anyone else present besides the participants and researchers? | 7, 11 |
| Description of sample | 16 | What are the important characteristics of the sample? e.g. demographic data, date | 7, 11 |
| *Data collection* | | | |
| Interview guide | 17 | Were questions, prompts, guides provided by the authors? Was it pilot tested? | 7-8 |
| Repeat interviews | 18 | Were repeat inter views carried out? If yes, how many? | 7-8 |
| Audio/visual recording | 19 | Did the research use audio or visual recording to collect the data? | 8 |
| Field notes | 20 | Were field notes made during and/or after the inter view or focus group? | 8-10 |
| Duration | 21 | What was the duration of the inter views or focus group? | 11 |
| Data saturation | 22 | Was data saturation discussed? | 9 |
| Transcripts returned | 23 | Were transcripts returned to participants for comment and/or | 9-10 |

For peer review only

| Topic | Item No. | Guide Questions/Description | Reported on Page No. |
|---|---|---|---|
| | | correction? | |
| **Domain 3: analysis and findings** | | | |
| *Data analysis* | | | |
| Number of data coders | 24 | How many data coders coded the data? | 9 |
| Description of the coding tree | 25 | Did authors provide a description of the coding tree? | 9 |
| Derivation of themes | 26 | Were themes identified in advance or derived from the data? | 9 |
| Software | 27 | What software, if applicable, was used to manage the data? | 9 |
| Participant checking | 28 | Did participants provide feedback on the findings? | 9-10 |
| *Reporting* | | | |
| Quotations presented | 29 | Were participant quotations presented to illustrate the themes/findings? Was each quotation identified? e.g. participant number | 9-10 (results) |
| Data and findings consistent | 30 | Was there consistency between the data presented and the findings? | 9-10 |
| Clarity of major themes | 31 | Were major themes clearly presented in the findings? | 9-10 |
| Clarity of minor themes | 32 | Is there a description of diverse cases or discussion of minor themes? | 9-10 |

Developed from: Tong A, Sainsbury P, Craig J. Consolidated criteria for reporting qualitative research (COREQ): a 32-item checklist for interviews and focus groups. *International Journal for Quality in Health Care*. 2007. Volume 19, Number 6: pp. 349 – 357

**Once you have completed this checklist, please save a copy and upload it as part of your submission. DO NOT include this checklist as part of the main manuscript document. It must be uploaded as a separate file.**

For peer review only
